# Canadian practice guidelines for the treatment of children and adolescents with eating disorders

**DOI:** 10.1186/s40337-020-0277-8

**Published:** 2020-02-01

**Authors:** Jennifer Couturier, Leanna Isserlin, Mark Norris, Wendy Spettigue, Melissa Brouwers, Melissa Kimber, Gail McVey, Cheryl Webb, Sheri Findlay, Neera Bhatnagar, Natasha Snelgrove, Amanda Ritsma, Wendy Preskow, Catherine Miller, Jennifer Coelho, Ahmed Boachie, Cathleen Steinegger, Rachel Loewen, Techiya Loewen, Elizabeth Waite, Catherine Ford, Kerry Bourret, Joanne Gusella, Josie Geller, Adele LaFrance, Anick LeClerc, Jennifer Scarborough, Seena Grewal, Monique Jericho, Gina Dimitropoulos, David Pilon

**Affiliations:** 1 McMaster University, Hamilton, Canada; 2 University of Ottawa, Ottawa, Canada; 3 University of Toronto, Toronto, Canada; 4 National Initiative for Eating Disorders, Toronto, Canada; 5 Canadian Mental Health Association – Waterloo, Wellington, Dufferin, Kitchener, Canada; 6 The Univeristy of British Columbia, Vancouver, Canada; 7 Patient advocate, Woodstock, Canada; 8 Parent advocate, Woodstock, Canada; 9 Ontario Ministry of Health and Long-Term Care, Toronto, Canada; 10 St. Joseph’s Care Group – Thunder Bay, Thunder Bay, Canada; 11 Dalhousie University, Halifax, Canada; 12 Laurentian University, Sudbury, Canada; 13 McMaster Children’s Hospital, Hamilton, Canada; 14 University of Calgary, Calgary, Canada

**Keywords:** Guidelines, Adolescent, Anorexia nervosa, Bulimia nervosa, Avoidant/restrictive food intake disorder

## Abstract

**Objectives:**

Eating disorders are common and serious conditions affecting up to 4% of the population. The mortality rate is high. Despite the seriousness and prevalence of eating disorders in children and adolescents, no Canadian practice guidelines exist to facilitate treatment decisions. This leaves clinicians without any guidance as to which treatment they should use. Our objective was to produce such a guideline.

**Methods:**

Using systematic review, the Grading of Recommendations Assessment, Development, and Evaluation (GRADE) system, and the assembly of a panel of diverse stakeholders from across the country, we developed high quality treatment guidelines that are focused on interventions for children and adolescents with eating disorders.

**Results:**

Strong recommendations were supported specifically in favour of Family-Based Treatment, and more generally in terms of least intensive treatment environment. Weak recommendations in favour of Multi-Family Therapy, Cognitive Behavioural Therapy, Adolescent Focused Psychotherapy, adjunctive Yoga and atypical antipsychotics were confirmed.

**Conclusions:**

Several gaps for future work were identified including enhanced research efforts on new primary and adjunctive treatments in order to address severe eating disorders and complex co-morbidities.

## Plain English summary

The objective of this project was to develop Canadian Practice Guidelines for the treatment of children and adolescents with eating disorders. We reviewed the literature for relevant studies, rated the quality of the scientific information within these studies, and then reviewed this information with a panel of clinicians, researchers, parents and those with lived experience from across the country. The panel came up with a list of recommendations regarding specific treatments. These recommendations included strong recommendations for the provision of Family-Based Treatment, as well as care provided in a least intensive environment. Weak recommendations were determined for Multi-Family Therapy, Cognitive Behavioural Therapy, Adolescent Focused Psychotherapy, adjunctive Yoga, and atypical antipsychotics. The panel also identified several areas for future research including the development of new treatments for severe and complex eating disorders.

## Introduction

Eating disorders are common and serious conditions affecting up to 4% of the population [1]. The mortality rate, particularly for Anorexia Nervosa (AN) is high [2, 3], and has been shown to increase by 5.6% for each decade that an individual remains ill [4, 5]. It is well-documented that interventions targeted at earlier stages of illness are critically important, given the evidence showing that earlier treatment leads to better outcomes [6, 7]. Despite the seriousness and prevalence of eating disorders in children and adolescents, no Canadian practice guidelines exist to facilitate treatment decisions. This leaves clinicians without any guidance as to which treatment they should use. We systematically reviewed and synthesized the knowledge available on treatments for children and adolescents with eating disorders to develop our guidelines.

### Review of existing guidelines

In the United States, practice parameters have been published by the American Academy of Child and Adolescent Psychiatry for youth with eating disorders [8]. These parameters reflect good clinical practice rather than making statements as to the strength of the evidence to support the recommendations. Clinical practice guidelines have also been developed by the National Institute of Health and Care Excellence [9], however, grading of the evidence is also not presented in these guidelines. The Academy for Eating Disorders has also published guidelines on their website that focus on medical management, but do not focus on psychotherapeutic/psychopharmacological interventions, nor the strength of the evidence (http://aedweb.org/web/downloads/Guide-English.pdf). In summary, guidelines that are currently available tend to focus on medical stabilization, and neglect psychotherapeutic/psychopharmacological approaches to treating eating disorders. Furthermore, they do not rate the strength of evidence. No Canadian guidelines focused on eating disorders in the pediatric age group exist.

### Objectives

Our aim was to synthesize the best available evidence on treatments for children and adolescents with eating disorders resulting in the production of a practice guideline. The research questions to drive this knowledge synthesis were discussed by our research team and guideline development panel, and are listed below.

### Research questions

What are the best treatments available for children and adolescents diagnosed with eating disorders?

- How effective is Family-Based Treatment for Anorexia Nervosa?
- How effective is Family-Based Treatment for Bulimia Nervosa?
- How effective is Cognitive Behavioural Therapy for Bulimia Nervosa?
- How effective is Dialectical Behaviour Therapy for Bulimia Nervosa?
- How effective are Atypical Antipsychotics for Anorexia Nervosa?
- How effective are Selective Serotonin Reuptake Inhibitors for Bulimia Nervosa?
- How effective is day treatment for any type of eating disorder?
- How effective is inpatient treatment for any type of eating disorder?

## Methods

### Overview

We used systematic review of the literature to arrive at a knowledge synthesis of the best treatments for children and adolescents with eating disorders. This was followed by a grading of the evidence using the Grading of Recommendations Assessment, Development, and Evaluation (GRADE) system [10–12]. These evidence profiles were then presented to a panel of stakeholders from across Canada, followed by a voting system and arrival at consensus on the recommendations. The Appraisal of Guidelines, Research, and Evaluation (AGREE II) tool was used to inform guideline development and reporting [13].

### Synthesis methods

#### Eligibility criteria

Following the principles outlined in the Cochrane Reviewer’s Handbook [14] and the Users’ Guides to Medical Literature [15], our inclusion criteria were:

A) Criteria pertaining to study validity: i) meta-analyses, randomized controlled trials, open trials, case series, and case reports,
B) Criteria pertaining to the subjects: i) involving children and adolescents (under age 18 years), ii) with eating disorders (Anorexia Nervosa, Bulimia Nervosa, Eating Disorder Not Otherwise Specified, Other Specified Feeding and Eating Disorder, Avoidant/Restrictive Food Intake Disorder, Binge Eating Disorder),
C) Criteria pertaining to the intervention: i) focusing on treatments including, but not limited to, Family-Based Treatment, Cognitive Behavioural Therapy, Dialectical Behavioural Therapy, Atypical Antipsychotics, Selective Serotonin Reuptake Inhibitors, Day Treatment, and Inpatient Treatment,
D) Criteria pertaining to the Outcome: i) weight (along with variants of weight such as BMI, treatment goal weight (TGW), etc.), ii) binge/purge frequency, iii) psychological symptoms such as drive for thinness, weight/shape preoccupation, and
E) Articles written in any language.

Exclusion criteria included: i) studies involving primarily adults (18 years or above), ii) studies focusing on medical management, iii) studies focusing on medical outcomes such as bone density, heart rate, iv) studies examining medical treatments such as hormone therapy, calcium, nutrition therapy, v) studies examining other medications. These exclusion criteria were developed for several reasons. We wanted to focus on treatments that were psychopharmacological and psychological in nature, along with outcomes that were central to the core features of eating disorders. We were trying to keep things as simple as possible when thinking of outcomes, especially with the goal of trying to combine studies in a narrative summary or even in a meta-analysis if possible. We focused on a couple of core outcomes with these goals in mind, so therefore excluded papers focusing on other physical outcomes (although these outcomes may indeed be related to weight status).

### Identifying potentially eligible studies

#### Databases

A literature search was completed using the following databases: Medline, PsycINFO, EMBASE, Cochrane Database of Systematic Reviews, Cochrane Central Register of Controlled Trials (CENTRAL) and CINAHL. The references of relevant articles obtained were also reviewed. This was an iterative process, such that search terms were added based on developing ideas and articles obtained.

#### Literature search strategy

Initially, an environmental scan of existing guidelines for children and adolescents with eating disorders was completed by the core research team using search terms “guidelines” and “eating disorders” in children and adolescents. Our library scientist then designed and executed comprehensive searches in the databases listed above to obtain evidence to align with each of the guideline questions. The searches included a combination of appropriate keyword and subject heading for each concept. The sample search strategy included, but was not limited to, various combinations of the following terms as appropriate for the questions being addressed: Anorexia nervosa OR bulimia nervosa OR eating disorder not otherwise specified OR other specified feeding and eating disorder OR avoidant/restrictive food intake disorder; AND family-based treatment OR cognitive behavioural therapy OR dialectical behavioural therapy OR atypical antipsychotics OR selective serotonin reuptake inhibitors OR day treatment OR day hospital OR inpatient treatment. The search string was developed further and was modified for each database as appropriate. The search strategy was completed in August 2016. The screening and reviewing process then ensued. Some treatments emerged as important through our search strategy that were not initially identified by our research team and guideline panel as interventions to evaluate. We later included these treatments through panel discussions.

#### Forward citation chaining

In November 2018 we used a forward citation chaining process to search each included article to see if it had been cited by any additional articles since August 2016 up until November 2018. We then screened the newly found articles to decide whether to include them. The forward chaining process involved the use of Google Scholar to locate all articles citing our included articles from the primary search.

#### Other strategies

Grey literature was also reviewed, including conference proceedings from the International Conference on Eating Disorders dating back the last 10 years (2008–2018). Databases of ongoing research were searched including The Cochrane Central Register of Controlled Trials (CENTRAL). We also hand searched the International Journal of Eating Disorders from the last 10 years for relevant articles (2008–2018).

### Applying eligibility criteria and extracting data

Two team members independently evaluated the results generated by our searches and came to consensus on which studies met eligibility criteria. We used the software Endnote and DistillerSR to organize our studies. DistillerSR was used for article screening and data extraction. Duplicate records identifying the same study were removed. Titles and abstracts were used to exclude obviously irrelevant reports by two reviewers. Potentially relevant articles were reviewed in full text by two reviewers who had to agree on inclusion, with a third resolving disputes. Authors of publications were contacted if any ambiguity existed about inclusion or exclusion. Data abstraction included the number of subjects, sex and/or gender of subjects, age range, type of treatment, type of control group if any, methodology (blinding, allocation concealment, intent-to-treat analysis), types of outcomes, and results. Sex was defined as biological sex, categorized into male or female. Gender was defined as the individual’s self-identified gender role/identity, categorized as girl, boy, or transgendered.

### Appraising studies

The Grading of Recommendations Assessment, Development, and Evaluation (GRADE) system explicitly describes how to rate the quality of each study, as well as how to synthesize the evidence and grade the strength of a recommendation [10–12]. Using this system, we developed an evidence profile of each included study that detailed all of the relevant data about the quality and strength of evidence for that particular study. Each evidence profile was created using GRADEpro software. We then used the GRADE system to synthesize and classify the overall quality of evidence for each intervention based on the quality of all of the studies using that intervention combined, taking into account risk of bias, inconsistency, indirectness, imprecision, publication bias, dose-response, and effect size. Although we looked at each outcome independently, when the rating of the evidence was the same, we collapsed the outcomes in the GRADEpro tables for the sake of efficiency.

### Guideline-related frameworks

The Appraisal of Guidelines, Research, and Evaluation (AGREE II) tool is an international standard of practice guideline evaluation that was used to inform our guideline development and reporting, and was developed by a co-author (MB) [13]. The Guideline Implementability for Decision Excellence Model (GUIDE-M) is a recent model that identifies factors to create recommendations that are optimally implementable [16]. We used these models to guide our methodological processes in the development of our practice guideline.

### The guideline team

The Guideline Team was comprised of a core research team and a larger guideline development panel (GDP). The core team presented the research questions to the GDP, reviewed evidence summaries, formulated practice recommendations, drafted the guideline, and limited biases that could impeach upon the guideline development process [17–19]. The chair of the GDP (MB) is an expert in guideline development having produced the AGREE framework [13]. She is a non-expert in the field of eating disorders, and as such, was an impartial chair. She led the consensus discussions of the GDP and she oversaw conflict-of-interest disclosures and management. A multidisciplinary GDP of 24 diverse stakeholders from across Canada was established including members from academic centres who are experts in the field of eating disorders, multi-disciplinary front-line clinicians/knowledge users from community settings, parent and patient representatives, hospital administrators, and policy-makers (all authors on this guideline).

### Procedures

An initial teleconference was held on May 18, 2016 with the core research team and the GDP to confirm the research questions prior to starting the systematic reviews. The initial teleconference oriented GDP members to the guideline development process, the roles and responsibilities of the GDP, as well as reviewed all conflicts of interest. The research questions were refined, the clinical population and outcomes were discussed, and the target audience reviewed.

Once the reviews were completed and the evidence profiles were generated, an in-person meeting was held at a central location on December 20, 2018. The core research team presented their evidence profiles for discussion with the GDP. The in-person meeting focused on a facilitated discussion of the evidence profiles and draft recommendations generated by the core team. For each question, the panel reviewed the evidence, and discussed: i) whether the interpretation of the evidence put forward by the core team aligned with that of the GDP, ii) strengths and limitations of the evidence base, iii) considerations of the generalizability of the studies, precision of the estimates, and whether the evidence aligned with values and preferences of Canadian patients and clinicians. Alternative interpretations and suggestions for further research were discussed. Minority or dissenting opinions were noted. Issues regarding implementability of the recommendations were considered, and suggestions for dissemination of the guideline were elicited.

Following the in-person meeting, GDP members were provided with the draft guidelines for review and approval. Group consensus on recommendations and strength of recommendations was obtained using a modified Delphi method [20], with voting by all GDP members using an anonymous web-based survey platform, Lime Survey (www.limesurvey.com). For a recommendation to be approved, at least 70% of the GDP were required to identify their agreement with the recommendation [12]. Consensus was achieved in the first round of voting. The GDP agreed to review and update the guideline every 5 years.

### External review

The purpose of the external review was to add validity to our guideline, but also initiate the dissemination process and elicit suggestions for dissemination and implementation. We invited review from four clinical and research experts in the area of pediatric eating disorders. Upon receiving external review, a summary of the review comments and suggestions was circulated to the GDP, along with a final version of the guideline for approval. The panel again discussed and voted on the changes suggested by the reviewers which included the addition of one further recommendation.

## Results

### Family therapy

**Fig. 1:**
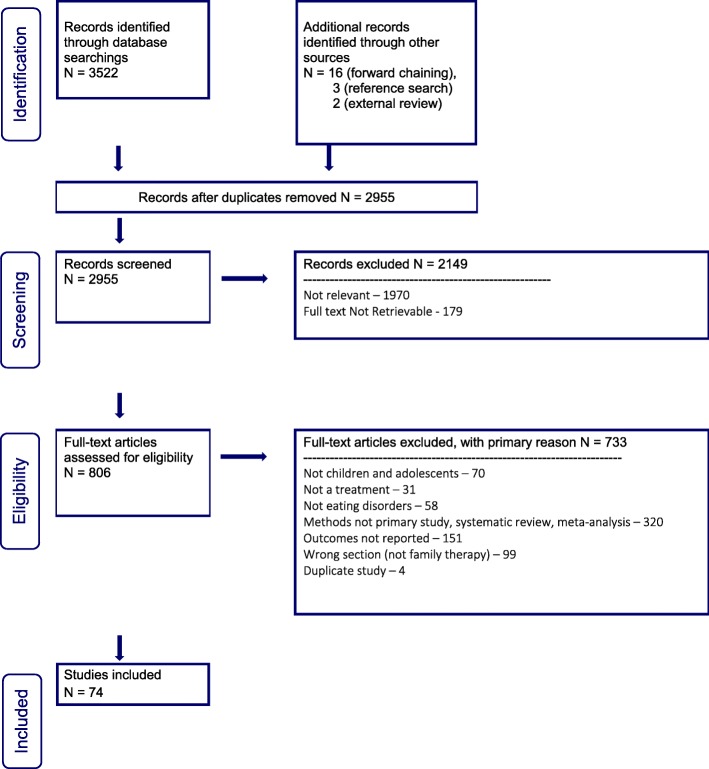
PRISMA flow diagram for family therapy

Three thousand, five hundred and twenty-two abstracts were identified for review within the family therapy section of our guideline (see PRISMA flow diagram, Fig. 1). Nineteen additional abstracts were identified through citation chaining (up to November 23, 2018) and review of reference lists. Two additional papers were identified through external review. After duplicates were removed, abstracts screened, and full text articles reviewed, 74 studies were included within the family therapy section of our guideline.

#### Family-based treatment

##### Anorexia nervosa

Of all treatments examined, Family-Based Treatment (FBT), in which parents are placed in charge of the refeeding process, had the most evidence to support its use in children and adolescents with Anorexia Nervosa (AN). One meta-analysis [21] and three high quality RCTs have demonstrated that greater weight gain and higher remission rates are achieved in FBT compared to individual treatment, especially when looking at 1 year follow up [6, 22, 23] (Table 1). One RCT compared a similar behavioural family systems therapy to Cognitive Behavioural Therapy (CBT) and found no significant differences [24], however the sample size was small (Table 1).

**Table 1:** Family-based treatment – anorexia nervosa

Certainty assessment							Impact	Certainty	Importance
№ of studies	Study design	Risk of bias	Inconsistency	Indirectness	Imprecision	Other considerations			
FBT vs supportive/dynamic individual– outcomes - Remission (assessed with: attaining target weight, good outcome category) Weight gain									
3	randomised trials	not serious	not serious	not serious	not serious	none	One meta-analysis indicated superiority of FBT at 6- and 12- month follow up. Three RCTs 43/90 (47.8%) with good outcome or in full remission with FBT, compared to 26/89 (29.2%) in Individual group. Total *n* = 179.	⨁⨁⨁⨁ HIGH	CRITICAL
		not serious	not serious	not serious	not serious	none	Weight gain greater in the FBT group compared to individual therapy group at end of treatment.	⨁⨁⨁⨁ HIGH	CRITICAL
RCT (FBT vs CBT) Remission/Good Outcome (assessed with: Morgan Russell Scale)									
1	randomised trials	not serious	not serious	not serious	not serious	none	7/13 (53.8%) had a good outcome in FBT group vs. 7/12 (58.3%) in the CBT group. No significant difference.	⨁⨁⨁⨁ HIGH	CRITICAL
Weight Gain (assessed with: kg and %IBW)									
1	Case control	serious ^b^	not serious	not serious	not serious	none	One case control retrospective chart review. 32 treated with FBT model compared to 14 in nonspecific therapy. Those in FBT made greater gains in weight.	⨁◯◯◯ VERY LOW	CRITICAL
Weight (assessed with: kg)									
7	Case series	very serious ^a,b^	not serious	not serious	not serious	none	7 large case series (total *n* = 223). Of these, 32 were children under age 13. Weight was significantly improved, pre to post.	⨁◯◯◯ VERY LOW	CRITICAL
Weight (assessed with: kg)									
11	Case reports	very serious ^a,b^	not serious	not serious	not serious	none	11 case reports detailing 29 patients who restored weight with FBT. Some described twins, comorbid conversion disorder, FBT within a group home setting, or FBT starting on a medical unit or use of FBT combined with medication.	⨁◯◯◯ VERY LOW	CRITICAL

Bibliography:

RCTs - Russell 1987 [6], Lock 2010 [23], Robin 1999 [22] (compared to psychodynamic individual)

RCT – Ball 2004 [24] (compared to CBT)

Case Control -Gusella 2017 [25]

Case Series - Paulson-Karlsson 2009 [26], Lock 2006 [27], Le Grange 2005 [28], Loeb 2007 [29], Goldstein 2016 [30], Couturier 2010 [31], Herscovici 1996 [32]

Case Reports – Le Grange 1999 [33], Le Grange 2003 [34], Loeb 2009 [35], Sim 2004 [36], Krautter 2004 [37], Aspen 2014 [38], Matthews 2016 [39], Turkiewicz 2010 [40], O’Neil 2012 [41], Duvvuri 2012 [42], Goldstein 2013[43]

In terms of nonrandomized studies, a case-control study of 34 patients treated with FBT compared to 14 treated with “nonspecific therapy” indicated that those in FBT made greater gains in body weight and were less likely to be hospitalized [25]. Seven case series (223 patients) also showed improvement in weight following treatment with FBT [26–32]. Eleven additional case reports (number of total patients = 29) are described showing benefit of FBT in terms of weight gain [33, 35–38, 40–44]. Some of these focus on twins [35, 42, 44], comorbid conversion disorder [43], FBT in a group home setting [38], FBT started on a medical unit [39], and FBT combined with medication [42].

Parent-Focused Family Therapy; a type of FBT in which most of the session is spent with the parents alone, may be just as effective as traditional FBT where the family is seen together [45–47] (Table 2).

**Table 2:** Parent focused FBT compared to standard FBT for children and adolescents with anorexia nervosa

Certainty assessment							Impact	Certainty	Importance
№ of studies	Study design	Risk of bias	Inconsistency	Indirectness	Imprecision	Other considerations			
Remission (assessed with: Weight greater than 95% and EDE score within 1 SD), Weight (kg), Psychological symptoms (EDI score)									
3	Randomized Trials	not serious	not serious	not serious	not serious	none	one RCT (*n* = 107) adolescents aged 12–18. Remission higher in Separated FBT (43% vs. 22%) compared to Standard FBT at end of treatment.	⨁⨁⨁⨁ HIGH	CRITICAL
		not serious	not serious	not serious	not serious	none	one RCT (*n* = 40), found no differences in weight outcome at end of treatment, except when subgroups analyzed. Those with high expressed emotion did better in separated family therapy in terms of weight gain. One pilot RCT (*n* = 18) found no differences in weight outcome at the end of treatment; both groups improved.	⨁⨁⨁⨁ HIGH	CRITICAL
		not serious	not serious	not serious	not serious	none	Improvement in EDI score was greater in the standard FBT group compared to the separated group. One pilot RCT (*n* = 18) found both groups improved in EAT scores with no difference between groups.	⨁⨁⨁⨁ HIGH	CRITICAL

Bibliography:

RCTs - Eisler 2000 [45], Le Grange 1992 [47], Le Grange 2016 [46]

##### Bulimia nervosa

Three high quality RCTs for Bulimia Nervosa (BN) have been completed and compared FBT to varying groups [48–50]. When FBT was compared to CBT, remission rates were significantly higher in the FBT group (39% versus 20%) [50]. Remission rates were also significantly better in the FBT group compared to supportive psychotherapy (39% versus 18% )[48]. However, when family therapy (with some elements consistent with FBT) was compared to guided self-help CBT, there were no significant differences (10% versus 14%) [49]. The adolescents in this study were slightly older and had the option to involve a “close other” rather than a parent, which may have resulted in lower remission rates. A case series and case report also support the use of FBT for BN [34, 51] (Table 3).

**Table 3:** Family-based treatment for bulimia nervosa

Certainty assessment							Impact	Certainty	Importance
№ of studies	Study design	Risk of bias	Inconsistency	Indirectness	Imprecision	Other considerations			
Remission (assessed with: Abstinence from binge or purge behaviour for 4 weeks) Psychological Symptoms (assessed with: EDE), Depression (assessed with: BDI),									
3	randomised trials	not serious	serious ^a,b,c^	not serious	not serious	none	one RCT (*n* = 130) compared FBT to CBT for adolescents with BN. FBT group achieved significantly higher remission rates (39% vs. 20%) at end of study. One RCT (n-85) compared FBT to CBT guided self care and found no difference in BP remission (although Binge alone was decreased in the CBT group). One RCT randomized 80 patients to FBT or supportive psychotherapy. 39% in FBT vs. 18% in supportive therapy were in remission at end of treatment; a significant difference.	⨁⨁⨁◯ MODERATE	CRITICAL
		not serious	serious ^a,b,c^	not serious	not serious	none	one RCT (*n* = 130) did not find any differences in EDE score at end of treatment for FBT vs. CBT for adolescents with BN. The other RCT (*n* = 80) also showed all EDE scores were more improved in the FBT group compared to supportive group.	⨁⨁⨁◯ MODERATE	CRITICAL
		not serious	serious ^a,b,c^	not serious	not serious	none	One RCT (*n* = 130) showed a decrease in depression scores that was greater in the FBT group compared to the CBT group at the end of the study. Another RCT (*n* = 80) did not show any differences in depression scores between FBT and supportive group.	⨁⨁⨁◯ MODERATE	CRITICAL
Binge Purge Frequency (assessed with: Frequency Scores)									
2	Case Reports	very serious ^d,e^	not serious	not serious	not serious	none	Two case reports of 9 patients in total describe decreases in binge and purge behaviours with FBT pre compared to post.	⨁⨁⨁◯ VERY LOW	CRITICAL

^a^one of three RCTs did not find a difference at end of treatment

^b^one RCT found a difference in psychological symptoms and the other did not

^c^one RCT showed a difference in depression scores and the other did not

^d^no randomization

^e^ no control condition

Bibliography:

RCTs – Le Grange 2015 [50], Le Grange 2007 [48], Schmidt 2007 [49]

Case Reports - Dodge 1995 [51], LeGrange 2003 [34]

#### Family-based treatment with other populations

Family-Based Treatment has been used for children and adolescents with atypical AN [52]. This case series of 42 adolescents who were not underweight but had lost a significant amount of weight, indicated that there were significant improvements in eating disorder and depressive symptoms, but no improvement in self-esteem (Table 4).

**Table 4:** Family-based treatment for other populations

Certainty assessment							Impact	Certainty	Importance
№ of studies	Study design	Risk of bias	Inconsistency	Indirectness	Imprecision	Other considerations			
Atypical AN - Depressive symptoms - Hughes 2017 (atypical AN) [52]									
1	Case series	very serious ^a,b^	not serious	not serious	not serious	none	Case series of 42 adolescents (age 12 to 18 years) with Atypical AN, that is adolescents who had lost a significant amount of weight, but were not currently underweight. There were significant decreases in eating disorder and depressive symptoms during FBT but no improvement in self esteem.	⨁◯◯◯ VERY LOW	CRITICAL
Case Reports - Spettigue 2018 [53], Murray 2012 [54] (ARFID)									
ARFID - Food Variety (assessed with: clinical impression), Weight									
2	Case Reports	very serious ^a,b^	not serious	not serious	not serious	none	Two case reports describe 7 cases in total (2 male, 5 female) in which ARFID was treated using a combination of FBT techniques, as well as some behavioural rewards and cognitive strategies. Food variety improved by clinical impression.	⨁◯◯◯ VERY LOW	CRITICAL
		very serious ^a,b^	not serious	not serious	not serious	none	Weight improved in all cases.	⨁◯◯◯ VERY LOW	CRITICAL
Case Report - Strandjord 2015 (transgendered youth) [55]									
Transgendered Youth -BMI									
1	Case Report	very serious ^a,b^	not serious	not serious	not serious	none	16 yo female sex assigned at birth treated with FBT to weight restoration then disclosed gender dysphoria with a desire to transition to male gender. BMI 14.9 before treatment, and 19 with treatment.	⨁◯◯◯ VERY LOW	CRITICAL

^a^no control condition

^b^no randomization

Two case reports describe the application of FBT for children with Avoidant/Restrictive Food Intake Disorder (ARFID) [53, 54]. These case reports (*n* = 7 cases total) indicate that weight improved in all cases (Table 4).

Family-Based Treatment and other family therapies for children and adolescents with eating disorders across the gender spectrum, including those who are gender variant or nonconforming requires more study. However, there is one case report describing the application of FBT with a transgendered youth, along with complexities that arose [55] (Table 4).

#### Adaptations to family-based treatment for anorexia nervosa

Adaptations to FBT, such as shorter or longer treatment [56], removal of the family meal [57], guided self-help [58], parent to parent consult [59], adaptive FBT involving extra sessions and another family meal [60], short term intensive formats [61, 62] and delivery of FBT by telehealth [63, 64], appear promising, but require more study (Table 5).

**Table 5:** FBT adaptations for children and adolescents with anorexia nervosa

Certainty assessment							Impact	Certainty	Importance
№ of studies	Study design	Risk of bias	Inconsistency	Indirectness	Imprecision	Other considerations			
Weight and Psychological Symptoms									
1	randomised trials 10 vs 20 sessions	not serious	not serious	not serious	not serious	none	RCT comparing 10 sessions of FBT to 20 sessions of FBT (*n* = 86). No differences in weight seen at 1 year. Those with nonintact families and severe eating related obsessive-compulsive features fair better in FBT.	⨁⨁⨁⨁ HIGH	IMPORTANT
		not serious	not serious	not serious	not serious	none	No differences in psychological symptoms (EDE) seen at 1 year. Those with nonintact families and severe eating related obsessive-compulsive features fair better in FBT.	⨁⨁⨁⨁ HIGH	IMPORTANT
1	randomised trials Adaptive vs. Standard FBT	not serious	not serious	not serious	not serious	none	45 adolescents in RCT comparing Adaptive FBT (3 extra sessions) to Standard FBT. No differences in outcomes in terms of weight.	⨁⨁⨁⨁ HIGH	CRITICAL
1	Randomized trial FBT +/− family meal	not serious	not serious	not serious	not serious	none	One RCT examined FBT with and without the family meal intervention (*n* = 23). No differences were found in weight at the end of the study.	⨁⨁⨁⨁ HIGH	CRITICAL
1	randomised trials FBT alone vs. FBT plus parent consultation	not serious	not serious	not serious	not serious	none	RCT of 20 adolescents aged 12–16 all female. 10 received FBT plus parent to parent consultation and 10 received FBT alone. Small increase in rate of weight restoration was seen in FBT plus consultation group.	⨁⨁⨁⨁ HIGH	CRITICAL
Weight									
4	Case Series guided self help, short term intensive, telemedicine	very serious ^a,b^	not serious	not serious	not serious	none	Uncontrolled feasibility study looked at Parental guided self help FBT for AN (*n* = 19). Improvement in weight was seen at the end of the study. Uncontrolled Short-Term Intensive Family Based Treatment for AN (*n* = 19). 18/19 patients gained and maintained weight. 30 month outcome of 74 patients treated with this Short Term Intensive Modal indicated 61% remained in full remission. One case series (*n* = 10) showing benefit of FBT delivery via telemedicine.	⨁◯◯◯ VERY LOW	CRITICAL
Weight									
1	Case Report telemedicine	very serious ^a,b^	not serious	not serious	not serious	none	One case report of FBT delivered by telehealth. Weight improved pre to post treatment.	⨁◯◯◯ VERY LOW	CRITICAL

Explanations

^a^no control condition

^b^no randomization

Bibliography:

RCT - Lock 2005 [56], Lock 2015 [60] Herscovici 2017 [57] Rhodes 2008 [59]

Case Series - Lock 2017 [58], Anderson 2017 [64], Marzola 2015 [62], Rockwell 2011 [61]

Case Report - Goldfield 2003 [63]

#### Adjuncts to family-based treatment for anorexia nervosa

Adjuncts to FBT, in which additional treatments have been added to FBT, such as cognitive remediation therapy versus art therapy [65], parental skills workshops [66] and Dialectical Behavioural Therapy (DBT) [67] for children and adolescents with AN show promise, but require further study (Table 6).

**Table 6:** FBT adjuncts for children and adolescents with anorexia nervosa

Certainty assessment							Impact	Certainty	Importance
№ of studies	Study design	Risk of bias	Inconsistency	Indirectness	Imprecision	Other considerations			
Psychological symptoms (EDE)									
1	randomised trials Art therapy vs. CRT	not serious	not serious	not serious	not serious	none	RCT examining FBT plus either Art Therapy or Cognitive Remediation Therapy. Global EDE score was slightly more improved in the Art Therapy Group (*p* < 0.03, *n* = 30).	⨁⨁⨁⨁ HIGH	CRITICAL
Weight Restoration (assessed with: Median BMI)									
1	Case control FBT +/−skills workshop	serious ^a^	not serious	not serious	not serious	none	One case control study described 45 families who had FBT with 45 families who had FBT plus a parent education and skills workshop. Week 4 weight gain was higher in those with the workshop, but there were no significant differences at the end of the study.	⨁◯◯◯ VERY LOW	CRITICAL
Weight (assessed with: pounds and %expected body weight)									
1	Case series DBT added	very serious ^a,b^	not serious	not serious	not serious	none	One case series (*n* = 11) of DBT added to FBT in a community-based clinic. 2/11 achieved full weight restoration at end of treatment	⨁◯◯◯ VERY LOW	CRITICAL
		very serious ^a,b^	not serious	not serious	not serious	none	6/11 had normal EDE scores at the end of the study.	⨁◯◯◯ VERY LOW	CRITICAL
Weight									
2	Case reports Emotion coaching	very serious ^a,b^	not serious	not serious	not serious	none	Two case reports of two patients with AN (one male) treated with adjunctive emotion coaching and the other with Attachment Based Family Therapy during a course of FBT. Both improved in weight to be fully weight restored.	⨁◯◯◯ VERY LOW	CRITICAL

^a^no randomization

^b^no control condition

Bibliography:

RCT – Lock 2018 [65]

Case Control – Ganci 2018 [66]

Case Series - Accurso 2018 [67]

Case Reports - Peterson 2016 [68], Wagner 2016 [69]

Two case reports describe the application of adjunctive emotion coaching and attachment based strategies to FBT for one male and one female patient with AN [68, 69] (Table 6).

Cognitive Behavioural Therapy has also been added as an adjunct to FBT for young patients with AN or BN. For AN, three case series [70–72] and two case reports [73, 74] indicate improved weight and psychological symptoms with added modules on perfectionism or exposure (Table 7). For BN, one case control study exists that compared one patient treated with FBT plus CBT to another patient treated with FBT alone, finding that both patients improved in terms of binge/purge symptoms and Eating Disorder Examination (EDE) scores [75] (Table 8).

**Table 7:** FBT plus CBT for children and adolescents with anorexia nervosa

Certainty assessment							Impact	Certainty	Importance
№ of studies	Study design	Risk of bias	Inconsistency	Indirectness	Imprecision	Other considerations			
Weight (assessed with: percent ideal body weight) Psychological Symptoms of ED (assessed with: EDE and EDI)									
3	Case series adding CBT to FBT	very serious ^a,b^	not serious	not serious	not serious	none	Total *n* = 78. Three case series looked at a perfectionism module added to FBT, or an exposure component to FBT. Weight increased significantly. One case series looked at Acceptance-Based Separated Family Treatment (*n* = 47), and also noted weight improved to ideal weight in about 50% of cases from pre to post treatment (20 sessions over 24 weeks).	⨁◯◯◯ VERY LOW	CRITICAL
		very serious ^a,b^	not serious	not serious	not serious	none	In one study 2/3 in full remission, 1/3 in partial remission.	⨁◯◯◯ VERY LOW	CRITICAL
		very serious ^a,b^	not serious	not serious	not serious	none	Decreases in EDE scores and EDI scores reported.	⨁◯◯◯ VERY LOW	CRITICAL
Perfectionism (assessed with: Child and Adolescent Perfectionism Scale)									
2	Case reports	very serious ^a,b^	not serious	not serious	not serious	none	Two case reports (*n* = 9 total) report on decreased perfectionism scores with the addition of a CBT perfectionism module or the addition of acceptance-based strategies	⨁◯◯◯ VERY LOW	IMPORTANT

Explanations

^a^no randomization

^b^no control condition

Bibliography:

Case Series - Hurst 2019 [72], Hildebrandt 2014 [70], Timko 2015 [71]

Case Reports - Hurst 2015 [74], Merwin 2013 [73]

**Table 8:** FBT plus CBT for children and adolescents with Bulimia Nervosa

Certainty assessment							Impact	Certainty	Importance
№ of studies	Study design	Risk of bias	Inconsistency	Indirectness	Imprecision	Other considerations			
Binge Purge Frequency (assessed with: frequency diary), Psychological symptoms (EDE)									
1	Case control	serious ^a^	not serious	not serious	not serious	none	One 15 yo female treated with FBT alone, compared to one 15 yo female treated with FBT and CBT (1 h sessions were split into 30 min of FBT and 30 min of CBT). Both improved significantly - BP episodes decreased from 10 to 12 episodes per week to 0.	⨁◯◯◯ VERY LOW	IMPORTANT
		serious ^a^	not serious	not serious	not serious	none	EDE scores were collected at end of this CBT plus FBT compared to FBT alone study (*n* = 2). EDE scores were similar in these two patients and demonstrated normal scores (in remission).	⨁◯◯◯ VERY LOW	IMPORTANT

Explanations

^a^no randomization

Bibliography:

Case Control - Hurst 2017 [75]

#### Multi-family therapy

One large high quality RCT (*n* = 169) found that Multi-Family Therapy (MFT) conferred additional benefits compared to single family therapy (FT) in terms of remission rates for adolescents with AN (75% in MFT versus 60% in FT), although no differences were found on the EDE [76]. There is one case control study examining MFT versus treatment as usual (TAU) in 50 female adolescents with AN [77]. Those in the MFT group had a higher percent body weight (99.6%) versus the TAU group (95.4%) at the end of the study. Two case series have also demonstrated a benefit of MFT for adolescents with AN [78, 79], and one case series with a mixed sample of adolescents with AN or BN showed benefit in psychological symptoms [80]. There is also one small case series examining MFT for adolescents with BN that found improvements in eating disorder symptoms [81] (Table 9).

**Table 9:** Multi family therapy for eating disorders

Certainty assessment							Impact	Certainty	Importance
DE	Study design	Risk of bias	Inconsistency	Indirectness	Imprecision	Other considerations			
Good Outcome at End of Treatment (assessed with: Morgan Russell Scale), Psychological Symptoms (EDE)									
1	randomised trials	not serious	not serious	not serious	not serious	none	RCT (*n* = 169) of adolescents with AN aged 11–18 comparing MFT to FBT (91% female). 65/86 (75.6%) good outcome at end of treatment in MFT versus 48/83 (57.8%) in the FBT group - significant difference. No differences between groups seen on the EDE. Both groups improved over time on the EDE.	⨁⨁⨁⨁ HIGH	CRITICAL
		not serious	not serious	not serious	not serious	none	No differences between groups seen on the EDE. Both groups improved over time on the EDE.	⨁⨁⨁⨁ HIGH	CRITICAL
Weight (assessed with: Percent ideal body weight)									
1	Case control	serious ^a^	not serious	not serious	not serious	none	Retrospective case control study looking at MFT versus TAU for AN. 50 female adolescents aged 11–18 were included (25 in MFT group and 25 in TAU group). Those in MFT restored weight to a higher percentage (99.6% vs. 95.4%).	⨁◯◯◯ VERY LOW	CRITICAL
Weight (assessed with: kg and BMI) Psychological Symptoms (assessed with: EDE, EDI)									
4	Case Series	very serious ^a,b^	not serious	not serious	not serious	none	Four studies without a control condition. Total *n* = 142 adolescents (5 males, 137 females), Diagnoses were mixed including AN, EDNOS and BN. Significant improvements in weight were reported.	⨁◯◯◯ VERY LOW	CRITICAL
		very serious ^a,b^	not serious	not serious	not serious	none	Improvements in psychological symptoms were seen pre to post MFT. In a case series of 10 adolescents aged 13 to 18 years, EDE scores decreased from 4.31 to 3.41 (cohen’s d 0.82).	⨁◯◯◯ VERY LOW	CRITICAL

^a^no randomization

^b^no control condition

Bibliography:

RCT – Eisler 2016 [76]

Case control - Gabel 2014 [77]

Case Series - Gelin 2015 [80], Hollesen 2013 [78], Salaminiou 2017 [79], Stewart 2015 [81]

#### Other forms of family therapy

Systemic Family therapy has been used in one RCT [82] and three case reports [83–85] for AN. The high quality RCT compared Systemic Family Therapy to FBT and found no significant differences in terms of remission rates, however, rate of weight gain was greater in the FBT group and the use of hospitalization was also significantly lower in the FBT group (Table 10). Structural Family Therapy has been studied within two case series [86, 87] and two case reports [88, 89]. Remission rates in the case series were 75% (38/51) by clinical impression (Table 11). Both of these types of family therapy (Systemic and Structural) might be helpful for children and adolescents with AN, but the evidence generally does not indicate superiority to FBT, especially when costs are taken into consideration.

**Table 10:** Systemic family therapy for anorexia nervosa

Certainty assessment							Impact	Certainty	Importance
№ of studies	Study design	Risk of bias	Inconsistency	Indirectness	Imprecision	Other considerations			
Systemic Family Therapy vs. FBT- Remission (assessed with: greater than 95% IBW)									
1	randomised trials	not serious	not serious	not serious	not serious	none	One RCT *n* = 164 (82 in each group, 141 were female). Remission rates were 27/82 in the FBT group and 21/82 in the Systemic Group - not significantly different.	⨁⨁⨁⨁ HIGH	CRITICAL
		not serious	not serious	not serious	not serious	none	Rate of weight gain were significantly faster in the FBT group compared to the Systemic Group.	⨁⨁⨁⨁ HIGH	CRITICAL
		not serious	not serious	not serious	not serious	none	No differences were seen in EDE score at end of treatment between FBT and Systemic Therapy	⨁⨁⨁⨁ HIGH	CRITICAL
Weight (assessed with: kg)									
3	Case Reports	very serious ^a,b^	not serious	not serious	not serious	none	Three case reports describe the use of systemic family therapy to good effect in terms of weight restoration. One case was a 14 yo male in which only the parents came to some of the sessions, another was a 15 yo female with comorbid osteosarcoma, and another is a 15 yo male.	⨁◯◯◯ VERY LOW	IMPORTANT

Explanations

^a^no control condition

^b^no randomization

Bibliography:

RCT - Agras 2014 [82]

Case Reports - Carr 1989 [83], De Benedetta 2011 [85], Merl 1989 [84]

**Table 11:** Structural family therapy for children and adolescents with Anorexia Nervosa

Certainty assessment							Impact	Certainty	Importance
№ of studies	Study design	Risk of bias	Inconsistency	Indirectness	Imprecision	Other considerations			
Recovery (assessed with: clinical impression), Weight Gain									
2	Case series	very serious ^a,b^	not serious	not serious	not serious	none	Two large case series of 51 female adolescents total used structural family therapy. 38/51 (75%) were deemed recovered by clinical impression.	⨁◯◯◯ VERY LOW	CRITICAL
		very serious ^a,b^	not serious	not serious	not serious	none	One of these case series reported between 5 and 31 kg of weight gain with the treatment (*n* = 25).	⨁◯◯◯ VERY LOW	CRITICAL
Weight Gain (assessed with: kg)									
2	Case reports	very serious ^a,b^	not serious	not serious	not serious	none	Two case reports (*n* = 2 both female) report weight restoration - one of these cases had co-morbid asthma.	⨁◯◯◯ VERY LOW	CRITICAL

Explanations

^a^no randomization

^b^no control condition

Bibliography:

Case Series - Minuchin 1975 [86], Wallin 2002 [87]

Case Reports - Combrinck-Graham 1974 [88], Liebman 1974 [89]

When looking at other nonspecific, family therapies in which family dynamics were examined, there is one high quality RCT which compared family therapy plus TAU to TAU alone [90] and three case reports [91–93] indicating a benefit of family therapy (Table 12). Family therapy has also been compared to family group psychoeducation with no significant differences in outcomes [94]. Both groups were recruited through an inpatient program. Both groups gained weight and were receiving other forms of treatment including medical monitoring and nutritional advice, in addition to the interventions of interest (Table 13).

**Table 12:** Family therapy (dynamic) for children and adolescents with Anorexia Nervosa

Certainty assessment							Impact	Certainty	Importance
№ of studies	Study design	Risk of bias	Inconsistency	Indirectness	Imprecision	Other considerations			
RCT - Good Outcome (assessed with: Morgan Russell)									
1	randomised trials	not serious	not serious	not serious	not serious	none	one RCT involving 60 adolescents randomized to TAU or TAU plus Family Therapy looking at family dynamics. 12/30 had a good outcome in the FT group compared to 5/30 in the TAU group (*p* < 0.05).	⨁⨁⨁⨁ HIGH	CRITICAL
Weight (assessed with: kg)									
3	Case Reports	very serious ^a,b^	not serious	not serious	not serious	none	three case reports looking at 4 female patients (one set of twins) treated with family therapy (one solution focused). Weight improved in all cases.	⨁◯◯◯ VERY LOW	IMPORTANT

Explanations

^a^no randomization

^b^no control group

Bibliography:

RCT - Godart 2012 [90]

Case Reports - Debow 1975 [91], Lane 1987 [92], O’Halloran 1999 [93]

**Table 13:** Family therapy compared to family group psychoeducation for adolescents with Anorexia Nervosa

Certainty assessment							Impact	Certainty	Importance
№ of studies	Study design	Risk of bias	Inconsistency	Indirectness	Imprecision	Other considerations			
Weight Restoration (assessed with: kg)									
1	randomised trials	not serious	not serious	not serious	not serious	none	No differences in weight restoration were seen at the end of the study between treatments. Both groups gained weight. (*n* = 25).	⨁⨁⨁⨁ HIGH	IMPORTANT

Bibliography:

Geist 2000 [94]

Emotion focused family therapy (EFFT) was compared in a randomized trial to CBT for 13 adolescents with BN [95] (Table 14). No differences were found in terms of binge/purge symptoms or psychological symptoms at the end of the study, however, the study was likely underpowered to detect differences.

**Table 14:** Emotion focused family therapy compared to cognitive behavioural therapy for children and adolescents with Bulimia Nervosa

Certainty assessment							Impact	Certainty	Importance
№ of studies	Study design	Risk of bias	Inconsistency	Indirectness	Imprecision	Other considerations			
Binge Purge Frequency (assessed with: frequency), Psychological Symptoms (assessed with: EDI)									
1	randomised trials	not serious	not serious	not serious	serious^a^	none	*n* = 13 adolescents with BN randomly assigned to EFFT or CBT. No differences in terms of binge purge frequency at end of study.	⨁⨁⨁◯ MODERATE	CRITICAL
		not serious	not serious	not serious	serious^a^	none	No differences in terms of psychological symptoms at end of study. Very small sample size.	⨁⨁⨁◯ MODERATE	CRITICAL

Explanations

^a^very small sample size

Bibliography:

RCT - Johnson 1998 [95]

### Individual and group outpatient psychotherapies

**Fig. 2:**
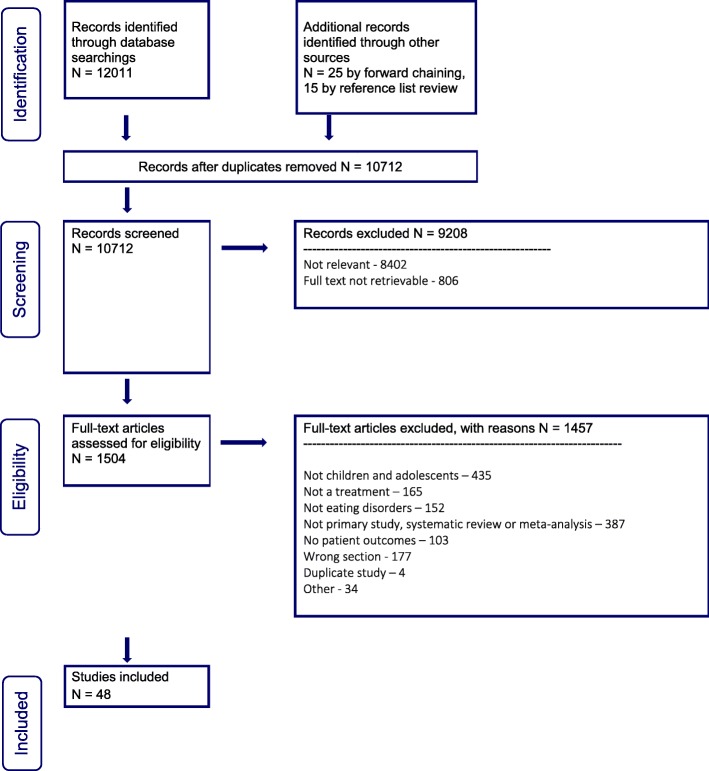
PRISMA flow diagram for individual and group psychotherapy

Twelve thousand and eleven abstracts were identified in our database searches for the individual and group psychotherapy section of our guideline (see PRISMA flow diagram, Fig. 2). Twenty-five were added with forward chaining up to November 21, 2018, and 15 more through reference list review. Nine thousand, two hundred and eight abstracts were excluded during the abstract screening phase, and a further 1457 were excluded based on full article review, leaving a total of 48 articles included.

#### Cognitive Behavioural therapy

##### Anorexia nervosa

A small RCT (*n* = 22) did not show any difference between CBT and Behavioural Family Therapy (similar to FBT) in terms of weight, or psychological symptoms on the EDE for children and adolescents with AN, however, both groups improved [24] (Table 15). One large case series [96] indicated that CBT resulted in weight gain and improvement in eating disorder psychological symptoms for children and adolescent with AN (*n* = 49). Eight additional case reports [97–104] support these results as well. Improvements have also been shown when CBT is delivered in a group setting for AN in a case control design involving 22 patients [105], and in a case series of 29 adolescents [106] (Table 16).

**Table 15:** Cognitive behavioural therapy for Anorexia Nervosa

Certainty assessment							Impact	Certainty	Importance
№ of studies	Study design	Risk of bias	Inconsistency	Indirectness	Imprecision	Other considerations			
Weight (assessed with: BMI), Psychological symptoms (EDE)									
1	randomised trials	not serious	not serious	not serious	not serious	none	RCT with 11 adolescents and young adults in CBT group compared to 11 in the Behavioural Family Therapy group (age range 13–23). There were no significant differences in terms of weight.	⨁⨁⨁⨁ HIGH	CRITICAL
		not serious	not serious	not serious	not serious	none	No differences seen in the eating psychopathology on the EDE between treatment groups.	⨁⨁⨁⨁ HIGH	CRITICAL
Weight (assessed with: kg), psychological symptoms with EDE									
1	Case Series	very serious ^a,b^	not serious	not serious	not serious	strong association	This was a large case series of 49 adolescents age 13 to 17 years, all female. 40 sessions weekly for 45 min. Weight was significantly increased by an average of 8.6 kg comparing pre to post weight.	⨁◯◯◯ VERY LOW	CRITICAL
		very serious ^a,b^	not serious	not serious	not serious	strong association	EDE scores were substantially decreased by a score of 2.03 (range 0–6) indicating psychological symptoms were much improved from pre to post treatment.	⨁◯◯◯ VERY LOW	CRITICAL
Weight (assessed with: kg), psychological symptoms									
8	Case Reports	very serious ^a,b^	not serious	not serious	not serious	none	8 case reports describing 8 adolescents (7 females, 1 male) with AN treated with CBT. One case had comorbid OCD. Weight improved with treatment. Age range 11.5 to 17 years.	⨁◯◯◯ VERY LOW	CRITICAL
		very serious ^a,b^	not serious	not serious	not serious	none	Improved EDE scores and EDI scores as well as improved eating behaviours in terms of a reduction in restricted eating.	⨁◯◯◯ VERY LOW	CRITICAL

Explanations

^a^no randomization

^b^no control condition

Bibliography:

RCT - Ball 2004 [24]

Case Series - Dalle Grave 2013 [96]

Case Reports - Cowdrey 2016 [97], Cooper 1984 [98], Martin-Murcia 2011 [99], Heffner 2002 [100], Scrignar 1971 [101], Fundudis 1986 [102], Ollendick 1979 [103], Wildes 2011 [104]

**Table 16:** Group cognitive behavioural therapy for children and adolescents with Anorexia Nervosa

Certainty assessment							Impact	Certainty	Importance
№ of studies	Study design	Risk of bias	Inconsistency	Indirectness	Imprecision	Other considerations			
Weight (assessed with: kg) Psychological Symptoms of ED (assessed with: EDE)									
1	Case Control	serious ^a^	not serious	not serious	not serious	none	This controlled study involved 11 adolescents in the CBT group condition compared to 11 adolescents in the treatment as usual condition. CBT group involved 24 sessions (90 min each) over a six-month period. There were no significant differences in terms of weight at the end of treatment.	⨁◯◯◯ VERY LOW	CRITICAL
		serious ^a^	not serious	not serious	not serious	none	Significant difference on the EDE subscale of Restraint (0.56 vs. 0.70 - clinical significance questionable).	⨁◯◯◯ VERY LOW	CRITICAL
Weight (assessed with: BMI) Psychological Symptoms of ED (assessed with: EDE)									
1	Case Series	very serious ^a,b^	not serious	not serious	not serious	none	Case series of 29 adolescent females (22 AN-R, 7 AN-BP). No control group. 40 sessions of group CBT over 40 weeks. Weight (BMI) improved pre to post treatment. EDE restraint and EDE weight concern improved Pre to Post treatment.	⨁◯◯◯ VERY LOW	CRITICAL

Explanations

^a^no randomization

^b^no control condition

Bibliography:

Case Control – Pegado 2018 [105]

Case Series - Ohmann 2013 [106]

##### Bulimia nervosa

For BN, three high quality RCTs were found examining CBT (Table 17). One RCT compared CBT to psychodynamic therapy in primarily adolescents, but also some young adults. This trial did not find any difference in terms of remission from BN. There were small differences in terms of a greater reduction in binge-purge frequency in the CBT group [107]. There were also two high quality RCTs identified comparing CBT to family-based approaches for BN [49, 50]. There are conflicting results between these two studies, with the study by Le Grange and colleagues [50] indicating significantly greater remission rates in the FBT group compared to the CBT group, whereas the study by Schmidt and colleagues [49] showed no significant difference between the groups with only a small proportion remitted in each group. Two large case series indicate significant decreases in binge-purge frequency pre to post treatment [108, 109]. Several case reports indicating improvement in binge-purge symptoms exist [110–114].

**Table 17:** Cognitive behavioural therapy for Bulimia Nervosa

Certainty assessment							Impact	Certainty	Importance
№ of studies	Study design	Risk of bias	Inconsistency	Indirectness	Imprecision	Other considerations			
CBT vs FBT - Remission (assessed with: abstinence from BP for 4 weeks)									
2	randomised trials	not serious	not serious	not serious	not serious	none	RCT *n* = 130 aged 12–18 years. 18 sessions over 6 months. 20% remitted in CBT group versus 39% remitted in FBT group (*p* < 0.04, NNT = 5). RCT *n* = 85 (guided self care CBT) remitted 6/44 in CBT group versus 4/41 in family group. no significant difference.	⨁⨁⨁⨁ HIGH	CRITICAL
CBT vs. Psychodynamic - Remission Rates (assessed with: Diagnostic Criteria)									
1	randomised trials	not serious	not serious	not serious	not serious	none	one RCT 81 females mean age 18.7 years (range 14–20). 33.3% remitted in the CBT group and 31.0% in the psychodynamic group. No significant differences. Mean of 37 sessions. Both groups improved, there were small between groups effect sizes for binge eating (d = 0.23) and purging (d = 0.26) in favour of CBT and for eating concern (d = 0.35) in favour of PDT.	⨁⨁⨁⨁ HIGH	CRITICAL
Binge Purge Behaviour (assessed with: EDE)									
2	Case Series	very serious ^a,b^	not serious	not serious	not serious	none	Two large case series (*n* = 68 including 2 males, 66 females, and *n* = 34 all female). Total age range 12–19. Number of sessions 16–20. Frequency of binge and purge episodes decreased significantly pre to post treatment.	⨁◯◯◯ VERY LOW	CRITICAL
		very serious ^a,b^	not serious	not serious	not serious	none	Case series of 68 adolescents - EDE significantly improved global EDE score from 3.6 to 1.8 from pre to post treatment.	⨁◯◯◯ VERY LOW	CRITICAL
Binge Purge Frequency (assessed with: Frequency), Psychological Symptoms (EDE or EAT)									
5	Case Reports	very serious ^a,b^	not serious	not serious	not serious	none	Case reports involving 9 patients in total. Frequency of binge and purge behaviours described as decreased.	⨁◯◯◯ VERY LOW	CRITICAL
		very serious ^a,b^	not serious	not serious	not serious	none	7 cases -EDE or EAT significantly improved.	⨁◯◯◯ VERY LOW	CRITICAL

Explanations

^a^no randomization

^b^no control condition

Bibliography:

RCT – Le Grange 2015 [50], Schmidt 2007 [49] (CBT vs. FBT) Stefini 2017 [107] (CBT vs. psychodynamic)

Case Series - Dalle Grave 2015 [108], Lock 2005 [109]

Case Reports – Schapman-Williams 2006 [110], Cooper 2007 [111], Anbar 2005 [112], Schapman-Williams 2007 [113], Sysko 2011 [114]

##### Avoidant/restrictive food intake disorder

There were 13 case reports identified in which CBT was used to treat ARFID [115–127]. One of these described the delivery of CBT by telemedicine [127]. One case described the combined treatment of CBT with fluoxetine for a significant choking phobia [120]. Although these reports are preliminary, improvements in food avoidance were noted in all cases (Table 18).

**Table 18:** Cognitive behavioural therapy for ARFID

Certainty assessment							Impact	Certainty	Importance
№ of studies	Study design	Risk of bias	Inconsistency	Indirectness	Imprecision	Other considerations			
Avoidance of Food (assessed with: clinical impression)									
12	Case Reports	very serious ^a,b^	not serious	not serious	not serious	none	28 cases are described in which various cognitive behavioural strategies including systematic desensitization (17), hypnosis (6) and EMDR (4) were used. Patients were aged 3 to 16 years (12 male, 16 female). Improvement in food avoidance behaviour was reported in all cases.	⨁◯◯◯ VERY LOW	IMPORTANT
Telemedicine - Increased food variety (assessed with: bites of nonpreferred food)									
1	Case Report	very serious ^a,b^	not serious	not serious	not serious	none	Case report with CBT delivered by teleconsultation to parents of 8 year old boy with ARFID. Increased frequency of bites of nonpreferred food was noted.	⨁◯◯◯ VERY LOW	IMPORTANT

Explanations

^a^no randomization

^b^no control condition

Bibliography:

Case Reports - Murphy 2016 [125], Fischer 2015 [124], Nock 2002 [119], Okada 2007 [122], Ciyiltepe 2006 [121], de Roos 2008 [123], Culbert 1996 [117], Siegel 1982 [115], Reid 2016 [126], Chatoor 1988 [116], Chorpita 1997 [118], Bloomfield 2018 [127], Bailly 2003 [120]

#### Adolescent focused psychotherapy

##### Anorexia nervosa

Adolescent Focused Psychotherapy (AFP: based on psychodynamic principles) [22, 23, 128] and other psychodynamic treatments [129] have some evidence to support their use (Table 19). Remission rates were not significantly different between AFP and FBT in two RCTs involving a total sample of 158 adolescents with AN [22, 23]. Rates of 20% (12/60) remitted in AFP compared to 34% (21/60) in FBT were found in a study by Lock and colleagues [23], whereas 41% in the AFP group met the weight goal of the 50th percentile in a study by Robin and colleagues [22] compared to 53% in the FBT group. Differences between AFP and FBT became more apparent at 1 year follow-up with FBT demonstrating an advantage [23]. Group analytic psychotherapy also has some evidence to support its use for AN [130] (Table 20). Psychodynamic Therapy (group or individual) for AN may be beneficial, however other treatments have some advantages over psychodynamic therapy in terms of cost and more rapid improvement in symptoms.

**Table 19:** Adolescent focused psychotherapy/psychodynamic for Anorexia Nervosa

Certainty assessment							Impact	Certainty	Importance
№ of studies	Study design	Risk of bias	Inconsistency	Indirectness	Imprecision	Other considerations			
Remission (assessed with: normal weight and EDE score)									
2	randomised trials	not serious	not serious	not serious	not serious	none	RCT of Adolescent Focused Psychotherapy versus FBT (*n* = 121, 11male, 110 female, age 12–18). 12/60 (20%) remitted at end of treatment in AFT group versus 21/61 (34.4%) in FBT group. No significant differences in terms of remission. No differences in remission in another RCT (*n* = 37). 52.6% in FBT reached 50th percentile weight vs. 41.2 in individual (*p* < 0.05).	⨁⨁⨁⨁ HIGH	CRITICAL
		not serious	not serious	not serious	not serious	none	Those in FBT had greater change on EDE scores at end of treatment.	⨁⨁⨁⨁ HIGH	CRITICAL
Weight									
2	Case Reports	very serious ^a,b^	not serious	not serious	not serious	none	Two case reports describing three cases total (age 12–16 years, all female) in which psychodynamic therapy over 1–2 years of therapy resulted in weight restoration.	⨁◯◯◯ VERY LOW	CRITICAL

Explanations

^a^no control condition

^b^no randomization

Bibliography:

RCT - Lock 2010 [23], Robin 1999 [22]

Case Reports - Fitzpatrick 2010 [128], Pharis 1984 [129]

**Table 20:** Group analytic therapy for children and adolescents with AN and BN

Certainty assessment							Impact	Certainty	Importance
№ of studies	Study design	Risk of bias	Inconsistency	Indirectness	Imprecision	Other considerations			
Psychological Symptoms (assessed with: EDI, SEED-short evaluation eating disorders)									
1	Case Reports	very serious ^a^	not serious	not serious	not serious	none	8 female adolescents aged 15–17 (3 with AN, 5 with BN). SEED AN and EDI maturity fears significantly decreased from pre to post. Setting was outpatient - 2 years 1.5 h per week	⨁◯◯◯ VERY LOW	IMPORTANT

Explanations

^a^no control condition

Bibliography:

Case Report - Prestano 2008 [130]

#### Dialectical Behavioural therapy

Dialectical Behavioural Therapy (DBT) for eating disorders has been applied for youth with AN, BN, Eating Disorder Not Otherwise Specified (EDNOS) and Binge Eating Disorder (BED) with promising results [131–133]. Two case series report decreases in binge-purge symptoms, and improvements in psychological eating disorder symptoms [131, 133], along with reductions in frequency of self-harm in multi-diagnostic youth [131] (Table 21).

**Table 21:** Dialectical behavioural therapy for eating disorders

Certainty assessment							Impact	Certainty	Importance
№ of studies	Study design	Risk of bias	Inconsistency	Indirectness	Imprecision	Other considerations			
Binge Frequency (assessed with: number per month) Purge Frequency									
2	Case Series	very serious ^a^	not serious	not serious	not serious	none	Two case series and one case report for a total of 22 patients (10 EDNOS, 6 AN, 6 BN) reported a significant decrease in binge frequency. Reduction in vomiting pre and post treatment.	⨁◯◯◯ VERY LOW	IMPORTANT
		very serious ^a^	not serious	not serious	not serious	none	There were decreases in psychological symptoms.	⨁◯◯◯ VERY LOW	IMPORTANT
		very serious ^a^	not serious	not serious	not serious	none	A decrease in self harm also noted.	⨁◯◯◯ VERY LOW	IMPORTANT
Binge Frequency, EDE scores									
1	Case Report	very serious ^a^	not serious	not serious	not serious	none	*N* = 1 female with BED – decreased frequency of binge episodes	⨁◯◯◯ VERY LOW	IMPORTANT
		very serious ^a^	not serious	not serious	not serious	none	improvement in EDE scores.	⨁◯◯◯ VERY LOW	IMPORTANT

Explanations

^a^no control group

Bibliography:

Case Series – Salbach-Andrae 2008 [133], Fischer 2015 [131]

Case Report - Safer 2007 [132]

#### Adjunctive treatments

Cognitive Remediation Therapy (CRT) has been mentioned in the family therapy section of this guideline as an adjunct to FBT [65], however, it has also been studied as an adjunct to other therapies in a case series [134] and a case report [135] for AN (Table 22). It has been used in multiple settings and will be touched upon again within the level of care section of this guideline.

**Table 22:** Cognitive remediation therapy for children and adolescents with Anorexia Nervosa

Certainty assessment							Impact	Certainty	Importance
№ of studies	Study design	Risk of bias	Inconsistency	Indirectness	Imprecision	Other considerations			
ART vs. CRT - Weight (assessed with: BMI), ED symptoms, depression, anxiety									
1	randomised trials	not serious	not serious	not serious	not serious	none	RCT comparing Art Therapy and CRT (both receiving FBT) *n* = 30 (3 male, 27 female). BMI not significantly different.	⨁⨁⨁⨁ HIGH	CRITICAL
		not serious	not serious	not serious	not serious	none	Art Therapy significantly better than CRT in terms of global EDE score at the end of 15 sessions.	⨁⨁⨁⨁ HIGH	CRITICAL
		not serious	not serious	not serious	not serious	none	No difference between CRT and Art Therapy with respect to depression scores.	⨁⨁⨁⨁ HIGH	CRITICAL
		not serious	not serious	not serious	not serious	none	No difference between CRT and Art Therapy with respect to Anxiety scores	⨁⨁⨁⨁ HIGH	CRITICAL
Weight (assessed with: BMI), Depression (BDI), Anxiety (STAI)									
1	Case Series	very serious ^a,b^	not serious	not serious	not serious	none	One open trial of 20 patients (10 inpatients, 10 outpatients) describes weight improvement with 10 sessions of CRT. Open trial was pre post CRT.	⨁◯◯◯ VERY LOW	CRITICAL
		very serious ^a,b^	not serious	not serious	not serious	none	Depression scores decreased significantly following CRT (pre compared to post)	⨁◯◯◯ VERY LOW	CRITICAL
		very serious ^a,b^	not serious	not serious	not serious	none	No differences pre and post were seen in terms of Anxiety.	⨁◯◯◯ VERY LOW	CRITICAL
Weight									
1	Case Report	very serious ^a,b^	not serious	not serious	not serious	none	Case report – 12 year old female with AN - pre post and 7 month follow up after 10 sessions CRT. Weight improved at the follow up assessment to a healthy weight range.	⨁◯◯◯ VERY LOW	IMPORTANT

Explanations

^a^no control group

^b^no randomization

Bibliography:

RCT - Lock 2018 [65]

Case Series -Dahlgren 2013 [134]

Case Report - van Noort 2015 [135]

One high quality study suggests some benefits of adjunctive yoga in terms of psychological symptoms of eating disorders, as well as depression and anxiety [136]. In this study, 50 girls and 4 boys were randomly assigned to an 8-week trial of yoga plus standard care versus standard care alone. The majority of the participants had AN (29/54), and others were diagnosed with BN (9/54) and EDNOS (15/54). Eating disorder symptoms measured by the EDE decreased more significantly in the yoga group. Both groups demonstrated maintenance of body mass index (BMI), along with decreases in anxiety and depression scores (Table 23).

**Table 23:** Yoga for eating disorders

Certainty assessment							Impact	Certainty	Importance
№ of studies	Study design	Risk of bias	Inconsistency	Indirectness	Imprecision	Other considerations			
Psychological Symptoms (assessed with: EDE), weight, anxiety, depression									
1	randomised trials	not serious	not serious	not serious	not serious	none	In this RCT 50 girls and 4 boys were randomized to yoga plus standard treatment, or standard treatment alone.. There were no differences in weight between the yoga group and the no yoga group at the end of the study.	⨁⨁⨁⨁ HIGH	CRITICAL
		not serious	not serious	not serious	not serious	none	The yoga group demonstrated greater decreases in EDE score at 12 weeks.	⨁⨁⨁⨁ HIGH	CRITICAL
		not serious	not serious	not serious	not serious	none	Anxiety scores improved over time in the yoga group and were significantly improved compared to the no yoga group.	⨁⨁⨁⨁ HIGH	CRITICAL
		not serious	not serious	not serious	not serious	none	Depression scores were significantly improved in the yoga group compared to the control group.	⨁⨁⨁⨁ HIGH	CRITICAL

Bibliography:

RCT - Carei 2010 [136]

### Medications

#### Atypical antipsychotics

**Fig. 3:**
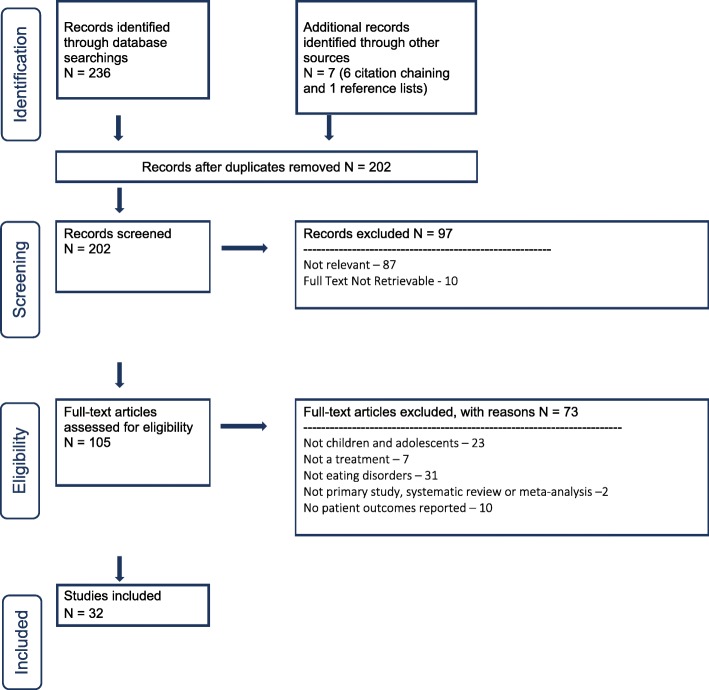
PRISMA flow diagram for antipsychotics

Two hundred and thirty-six abstracts were identified through database searching for the atypical antipsychotic section of our guideline (see PRISMA flow diagram Fig. 3). Seven additional articles were found through citation chaining and reference list review. After excluding 97 abstracts and then excluding 73 full text articles we arrived at 32 included studies for the atypical antipsychotic section. We then divided up the antipsychotics into their respective categories – Olanzapine, Risperidone, Quetiapine, and Aripiprazole.

#### Olanzapine

##### Anorexia nervosa

Olanzapine has been the most commonly studied psychotropic medication for children and adolescents with AN (Table 24). At present, only one double blind placebo-controlled trial in this population has been published. Kafantaris and colleagues [137] examined olanzapine in 20 underweight adolescents being treated in inpatient (*n* = 9), day treatment (*n* = 6) and outpatient (*n* = 5) settings (age range 12.3 to 21.8 years). In a 10-week pilot study, they found no differences in beneficial effect between the olanzapine and placebo groups in the 15 subjects who completed the trial; however, the treated group showed a trend towards increasing fasting glucose and insulin levels by the end of the study. The mean dose of olanzapine was 8.5 mg daily. Of note, only 21% of eligible patients were recruited into the study and there was a high attrition rate. Although other research teams have also attempted RCTs using olanzapine in this population, trials have been hampered by a myriad of confounding and recruitment issues [155].

**Table 24:** Olanzapine for children and adolescents with Anorexia Nervosa

Certainty assessment							Impact	Certainty	Importance
№ of studies	Study design	Risk of bias	Inconsistency	Indirectness	Imprecision	Other considerations			
Weight (assessed with: BMI), Psychological Symptoms, Side Effects									
1	randomised trials	not serious	not serious	not serious	not serious	none	RCT with 10 subjects in olanzapine group and 10 in placebo group. No differences were found between groups in rate of weight restoration or final weight. Difference in BMI was 0.4 kg/m2 and was not significant. Mean dose was 8.5 mg/day.	⨁⨁⨁⨁ HIGH	CRITICAL
		not serious	not serious	not serious	not serious	none	No differences in eating disorder symptoms or psychological functioning.	⨁⨁⨁⨁ HIGH	CRITICAL
		not serious	not serious	not serious	not serious	none	A trend of increasing fasting glucose and insulin levels were found in the olanzapine group.	⨁⨁⨁⨁ HIGH	CRITICAL
Weight gain, activity levels, side effects									
3	Case Control	serious ^a^	not serious	not serious ^a^	not serious	none	There are three non randomized case control studies. One of the studies found the rate of weight gain was greater in the olanzapine group, while another study found no differences between cases and controls in terms of weight gain.	⨁◯◯◯ VERY LOW	CRITICAL
		serious ^a^	not serious	not serious ^a^	not serious	none	Reduced activity levels were observed in one study.	⨁◯◯◯ VERY LOW	CRITICAL
		serious ^a^	not serious	not serious ^a^	not serious	none	Sedation and dyslipidemia was found in 56% of patients in one study. One study found that 32% of patients discontinued the treatment due to a side effect.	⨁◯◯◯ VERY LOW	CRITICAL
Weight, hyperactivity, side effects									
2	Case Series	very serious ^a^	not serious	not serious	not serious	none	60 patients total involved in these two case series. Improvements in weight noted.	⨁◯◯◯ VERY LOW	CRITICAL
		very serious ^a^	not serious	not serious	not serious	none	Improvements in hyperactivity are noted.	⨁◯◯◯ VERY LOW	CRITICAL
		very serious ^a^	not serious	not serious	not serious	none	No long term adverse effects were seen 3 months after discontinuing medication.	⨁◯◯◯ VERY LOW	CRITICAL
Weight, side effects									
13	Case Reports	very serious ^a^	not serious	not serious	not serious	none	Thirteen studies report on 30 cases. All studies report improvement in weight.	⨁◯◯◯ VERY LOW	CRITICAL
		very serious ^a^	not serious	not serious	not serious	none	One case study reports on QTc prolongation (a problem on the ECG), another reports a case with neuroleptic malignant syndrome.	⨁◯◯◯ VERY LOW	CRITICAL

Explanations

^a^observational study, non randomized

Bibliography:

RCT - Kafantaris 2011 [137]

Case Control - Spettigue 2018 [138], Norris 2011 [139], Hillebrand 2005 [140]

Case Series -Swenne 2011 [141], Leggero 2010 [142]

Case Reports - Pisano 2014 [143], Duvvuri 2012 [42], Dennis 2006 [144], Boachie 2003 [145], Mehler 2001 [146], La Via 2000 [147], Dadic-Hero 2009 [148], Hein 2010 [149], Tateno 2008 [150], Ercan 2003 [151], Dodig-Curkovic 2010 [152], Ayyildiz 2016 [153], Ritchie 2009 [154]

Three case control studies have examined the use of olanzapine in children and adolescents with AN [138–140]. The most recent of these studies enrolled 38 patients with AN; 22 of whom took olanzapine and 10 who declined medication and were retained as a comparison group [138]. The mean dose of medication was 5.28 mg daily over a 12-week trial period. Those in the medication group demonstrated a significantly higher rate of weight gain in the first 4 weeks, although approximately one third of participants discontinued olanzapine early due to side effects [138]. Norris and colleagues [139] completed a retrospective chart review of 22 inpatients treated with olanzapine compared to an untreated age-matched group. The rate of weight gain was not significantly different, however, the treated group had more psychiatric co-morbidities than those not taking olanzapine and experienced side effects of sedation and dyslipidemia [139]. Hillebrand and colleagues [140] also reported on olanzapine use in seven patients (mean age 16.0 years) with AN. Most were taking 5 mg of olanzapine, with one patient receiving 15 mg once daily. The authors found reductions in activity levels in the adolescents taking olanzapine in comparison to 11 adolescents not treated with olanzapine. All patients were receiving either inpatient or day hospital care and there were no significant differences in weight [140].

In terms of case series, Leggero and colleagues [142] reported on 13 young patients (age 9.6 to 16.3 years) treated with a mean dose of 4.13 mg daily of olanzapine. Significant improvements were seen in weight, functioning, eating disorder symptoms and hyperactivity. Similarly, Swenne and Rosling [141] reported on 47 adolescents with AN treated with a mean dose of 5.1 mg daily. A mean weight gain of 9 kg was noted. The patients were treated for a mean of 228 days with olanzapine and were followed for three months following medication discontinuation. Biochemical side effects were closely monitored and were felt to be more related to refeeding processes than to medication [141].

Thirteen case reports (Table 24) have also been published [42, 143–154]. Pisano and colleagues [143] reported on five cases of adolescents with AN treated with 2.5 to 7.5 mg of olanzapine. At 6 month follow-up these patients demonstrated increased oral intake and improved BMI. Dennis, Le Grange, and Bremer [144] used olanzapine at a dose of 5 mg daily in five adolescent females with AN and found an increase in BMI, reduction of body concerns, and improvements in sleep and anxiety surrounding food and weight. Another case series involving four young patients aged 10 to 12 years reported on the use of olanzapine at a dose of 2.5 mg daily to treat AN [145]. These authors reported improvements in compliance and weight gain, as well as decreases in agitation. Mehler et al. [146] reported on five female patients aged 12 to 17 years on a dose range of 5 mg to 12.5 mg daily of olanzapine. They found improvements in body image distortion and rigidity. La Via, Gray, and Kaye [147] described two females with AN who experienced reduction of inner tension and “paranoid ideas” with use of 10 mg daily of olanzapine. Finally, there is a case report using olanzapine 5 mg daily to treat a 17 year old girl with AN and co-morbid pervasive developmental disorder not otherwise specified [150]. These authors reported weight restoration and improvements in eating behavior within 5 months of initiating treatment.

##### Eating disorder not otherwise specified

Olanzapine was used in a case report of a 12 year old female with EDNOS with improvements on the clinical global impressions scale at a dose of 10 mg daily [156] (Table 25).

**Table 25:** Olanzapine for children and adolescents with OSFED/EDNOS

Certainty assessment							Impact	Certainty	Importance
№ of studies	Study design	Risk of bias	Inconsistency	Indirectness	Imprecision	Other considerations			
Global improvement (assessed with: Clinical Global Impressions Scale)									
1	Case Report	very serious ^a^	not serious	serious ^b^	not serious	none	Single case report of 12 year old female with EDNOS. CGI improved with olanzapine 10 mg daily.	⨁◯◯◯ VERY LOW	IMPORTANT

Explanations

^a^single case report, no control

^b^outcome measured does not really answer our clinical question

Bibliography:

Case Report - Bozabali 2002 [156]

##### Avoidant/restrictive food intake disorder

In a recent case series, Spettigue and colleagues [53] described six patients with ARFID and co-morbid anxiety (median age 12.9 years) who were treated with a combination of family therapy plus pharmacotherapy (Table 26). All patients were treated with olanzapine in combination with other medications, making interpretation of the results difficult: three cases were treated with a combination of olanzapine and fluoxetine, one case was treated with olanzapine followed by fluvoxamine, and two cases were treated with a combination of olanzapine, cyproheptadine and fluoxetine. All six cases reached their treatment goal weights.

**Table 26:** Olanzapine for children and adolescents with avoidant/restrictive food intake disorder

Certainty assessment							Impact	Certainty	Importance
№ of studies	Study design	Risk of bias	Inconsistency	Indirectness	Imprecision	Other considerations			
Weight (assessed with: lbs), Anxious/Depressive Symptoms									
2	Case Reports	very serious ^a^	not serious	not serious	not serious	none	*N* = 15 total in two studies. Nine patients aged 9–19 years in this pre- post- study. Rate of weight gain increased significantly with olanzapine treatment from 3.3lbs to 13.1 lbs. All patients were in a residential treatment facility. Another case series of 6 patients indicated all patients gained to their target weight with olanzapine (2.5 to 7.5 mg daily) in combination with SSRIs and family therapy.	⨁◯◯◯ VERY LOW	IMPORTANT
		very serious ^a^	not serious	not serious	not serious	none	The Clinical global impressions scale was used to rate anxious/depressive symptoms for 9 patients pre and post. The rating changed from markedly ill to mildly ill. All patients were in a residential treatment facility.	⨁◯◯◯ VERY LOW	IMPORTANT

Explanations

^a^small sample size, no control group

Bibliography:

Case Reports - Brewerton 2017 [157], Spettigue 2018 [53]

Another recent case series reported beneficial effects from olanzapine in the treatment of patients with ARFID [157]. These authors completed a retrospective chart review and described a significant increase in weight, as well as improvements in anxiety and depressive symptoms in nine patients with ARFID treated with olanzapine. The mean final dose of olanzapine was 2.8 mg daily. All nine patients had comorbid mental health diagnoses including separation anxiety, obsessive-compulsive disorder, posttraumatic stress disorder, generalized anxiety disorder, and social anxiety disorder. Six of the nine also had significant major depressive symptoms.

#### Risperidone

##### Anorexia nervosa

The use of risperidone for AN has been studied in one high quality RCT and four case reports (Table 27). Hagman and colleagues [158] conducted a double-blind placebo-controlled trial of risperidone in adolescents and young adults with AN (age range 12 to 21 years). These authors randomized 40 patients to risperidone or placebo. The mean dose of risperidone was 2.5 mg daily over a mean duration of 9 weeks. There were no differences found between the groups at the end of the study [158]. Personal communication with the primary author indicates that even when the subgroup of patients under age 18 years was examined, no differences were found. These authors concluded that their results do not support the use of risperidone in the weight restoration phase of treatment for young patients with AN [158].

**Table 27:** Risperidone for children and adolescents with anorexia nervosa

Certainty assessment							Impact	Certainty	Importance
№ of studies	Study design	Risk of bias	Inconsistency	Indirectness	Imprecision	Other considerations			
Weight (assessed with: kg), Psychological Symtpoms, Side Effects									
1	randomised trials	not serious	not serious	not serious	not serious	none	There were no significant differences in weight at end of study (risperidone *n* = 18, placebo *n* = 22). Even when just data from those under age 18 (placebo 18, risperidone 12) were analyzed separately, there were no differences. Mean dose 2.5 mg over 9 weeks.	⨁⨁⨁⨁ HIGH	CRITICAL
		not serious	not serious	not serious	not serious	none	There were no significant differences at end of study on any subscale of the EDI (Eating Disorders Inventory).	⨁⨁⨁⨁ HIGH	CRITICAL
		not serious	not serious	not serious	not serious	none	ECG, bloodwork (prolactin, lipids, liver enzymes, glucose) no differences. Patient in the treated group reported fatigue and dizziness.	⨁⨁⨁⨁ HIGH	CRITICAL
Weight (assessed with: kg), Psychological Symptoms									
4	Case Reports	very serious ^a^	not serious	not serious	serious ^a^	none	Weight generally increased pre to post study period by several kg in 4 cases.	⨁◯◯◯ VERY LOW	CRITICAL
		very serious ^a^	not serious	not serious	serious ^a^	none	Psychological symptoms including willingness to eat improved over the study period. Rigidity decreased. (*n* = 4)	⨁◯◯◯ VERY LOW	CRITICAL

Explanations

^a^These are four case reports with no comparison condition

Bibliography:

RCT - Hagman 2011 [158]

Case Reports - Fisman 1996 [159], Kracke 2014 [160], Umehara 2014 [161], Newman-Toker 2000 [162]

Four case reports were found on the use of risperidone in the treatment of AN [159–162]. Weight generally increased in all four cases described, and willingness to eat increased. Of these was a case report of a 12 year old girl with autism and AN who is described as benefitting from treatment with risperidone at a dose of 0.5 mg twice daily [159]. One of these cases describes the use of risperidone long-acting injection [161].

##### Avoidant/restrictive food intake disorder

Pennell and colleagues [163] described two cases of ARFID where significant weight loss occurred with stimulant treatment for Attention Deficit Hyperactivity Disorder (ADHD), resulting in the need for hospitalization. These cases were managed by temporarily stopping the stimulant and adding risperidone to help with appetite and behaviour (Table 28).

**Table 28:** Risperidone for children and adolescents with avoidant/restrictive food intake disorder

Certainty assessment							Impact	Certainty	Importance
№ of studies	Study design	Risk of bias	Inconsistency	Indirectness	Imprecision	Other considerations			
Weight, psychological symptoms									
1	Case Report	very serious ^a^	not serious	not serious	not serious	none	In two cases of ARFID on dose of 1 mg daily of risperidone. Weight gain was observed to target weight over several weeks.	⨁◯◯◯ VERY LOW	IMPORTANT
		very serious ^a^	not serious	not serious	not serious	none	Oppositional behaviour and rigidity around eating improved.	⨁◯◯◯ VERY LOW	IMPORTANT

Explanations

^a^two case reports with no control group

Bibliography:

Case Report - Pennell 2016 [163]

#### Quetiapine

##### Anorexia nervosa

Very few studies could be found on the treatment of AN with quetiapine (Table 29). One case series described quetiapine use in three subjects, aged 11 to 15 years with severe AN (lengthy hospitalization, use of nasogastric tubes, and BMI 12.3 to 13.9) [164]. Two of these patients were treated with quetiapine 100 mg twice daily, and one patient was treated with 250 mg twice daily. Authors reported improvements in body image disturbance, weight phobia, and “paranoid ideas”. Sedation and constipation were noted as side effects.

**Table 29:** Quetiapine for children and adolescents with Anorexia Nervosa

Certainty assessment							Impact	Certainty	Importance
№ of studies	Study design	Risk of bias	Inconsistency	Indirectness	Imprecision	Other considerations			
Weight, fear of weight gain, side effects									
1	Case Report	very serious ^a^	not serious	not serious	not serious	none	Three cases described in which weight increased.	⨁◯◯◯ VERY LOW	CRITICAL
		very serious ^a^	not serious	not serious	not serious	none	Fear of weight gain improved.	⨁◯◯◯ VERY LOW	CRITICAL
		very serious ^a^	not serious	not serious	not serious	none	Side effects - Initial Fatigue, constipation.	⨁◯◯◯ VERY LOW	CRITICAL

Explanations

^a^this a series of three cases with no control group

Bibliography:

Case Report - Mehler-Wex 2008 [164]

#### Aripiprazole

##### Anorexia nervosa

One case control study and two case reports were found on the use of aripiprazole in AN (Table 30). Frank and colleagues completed a retrospective case control study [165] and a case report [166] on the use of aripiprazole in adolescents with AN. The chart review described 22 adolescents with AN taking aripiprazole at a mean dose of 3.59 mg daily compared to an untreated comparison group of 84 adolescents with AN. These authors found a greater increase in BMI in the treated group [165]. The case report described four adolescents who benefitted in terms of weight and improved eating disorder cognitions [166]. One other case report was found on the use of aripiprazole [167]. The adolescent received a dose of 5 mg daily. The authors report an improvement in anxiety and rigidity around eating with aripiprazole.

**Table 30:** Aripiprazole for children and adolescents with Anorexia Nervosa

Certainty assessment							Impact	Certainty	Importance
№ of studies	Study design	Risk of bias	Inconsistency	Indirectness	Imprecision	Other considerations			
Weight									
1	Case Control	serious ^a^	not serious	not serious	not serious	none	Retrospective case-control study with 22 subjects treated with aripiprazole, 84 no treatment. BMI was slightly different between groups 18.8 vs. 17.9 *p* < 0.35.	⨁◯◯◯ VERY LOW	CRITICAL
Weight									
2	Case Reports	very serious ^a,b^	not serious	not serious	not serious	none	5 cases report a benefit in terms of weight gain	⨁◯◯◯ VERY LOW	CRITICAL

Explanations

^a^the study was not randomized

^b^there are five cases reported on in total with no comparison group

Bibliography:

Case Control - Frank 2017 [165]

Case Reports - Frank 2016 [166], Trunko 2011 [167]

##### Avoidant/restrictive food intake disorder

One case report described the beneficial use of fluoxetine (20 mg daily) in combination with aripiprazole (2.5 mg daily) for a 15 year old girl with severe choking phobia [168] (Table 31).

**Table 31:** Aripiprazole for children and adolescents with avoidant/restrictive food intake disorder

Certainty assessment							Impact	Certainty	Importance
№ of studies	Study design	Risk of bias	Inconsistency	Indirectness	Imprecision	Other considerations			
Weight (assessed with: kg), psychological Symptoms									
1	Case Report	very serious ^a^	not serious	not serious	not serious	none	Only one case report. Patient gained 10 kg. Also on fluoxetine.	⨁◯◯◯ VERY LOW	IMPORTANT
		very serious ^a^	not serious	not serious	not serious	none	Psychological symptoms including anxiety and rigidity improved.	⨁◯◯◯ VERY LOW	IMPORTANT

Explanations

^a^one case report, no comparison

Bibliography:

Case Report - Sivri 2018 [168]

#### Antidepressants

**Fig. 4:**
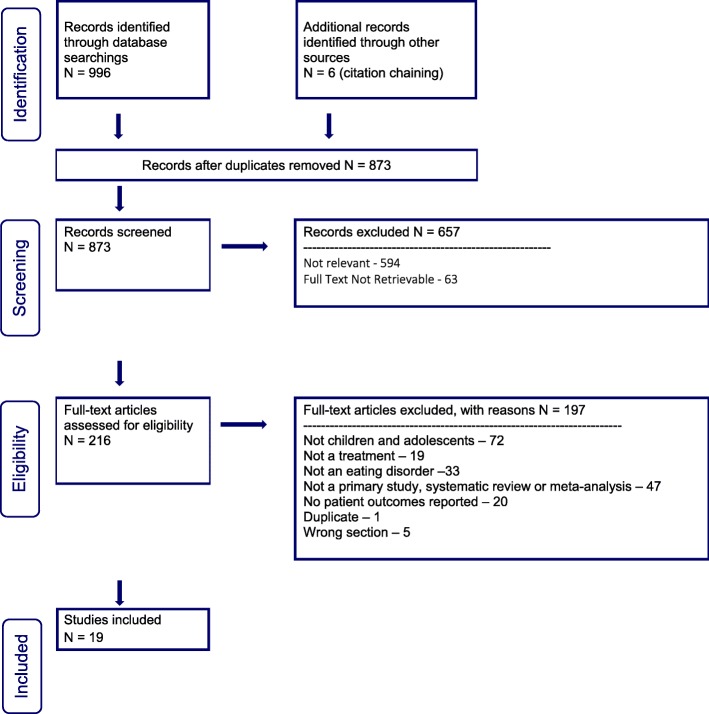
PRISMA flow diagram for antidepressants

Nine hundred and ninety-six abstracts were identified through our database searches along with six additional articles through citation chaining and reference list searching for the antidepressant section of our guideline (see PRISMA flow diagram Fig. 4). Six hundred and fifty-seven citations were excluded on screening. On full text review, 197 articles were excluded, leaving 19 papers for data extraction.

#### Selective serotonin reuptake inhibitors

##### Anorexia nervosa

In terms of Selective Serotonin Reuptake Inhibitors (SSRIs) for AN, one case control study and five case reports were found (Table 32). One retrospective study compared 19 adolescent patients with AN taking SSRIs to 13 patients with AN not treated with SSRIs [169]. These authors found no differences between groups in terms of BMI, eating disorder psychopathology, or depressive and obsessive-compulsive symptoms after evaluating patients on admission, discharge and one-year follow-up. The SSRIs involved in this study included fluoxetine (*n* = 7, mean dose 35 mg daily), fluvoxamine (*n* = 8, mean dose 120 mg daily), and sertraline (*n* = 4, mean dose 100 mg daily).

**Table 32:** SSRIs for children and adolescents with Anorexia Nervosa

Certainty assessment							Impact	Certainty	Importance
№ of studies	Study design	Risk of bias	Inconsistency	Indirectness	Imprecision	Other considerations			
Weight (assessed with: BMI), Core Eating disorder Symptoms (assessed with: ANIS), Depression (assessed with: DIJK)									
1	Case Control	serious ^a^	not serious	not serious	not serious	none	Retrospective chart review - 19 patients on SSRIs (7 fluoxetine 20-60 mg, 8 fluvoxamine 100-150 mg, 4 sertraline 50-150 mg) compared to 13 on no medication. No differences in BMI.	⨁◯◯◯ VERY LOW	CRITICAL
		serious ^a^	not serious	not serious	not serious	none	No differences in core ED pathology.	⨁◯◯◯ VERY LOW	CRITICAL
		serious ^a^	not serious	not serious	not serious	none	No difference in depression scores between treated and untreated groups.	⨁◯◯◯ VERY LOW	CRITICAL
		serious ^a^	not serious	not serious	not serious	none	No difference in obsessive compulsive symptoms between treated and untreated groups.	⨁◯◯◯ VERY LOW	CRITICAL
Weight (assessed with: kg)									
5	Case Reports	very serious ^a,b^	not serious	not serious	not serious	none	Five case reports (3 fluoxetine 20 mg, 2 sertraline 75-100 mg) are described in which patients had a good response to various SSRIs and gained weight.	⨁◯◯◯ VERY LOW	CRITICAL

Explanations

^a^Non randomized study

^b^No control condition

Bibliography:

Case Control - Holtkamp 2005 [169]

Case Report - Frank 2001 [170], Newman Toker 2000 [162], Lyles 1990 [171], Ercan 2003 [151], Gee 1999 [172]

Five adolescent case reports have been published on the use of SSRIs in AN. One of these focused on the use of sertraline in an adolescent with AN and symptoms of purging [170], another on the use of fluoxetine in an adolescent with AN and depressive features [171], and another on the use of fluoxetine for comorbid obsessive compulsive disorder [172]. All of these cases described a benefit in terms of anxiety, mood and weight restoration. Two additional case reports examined SSRIs in combination with antipsychotics [151, 162]. Newman-Toker [162] described two cases of adolescents with AN in which risperidone (1.5 mg daily) was added to antidepressant treatment, with improvements in anxiety and weight gain. Similarly, Ercan and colleagues [151] described a case of a 15 year old female with severe AN treated with olanzapine, fluoxetine, alprazolam, and thioridazine, demonstrating that polypharmacy is sometimes needed for severe symptoms of AN including agitation and fear of weight gain. These authors also reported that once stabilized in terms of agitation, a maintenance dose of 10 mg of olanzapine daily resulted in an increase in BMI, along with a reduction of obsessive-compulsive symptoms, exercising, and eating disorder cognitions [151].

##### Bulimia nervosa

Selective serotonin reuptake inhibitors have been studied in children and youth with BN, although the evidence is scant (Table 33). One open trial of fluoxetine in ten adolescents aged 12 to 18 years [173] reported on 8 weeks of a titrating dose of fluoxetine (maximum 60 mg daily) along with supportive psychotherapy. Frequencies of binge episodes decreased significantly from a mean of 4.1 to zero episodes per week, and weekly purges decreased from 6.4 to 0.4 episodes per week [173]. Seventy percent of patients were rated as improved or much improved on the clinical global impressions-improvement scale. No significant side effects were noted. Whether patients maintained these benefits over the long term is unknown.

**Table 33:** SSRIs for children and adolescents with Bulimia Nervosa

Certainty assessment							Impact	Certainty	Importance
№ of studies	Study design	Risk of bias	Inconsistency	Indirectness	Imprecision	Other considerations			
Binge Frequency (assessed with: average weekly binges), purge frequency, psychological symptoms, depression (BDI)									
1	Case Series	very serious ^a^	not serious	not serious	not serious	none	Ten subjects all female, no control group. 8 week study of fluoxetine 60 mg/day. Binge frequency decreased from 4.1 to 3.8 (*p* < 0.01). Purge frequency decreased from 6.4 to 5.2 (*p* < 0.005).	⨁◯◯◯ VERY LOW	CRITICAL
		very serious ^a^	not serious	not serious	not serious	none	EDI Bulimia Subscale decreased significantly from 10.6 to 4.2 (*P* < 0.01).	⨁◯◯◯ VERY LOW	CRITICAL
		very serious ^a^	not serious	not serious	not serious	none	BDI scores were not significantly different pre and post.	⨁◯◯◯ VERY LOW	CRITICAL
Adverse Effect - Mania									
1	Case Report	very serious ^a,b^	not serious	not serious	not serious	none	Case described of teen with BN treated with fluoxetine 20 mg who developed mania - fluoxetine stopped and valproate started.	⨁◯◯◯ VERY LOW	CRITICAL

Explanations

^a^no control group

Bibliography:

Case Series - Kotler 2003 [173]

Case report - Tor 2008 [174]

One case report describes the use of valproate 200 mg twice daily following onset of mania felt to be related to the use of fluoxetine in an adolescent female with BN. In this report mood stabilized and binge eating and purging symptoms resolved once the fluoxetine had been stopped and valproate was initiated [174].

##### Other specified feeding and eating disorders

Our review identified one case report of a patient with Other Specified Feeding and Eating Disorder (OSFED), atypical AN, whose depressive symptoms were treated with escitalopram with improvement noted [175]. She had lost almost 40 kg over a period of 4 months, but remained within a normal weight range (Table 34). Body image concerns remained.

**Table 34:** SSRIs for children and adolescents with OSFED/EDNOS

Certainty assessment							Impact	Certainty	Importance
№ of studies	Study design	Risk of bias	Inconsistency	Indirectness	Imprecision	Other considerations			
Depressive symptoms (assessed with: clinical impression)									
1	Case Report	very serious ^a^	not serious	not serious	not serious	none	Single case report of adolescent female, initially overweight with depressive symptoms. Treated with escitalopram 10 mg and depressive symptoms improved.	⨁◯◯◯ VERY LOW	IMPORTANT

Explanations

^a^no control group, single case report

Bibliography:

Case Report - Wolter 2009 [175]

##### Avoidant/restrictive food intake disorder

In terms of the ‘post-traumatic’ subtype of ARFID where there has been a choking event followed by refusal to eat and drink, the SSRIs have been described in case reports as being helpful (Table 35). Several SSRIs have been mentioned in case reports including; escitalopram [177] and fluoxetine [120, 178]. Of note, Celik and colleagues reported a case of two 2-year old twins who were treated with fluoxetine 5 mg daily for a severe posttraumatic food avoidance, with good effect [178]. Similarly, a case series of three children with “severe choking phobias” were successfully treated with low-dose SSRIs (sertraline and paroxetine) [176]. Spettigue and colleagues [53] also described the treatment of six children with ARFID treated with combinations of SSRIs and antipsychotics (described above in more detail in the olanzapine section).

**Table 35:** SSRIs for children and adolescents with avoidant/restrictive food intake disorder

Certainty assessment							Impact	Certainty	Importance
№ of studies	Study design	Risk of bias	Inconsistency	Indirectness	Imprecision	Other considerations			
Anxiety (assessed with: clinical impression)									
5	Case Reports	very serious ^a^	not serious	not serious	not serious	none	13 patients (3 male, 10 female) treated with various SSRIs including fluoxetine (8), paroxetine (2), fluvoxamine (1), sertraline (1), escitalopram (1). All cases experienced an improvement in anxiety and improved eating.	⨁◯◯◯ VERY LOW	CRITICAL

Explanations

^a^no control group

Bibliography:

Case Reports - Banerjee 2005 [176], Hosoglu 2018 [177], Spettigue 2018 [53], Celik 2007 [178], Bailly 2003 [120]

#### Other antidepressants - mirtazapine

##### Anorexia nervosa

To date, one case control study as well as two case reports involving the use of mirtazapine in AN have been published (Table 36). Hrdlicka and colleagues [179] examined nine adolescent patients with AN who had been treated with mirtazapine for anxiety or depression compared to nine female controls with AN. The two groups were matched in terms of age and BMI. The mean dose of mirtazapine was 21.7 mg daily. There were no significant differences in terms of weight or BMI at the end of this study [179].

**Table 36:** Mirtazapine for children and adolescents with Anorexia Nervosa

Certainty assessment							Impact	Certainty	Importance
№ of studies	Study design	Risk of bias	Inconsistency	Indirectness	Imprecision	Other considerations			
Weight (assessed with: kg)									
1	Case Control	serious ^a^	not serious	not serious	not serious	none	9 females with AN treated with mirtazapine (mean dose 21.7 mg/day) matched with 9 controls. No significant differences in weight or BMI at the end of 4 weeks of treatment.	⨁◯◯◯ VERY LOW	CRITICAL
Weight (assessed with: kg) Depression (assessed with: clinical impression)									
2	Case Reports	very serious ^a,b^	not serious	not serious	not serious	none	Two case reports (one male, one female) with AN and depression. Both improved in weight.	⨁◯◯◯ VERY LOW	CRITICAL
		very serious ^a,b^	not serious	not serious	not serious	none	One of these case reports mentioned remission of depression in the context of AN with treatment with mirtazapine (30 mg).	⨁◯◯◯ VERY LOW	CRITICAL

Explanations

^a^subjects were not randomized

^b^no control condition

Bibliography:

Case Control - Hrdlicka 2008 [179]

Case Report - Jaafar 2007 [180], Naguy 2018 [181]

In terms of the case reports, the first case report described a 16 year old female hospitalized for AN and depression treated with mirtazapine [180]. These authors found positive results in terms of weight restoration and mood improvement, and suggested further study of the medication was needed. More recently, Naguy and Al-Mutairi [181] described the case of a 16 year old boy hospitalized for severe AN who responded well to mirtazapine 30 mg/day in terms of weight restoration.

##### Avoidant/restrictive food intake disorder

For ARFID, mirtazapine has also been used to good effect, although the evidence is limited to one case series and one case report (Table 37). The case series described 14 cases with the rate of weight gain reported pre and post initiation of mirtazapine (average dose 25.5 mg daily) [182]. Rate of weight gain was significantly greater after the initiation of the medication. An additional case report described the treatment of a 10 year old girl with ARFID and Obsessive-Compulsive Disorder (OCD). Anxiety and eating improved with 15 mg daily [183].

**Table 37:** Mirtazapine for children and adolescents with avoidant/restrictive food intake disorder

Certainty assessment							Impact	Certainty	Importance
№ of studies	Study design	Risk of bias	Inconsistency	Indirectness	Imprecision	Other considerations			
Mealtime Anxiety (assessed with: clinical impression)									
1	Case Series	very serious ^a^	not serious	not serious	not serious	none	Retrospective chart review of 14 cases pre and post documentation of rate of weight gain pre and post mirtazapine. Rate of gain significantly greater after mirtazapine (mean dose 25.5 mg).	⨁◯◯◯ VERY LOW	CRITICAL
Anxiety									
1	Case Report	very serious ^a^	not serious	not serious	not serious	none	Single case report of 10 yo girl with ARFID and OCD treated with 15 mg/day of mirtazapine. Anxiety improved and she began to eat solid food within 1–2 weeks.	⨁◯◯◯ VERY LOW	CRITICAL

Explanations

^a^no control condition

Bibliography:

Case series – Gray 2018 [182]

Case Report - Tanidir 2015 [183]

##### Lack of evidence

No studies could be found on the use of Selective Norepinephrine Reuptake Inhibitors (SNRIs) for this population. The same was true for Mood Stabilizers.

### Level of care

**Fig. 5:**
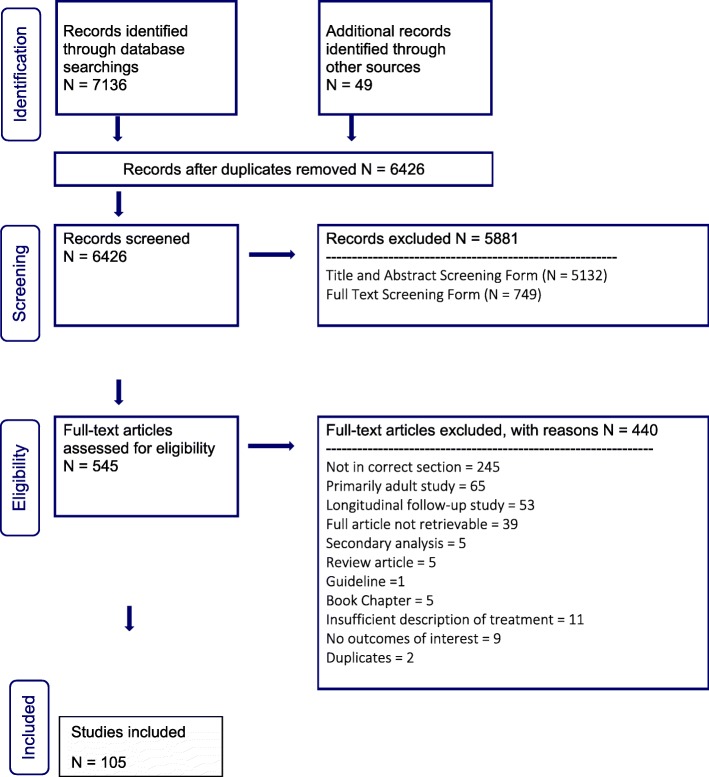
PRISMA flow diagram for inpatient, day hospital and residential treatment

The database search initially provided 7136 citations, as reported in the PRISMA flow diagram (Fig. 5). An additional 49 citations were added through review of references, and forward citation chaining. After removing the duplicates, 6426 records remained, of which 5881 were eliminated on screening given that they did not meet the inclusion criteria. Of the 545 full text articles assessed for eligibility, 440 full text articles were excluded because they were longitudinal follow-up studies, primarily adult studies, review or secondary analysis papers, book chapters or guidelines, did not provide sufficient description of the treatment provided, did not focus on inpatient treatment or otherwise did not meet the inclusion criteria. Ultimately, 105 studies were selected for inclusion in the level of care section of this guideline – 70 within the inpatient subsection, 29 within the day hospital subsection, and six within the residential subsection.

### Inpatient

#### Multimodal treatment

##### Anorexia nervosa

Twenty-one observational studies, none of which included control or comparison groups, have been published for a combined total of 1347 patients (Table 38) [184–191, 193–196, 198, 199, 201–207]. Various measures of change in weight were used across these studies including BMI, absolute weight in kg, percent Treatment Goal Weight (%TGW), weight gain per week and percent of patients attaining a predetermined discharge weight prior to discharge. Mean change in weight was positive in all studies. Mean length of stay ranged from 20.10 to 328.5 days.

**Table 38:** Multimodal inpatient treatment for anorexia nervosa and/or low weight eating disorders

Certainty assessment							Impact	Certainty	Importance
№ of studies	Study design	Risk of bias	Inconsistency	Indirectness	Imprecision	Other considerations			
Weight (assessed with: Change in Weight Measures from Admission to Discharge), ED Symptoms (EDE-Q, EDI, EAT), motivational stage of change, laxative use, binge eating									
20	Case Series	very serious ^a^	serious ^b^	not serious	serious ^c^	none	Twenty studies examined change in weight during inpatient treatment for total 1346 patients. Various measures of change in weight used across studies including BMI, absolute weight in KG, %TGW, weight gain per week and % of patients attaining predetermined D/C weight prior to d/c. Seventeen (*N* = 1319) used BMI as measure of weight. Mean BMI at admission varied from 13.2 to 16.3 between studies. Mean BMI at d/c varied from 16.3 to 19.49. Change in BMI from admission to d/c varied from 1.4 to 4.1. One study (*n* = 40) reported on mean BMI% change which rose from BMI 8.98 (+/−2.07) to 21.25 (+/− 3.13). Six studies (*n* = 134) reported mean absolute weight gain during admission which varied from 5.4 to 10.1 kg. Three studies (*N* = 151) reported mean %TGW change admission to discharge of 10.3 and 10.5%. One study (*n* = 40) only reported weight outcomes as rate of weight gain per week which was 1.86 kg/wk. with a mean LOS of 20.63 days (SD 13.03). Finally 2 studies reported on the % of patients attaining a pre-determined adequate weight as inpatients with 1 study reporting 76.1% (*n* = 196) reaching a mean BMI of > 17.63 and 1 study reporting 79.6% (*n* = 108) attaining > 90%TGW at time of d/c. LOS varied considerably which is likely related to difference in weight change as an inpatient. Mean LOS ranged from 20.10 to 328.5 days between studies. One study noted that longer LOS, lower age at admission and no previous inpatient treatment was associated with greater improvement in BMI.	⨁◯◯◯ VERY LOW	CRITICAL
		very serious ^d^	serious ^e^	not serious	serious ^c^	all plausible residual confounding would reduce the demonstrated effect	Three studies - Two self-report measures of symptoms were used (EDI-3 and EDE-Q), change reported from admission to discharge. Treatment provided was multimodal. Three studies (total *n* = 88) reported on EDE-Q. Change in EDE-Q was found to be significant in one of these studies (*n* = 44, *p* < 0.05) - this difference was attributed to the restraint and eating concerns subscales. In the other 2 studies there was no difference in EDE scores from admission to discharge. LOS for these studies was a mean of 203 and 115 days. BMI at discharge was higher in the study which found significant change in EDE-Q (ie BMI 19.49 vs 18.5 and BMI% 21.25 at discharge).	⨁◯◯◯ VERY LOW	CRITICAL
		very serious ^d^	serious ^e^	not serious	serious ^c^	all plausible residual confounding would reduce the demonstrated effect	All three studies (total *n* = 126) reported EAT scores at admission and discharge. Two studies used the EAT-26 and 1 study used the EAT-40. Treatment was multimodal and varied between studies. The difference in EAT score was noted to be statistically different in 2 studies (*p* < 0.001) and the third study reported a difference of 19 on the EAT-26 pre-post. LOS varied between studies (29.8 days, 91 days and not reported). Mean BMIs at discharge in these 3 studies were 19.2. 18.4 and 16.3.	⨁◯◯◯ VERY LOW	CRITICAL
		very serious ^f^	serious ^e^	not serious	serious ^g^	all plausible residual confounding would reduce the demonstrated effect	One study - Number of patients reporting laxative use, binge/purge, exercise symptoms, even at admission were exceedingly small (ie laxatives 0, bingeing 3, exercise 5). Overall study small (total n = 11 at admission and 7 at discharge). No statistical change noted in any of these outcomes.	⨁◯◯◯ VERY LOW	IMPORTANT
		very serious ^d^	not serious	not serious	serious ^c^	all plausible residual confounding would reduce the demonstrated effect	One study with *n* = 49 patients and mean LOS 30 days. Change in mean ANSOCQ was statistically significant, however both admission and d/c scores fall into “preparation” phase of motivation and confidence intervals wide (ie admit score 53.6, SD 19.7 and d/c score 62.9, SD 24.5). During the course of the study BMI rose from 15.5 to 18.4.	⨁◯◯◯ VERY LOW	IMPORTANT
		very serious ^d^	serious ^e^	not serious	serious ^c^	strong association all plausible residual confounding would reduce the demonstrated effect	Three studies (*n* = 353), mean LOS 115 days, 33.61 and 81.9 days respectively, reported on EDI-2 outcomes. One study (LOS 115 days) found no significant change in total or subscales of EDI-2 from admission to discharge. One study (*n* = 71 and LOS 33.61 days) found statistically significant improvement on Drive for Thinness (13.19 +/− 6.86 at admission and 11.23 +/− 6.52 at discharge, *p* < 0.05) and Bulimia (1.50 +/− 2.15 at admission and 0.66 +/− 1.08 at discharge, *p* < 0.05), but no change in Body Dissatisfaction. The final study (*n* = 238) found statistically significant improvements in global (ES 0.8) and all subscales of the EDI-2. The largest effect size was found for Drive for Thinness (ES = 1.1) and the lowest for “Maturity Fears” (ES = 0.3).	⨁◯◯◯ VERY LOW	CRITICAL
Weight									
1	Case Study	very serious ^d^	serious ^e^	not serious	serious ^c^	strong association all plausible residual confounding would reduce the demonstrated effect	One case report describing a 17.1 kg wt gain	⨁◯◯◯ VERY LOW	CRITICAL

Explanations

^a^Observational studies with no comparison group

^b^Multimodal treatment not well described/defined

^c^Confidence interval wide and cross over threshold for change

^d^Self-report measures and no control/comparison group

^e^Differing inclusion/exclusion criteria and treatments provided

^f^Unclear how these symptoms were measured and study took place over two sites which may have resulted in variation

^g^Number of patients in study small and numbers reporting these particular symptoms even smaller

Case Series – Anis 2016 [184], Ayton 2009 [185], Castro-Fornieles 2007 [186], Fennig 2017 [187], Goddard 2013 [188], Heinberg 2003 [189], Kalisvaart 2007 [190], Leon 1985 [191], Lievers 2009 [192], Mekori 2017 [193], Morris 2015 [194], Nova 2007 [195], Roux 2016 [196], Schlegl 2016 [197], Shugar 1995 [198], Tasaka 2017 [199], Treat 2008 [200], Vall 2017 [201], Bourion-Bedes 2013 [202], Rothschild-Yakar 2013 [203]

Case Reports – Toms 1972 [204]

While all of the observational studies of multimodal inpatient treatment reported on change in weight, fewer reported on change in eating disorder symptoms. Three studies (total *n* = 88) reported on Eating Disorders Examination-Questionnaire (EDE-Q) and one of the three studies reported significant change (*n* = 44, *p* < 0.05) [187, 201, 207]. This pre-post difference was attributed predominantly to the restraint and eating concerns subscales. Mean length of stay for these studies was between 203 and 115 days. Three studies (total *n* = 126) reported improvements in Eating Attitudes Test (EAT) scores at admission and discharge [186, 198, 203]. Length of stay varied between these three studies (29.8 days, 91 days and not reported). One study (total *n* = 44), with a mean LOS of 115 days reported on Eating Disorder Inventory (EDI) scores at admission and discharge [187]. This study found no significant change in total or subscales of the EDI. One study reported on frequency of binge, laxative and exercise symptoms, however the total number of patients reporting these symptoms at admission was small (i.e. laxatives 0, bingeing 3, exercise 5) [188]. Overall the study population was small (total *n* = 11 at admission and 7 at discharge). No statistical change was noted in any of these outcomes.

Although not a focus of our guideline, one study measured motivation for change using the Anorexia Nervosa Stage of Change Questionnaire (ANSOCQ) at admission and then again at discharge [186]. The study included 49 patients whose mean length of stay was 30 days. Change in mean ANSOCQ score was noted to be statistically significant, however both admission and discharge scores fell into the “preparation” phase of motivation and the confidence intervals were wide.

##### Mixed diagnoses

Two studies of multimodal inpatient treatment were found which reported on weight gain during inpatient treatment for patients with mixed eating disorder diagnoses (Table 39). One study differentiated between patients with AN restricting type versus those with AN binge-purge type or BN [203], and the other differentiated between those with AN restricting type or AN binge-purge type versus those with BN or Eating Disorder Not Otherwise Specified binge-purge type (EDNOS-B/P) [193]. Multimodal treatment was provided in both studies but varied between studies. Total number of patients studied was 150 across the two studies. In both cases there was a significantly greater increase in BMI for the group containing AN restricting type patients (total *n* = 94). In both cases this group started with a much lower BMI. Length of stay in these studies was approximately 6–7 months.

**Table 39:** Multimodal inpatient treatment for children and adolescents with eating disorders

Certainty assessment							Impact	Certainty	Importance
№ of studies	Study design	Risk of bias	Inconsistency	Indirectness	Imprecision	Other considerations			
Change in Weight (assessed with: Change in BMI from Admit to D/C)									
2	Case Series	very serious ^a^	serious ^b^	not serious	not serious	none	One study differentiated between patients with AN-R vs those with AN-B/P or BN and the other differentiated between those with AN-R or AN-B/P and those with BN or EDNOS-B/P. Multimodal treatment was provided in both studies, but varied between studies. Total *n* = 150 across the two studies. In both studies there was a significantly greater increase in BMI for the group containing AN-R patients (total *n* = 94). In both studies this group started with a much lower BMI (ie 14.94 and 15.78) and d/c BMI was 19.24 and 19.79. In the group containing only BN and EDNOS-B/P (*n* = 27) there was no change in BMI during admission. LOS in these studies was 6.25 +/− 2.28 months and 6.8 +/− 3 months.	⨁◯◯◯ VERY LOW	CRITICAL
		very serious ^a,c^	not serious	not serious	serious ^d^	all plausible residual confounding would reduce the demonstrated effect	Study compared EAT-26 at admission to discharge in group of patients with AN-R (*n* = 33) vs AN-B/P or BN (*n* = 29). Overall there was a statistically significant improvement in EAT-26 over the course of the admission (*p* < 0.001). In AN-R groups EAT-26 score decreased from 41.8 (SD 18.56) to 32.17 (SD 22.2) and in AN-B/P or BN group EAT-26 score decreased from 46.67 (SD 15) to 28.83 (SD 14.74). There was no significant difference in change on EAT-26 by diagnosis. LOS was 6.25 +/− 2.28 months.	⨁◯◯◯ VERY LOW	IMPORTANT

Explanations

^a^Observational Study with no control/comparison

^b^Differing inclusion/exclusion criteria and treatments provided

^c^Self-report scale

^d^Wide confidence intervals which cross over threshold of change

Bibliography:

Case Series - Rothschild-Yakar 2013 [203], Mekori 2017 [193]

One of these studies compared symptom change using the EAT at admission to discharge in the group of patients with AN restricting type (*n* = 33) versus AN binge-purge type or BN (*n* = 29 ) [203]. Overall there was a statistically significant improvement in EAT scores over the course of the admission. There was no significant difference in change on EAT by diagnosis.

##### Avoidant/restrictive food intake disorder

Four articles were found which reported on the inpatient treatment of a total of thirteen children treated using either a family-based or cognitive behaviour therapy approach [53, 208–210] (Table 40). Length of stay for these studies varied from 16 days to 60 days. In two of these studies weight gain was reported as an outcome and all patients gained weight [53, 208]. One of these studies reported on caloric intake in kcal/day which rose for all three patients [208]. The third study reported on two cases of females ages 17 and 13 years who were “severely underweight” due to the onset of vomiting and food refusal [209]. After admission, nasojejunum (NJ) tubes were placed to initiate refeeding when oral feeding was not tolerated. The authors reported that the use of an individualized behaviour plan for each patient providing reinforcements for eating was the critical factor which helped these patients to tolerate oral intake without vomiting and allowed for the removal of the NJ tubes.

**Table 40:** Inpatient Treatment for ARFID

Certainty assessment							Impact	Certainty	Importance
№ of studies	Study design	Risk of bias	Inconsistency	Indirectness	Imprecision	Other considerations			
Weight Change (assessed with: Pre-post weight in KG), caloric intake, ability to tolerate oral intake									
4	Case Reports	very serious ^a,b^	not serious	not serious	not serious	none	Two articles reporting on case studies of 3 boys with ARFID ages 6–8 yrs. treated in an inpatient CBT-based treatment program and 2 studies reporting on a total of 5 children ages 9–13 yrs. treated in a family-based inpatient setting. LOS varied from 16 days to 112 days. All patients gained weight. For studies reporting on absolute weight gain the cases gained 2.7 kg in 60 days, 1.2 kg in 16 days and 0.3 kg in 19 days). At discharge these patients were at 97, 104 and 96%TGW. For the study reporting on change in %TGW, patients weight improved from 83 to 100%TGW (in 38 d), 75.8 to 100%TGW (in 2 months) and 72 to 88%TGW (in 2 months) and 69 to 86.8%TGW (LOS unclear).	⨁◯◯◯ VERY LOW	CRITICAL
		very serious ^a^	not serious	not serious	not serious	none	One study reporting on 3 case studies on boys with ARFID ages 6–8 years treated in an inpatient CBT-based program. LOS varied from 16 to 60 days. Caloric intake in kcal/day rose for all 3 patients (from 1557 to 2208, 740 to 1300, and 1200 to 1500).	⨁◯◯◯ VERY LOW	CRITICAL
		very serious ^b^	not serious	not serious	serious ^c^	none	One study reporting on 2 cases of females ages 17 and 13 yrs. and one study describing two 9 yo girls. In the adolescent case reports both patients were severely underweight and due to the onset of vomiting and food refusal after admission NJ tubes were placed. Authors report that the use of an individualized behaviour plan for each patient providing reinforcements for eating was the critical factor in helping patients to tolerate oral intake without vomiting. The case reports involving the 9 yo girls, in both cases the patients were exclusively NGT fed due to a refusal of all oral nutrition, but with the addition of family therapy and mobilization from hospital the patients were able to resume eating orally.	⨁◯◯◯ VERY LOW	IMPORTANT

Explanations

^a^Observational study, no comparison/control

^b^Case studies only, likely biased reporting on patients with successful outcomes

^c^Results descriptive only, no quantitative outcomes re frequency of amount of food tolerated

Bibliography:

Case Reports - Pitt 2018 [209], Singer 1992 [208], Spettigue 2018 [53], Rhodes 2009 [210]

#### Family-based inpatient care

##### Anorexia nervosa

There were three studies found examining inpatient treatment utilizing a family-based approach, one of which included 37 patients [211], and the other two studies which included one patient each (i.e. case reports) [39, 63] (Table 41). Length of stay in hospital was a mean of 20.6 weeks (SD = 13.6, range 3–58) in the first study [211] and 10 days in one case report [39] and unclear in the second case report [63]. Mean weight gain was reported as 7.5 kg in the case series [211], a change in BMI from 16.32 to 17.5 in one case report [39], and a change in BMI of 15.4 to 19.5 in the other case report [63]. In the case report by Goldfield and Boachie [63], the family received eight sessions of family-based informed therapy via telepsychiatry as one parent and siblings were not local.

**Table 41:** Family-based inpatient treatment for children and adolescents with anorexia nervosa

Certainty assessment							Impact	Certainty	Importance
№ of studies	Study design	Risk of bias	Inconsistency	Indirectness	Imprecision	Other considerations			
Change in weight (assessed with: Absolute weight gain during admission in kg)									
1	Case Series	very serious^a^	not serious	not serious	not serious	none	One case series including 37 patients. LOS in hospital was a mean of 20.6 weeks (SD = 13.6, range 3–58) in this study. Weight gain was reported as 7.5 kg (SD 4.4, range − 1.1 to 14.8 kg)	⨁◯◯◯ VERY LOW	CRITICAL
Weight									
2	Case Reports	very serious ^a^	not serious	not serious	not serious	none	Two case reports – LOS in hospital was 10 days in one case report and unclear in the second case report. A change in BMI from 16.32 to 17.5 (ie 82%TGW to 85.8%TGW) in one case report, and a change in BMI of 15.4 to 19.5 in the second case report. In the second case report the family received 8 sessions of family-based informed therapy via telepsychiatry as one parent and siblings were not local.	⨁◯◯◯ VERY LOW	CRITICAL

Explanations

^a^Observational study with no comparison or control

Bibliography:

Case Series - Halvorsen 2018 [211]

Case Reports - Goldfield 2003 [63], Matthews 2016 [39]

#### CBT-based inpatient care

##### Anorexia nervosa

Three studies reported on inpatient treatment utilizing a CBT framework [197, 212, 213] (Table 42). These studies included two case series without a control group [197, 213] and one case study [212], for a total of 296 patients. Mean length of stay in these studies varied from 6 days to 90 days. In all studies patients gained weight in hospital.

**Table 42:** CBT-based inpatient treatment for children and adolescents with anorexia nervosa

Certainty assessment							Impact	Certainty	Importance
№ of studies	Study design	Risk of bias	Inconsistency	Indirectness	Imprecision	Other considerations			
Weight Change (assessed with: Pre-post weight measures), EDI-2 Scores pre and post									
2	Case Series	very serious ^a^	not serious	not serious	serious ^b^	none	Two studies - Total 295 patients. In all studies patients gained weight in hospital. Weight change reported differently across studies. One study reported BMI pre/post with BMI increasing from 14.83 (+/− 1.22) at admission to 17.34 (+/− 1.37) at discharge signifying an ES of 2.1. One study reported change in BMI % which rose from mean of 1.46 (+/− 2.41) at admission to 9.44 (+/− 6.68) at discharge.	⨁◯◯◯ VERY LOW	CRITICAL
		very serious ^a^	not serious	not serious	serious ^b^	all plausible residual confounding would reduce the demonstrated effect	One study which included 238 patients, mean LOS 81.9 (+/− 31.9) days. Global score and all subscales of the EDI-2 showed significant improvements. The ES of the Global score was 0.8. For subscales the highest ES was found for Drive for Thinness with an ES of 1.1, and the lowest ES was for Maturity Fears with an ES of 0.3. Forty-five % showed “clinically significant” changes in EDI-2 Global Scores, 23.6% showed “reliable” changes, 28% of patients remained unchanged and 3.7% deteriorated.	⨁◯◯◯ VERY LOW	IMPORTANT
Weight									
1	Case Report	very serious ^a^	not serious	not serious	serious ^b^	none	In the case study weight increased 1.1 kg in 6 days.	⨁◯◯◯ VERY LOW	CRITICAL

Explanations

^a^Observational study, no comparison/control

^b^Confidence intervals wide in some studies and overlapping with any true effect

Bibliography:

Case Series - Salbach-Andrae 2009 [213], Schlegl 2016 [197]

Case Report - Paul 2013 [212]

One of these studies also reported on symptom change and included 238 patients [197]. Global EDI score and all subscales showed significant improvements. Forty-five percent showed “clinically significant” changes in EDI Global Scores, 23.6% showed “reliable” changes, 28% of patients remained “unchanged” and 3.7% “deteriorated”.

#### Behaviour therapy based inpatient care

##### Anorexia nervosa

Fifteen studies reported on inpatient treatment utilizing a behaviour therapy approach (4 case series and 11 case reports ) [214–228] (Table 43). These studies included a total of 219 patients. Length of stay in these studies ranged from 13 days to 6.25 weeks [215, 217, 218]. In all studies patients gained weight.

**Table 43:** Behaviour therapy based inpatient treatment for children and adolescents with anorexia nervosa

Certainty assessment							Impact	Certainty	Importance
№ of studies	Study design	Risk of bias	Inconsistency	Indirectness	Imprecision	Other considerations			
Change in Weight (assessed with: Pre-post measures of weight), Change in EAT scores, EDI Scores									
4	Case Series	very serious ^a^	not serious	not serious	serious ^b^	none	Four Case series utilizing a behaviour therapy approach. Total 198 patients. Various approaches to reporting change in weight. One study reported absolute weight change of 1.89 kg (+/− 1.41) over a mean of 23 days in hospital; one study reported a rise from a mean of 65.9%TGW to 87.4%TGW over 11 weeks. One study reported that patients admitted at > 75%TGW all reached 100%of their TGW by discharge, 91% of those admitted at < 75%TGW not requiring NGT feeds reached their TGW by discharge and only 62% of patients admitted at < 75%TGW and requiring NGT feeds reached 100% of their TGW at discharge. This study also noted that those admitted at > 75%TGW had a mean LOS of 20.8 d, those < 75%TGW at admission had a mean LOS of 18.4d and those < 75%TGW and NGT fed had a mean LOS of 32.7d. The final case series reported weight gain under 2 types of behaviour contracts, varying only with regards to the expected rate of weight gain (ie 0.36 kg/q4d vs 0.55 kg/q4d). Those treated under the contract requiring greater weight restoration gained weight at a faster rate (0.09 kg/d, range 0.04–0.4 kg/d vs 0.17 kg/d, range 0.01–0.64 kg/d), thereby attaining a greater weight gain overall during admission (LOS 28 days). Most case series reported weight gain observed while patients were adhering to a behaviour contract. LOS in these studies ranged from 13 days to 6.25 weeks. In all cases patients gained weight (ranging from 0.17 to 0.63 kg/day.	⨁◯◯◯ VERY LOW	CRITICAL
		very serious ^a^	not serious	not serious	serious ^c^	all plausible residual confounding would reduce the demonstrated effect	One study measured EAT scores in 24 patients at admission and discharge (mean LOS 11 weeks) and reported a change from total mean EAT of 37.1 at admission to 12.7 at discharge (*p* = 0.0001).	⨁◯◯◯ VERY LOW	IMPORTANT
		very serious ^a^	not serious	not serious	serious ^c^	all plausible residual confounding would reduce the demonstrated effect	One study of 24 patients, mean LOS 11 weeks. Reported a change in EDI score “Drive for Thinness” score of 8.0 at admission to 1.9 at discharge (*p* = 0.02). Other subscales and total EDI score not reported.	⨁◯◯◯ VERY LOW	IMPORTANT
Change in Weight, Change in EAT score, change in intake, change in rate of purging									
11	Case reports	very serious ^a^	not serious	not serious	serious ^b^	none	Case reports all described patients gaining weight in hospital ranging from 0.45 kg/wk. to 4.0 kg/wk. Two case reports did not note the LOS andstated that the patients gained 10 kg total and change in BMI from 13.5 to 16.5 during their admissions.	⨁◯◯◯ VERY LOW	CRITICAL
		very serious ^a^	not serious	not serious	serious ^c^	all plausible residual confounding would reduce the demonstrated effect	One case report describing that EAT scores remained high for the first 7 weeks of treatment and weight restoration (4.5 kg over first 7 weeks) and then dropped (from total score of 60 to 10) over the last 3 weeks of 10 week admission.	⨁◯◯◯ VERY LOW	IMPORTANT
		very serious ^a^	not serious	not serious	not serious	strong association	Two case reports describing change in intake measured by kcal/day from admission to discharge. Only one study reported LOS of 39 days. Kcal/day increased from 1600 kcal/d at admission to 3900 kcal/d at discharge in this study. The other study did not report on LOS, but stated that intake increased from 850 kcal/d at admission to 1700 kcal/d at discharge.	⨁◯◯◯ VERY LOW	CRITICAL
		very serious ^a^	not serious	not serious	not serious	none	1 case report describing a decrease in purging after meals from 48% of meals/week to 0% of meals per week. LOS not noted.	⨁◯◯◯ VERY LOW	IMPORTANT

Explanations

^a^Observational studies with no comparison group or control

^b^Wide confidence intervals in some studies, overlapping with any true effect

^c^Confidence intervals not noted

Bibliography:

Case series - Collins 1983 [222], Solanto 1994 [227], Steinhausen 1985 [224], Nygaard 1990 [226]

Case reports - Alessi 1989 [225], Blanchet-Collet 2016 [228], Blinder 1970 [215], Boey 1985 [223], Cinciripini 1983 [221], Clark 1981 [220], Garfinkel 1973 [216], Halmi 1975 [217], Leitenberg 1968 [214], Pertschuk 1978 [218], Poole 1978 [219]

Two of the case reports described change in intake as measured by kcal/day from admission to discharge. Only one of these studies reported the length of stay, which was 39 days. Calorie intake increased from 1600 kcal/d at admission to 3900 kcal/d at discharge in this study [214]. The other study did not report the length of stay, but stated that intake increased from 850 kcal/d at admission to 1700 kcal/d at discharge [221].

Several studies reported on symptom change during admission to hospital. One case report described a decrease in purging after meals from 48% of meals/week to 0% of meals per week, although the length of stay for this patient was not noted [221]. Two studies reported on EAT scores over the course of inpatient treatment. One was a case report describing that EAT scores remained high for the first 7 weeks of treatment and then dropped (from total score of 60 to 10) over the last 3 weeks of a 10-week admission [225]. The other study measured EAT scores in 24 patients at admission and discharge (mean length of stay 11 weeks) and reported a change from total mean EAT of 37.1 at admission to 12.7 at discharge (*p* = 0.0001) [224].

##### Bulimia nervosa

Only one case series of 24 patients was found that examined inpatient treatment specifically for BN, and the treatment provided was based on behaviour therapy [229] (Table 44). The only eating disorder related outcome that was reported was weight. The mean LOS was 9.9 wks. (+/− 3.5 wks.). Weight decreased slightly over admission from a mean BMI of 20.6 to 20.5.

**Table 44:** Behaviour therapy based inpatient treatment for children and adolescents with bulimia nervosa

Certainty assessment							Impact	Certainty	Importance
№ of studies	Study design	Risk of bias	Inconsistency	Indirectness	Imprecision	Other considerations			
Change in weight (assessed with: Pre/post BMI)									
1	Case Series	very serious ^a^	not serious	not serious	not serious	none	One case series, including 24 patients. Mean LOS was 9.9 wks (+/−3.5 wks). Weight decreased slightly over admission from mean BMI of 20.6 +/− 4.3 to 20.5 +/− 2.7.	⨁◯◯◯ VERY LOW	IMPORTANT

Explanations

^a^Observational study with no comparison/control

Bibliography:

Case Series - Wockel 2009 [229]

#### Psychodynamic based inpatient care

##### Anorexia nervosa

Only two reports of a total of six patients being treated as inpatients using a psychodynamic based approach were found [230, 231] (Table 45). The length of stay for these patients varied between 1.5 months and 5 months. Patients were reported to have gained between 1.3 kg/month to 6 kg/month while admitted.

**Table 45:** Psychodynamic based inpatient treatment for children and adolescents with anorexia nervosa

Certainty assessment							Impact	Certainty	Importance
№ of studies	Study design	Risk of bias	Inconsistency	Indirectness	Imprecision	Other considerations			
Change in weight (assessed with: Pre/post measures of weight)									
2	Case Reports	very serious ^a^	serious ^b^	not serious	not serious	none	Two reports of 6 patients total. LOS varied between 1.5 months and 5 months. Patients were reported to have gained between 1.3 kg/month to 6 kg/month while admitted.	⨁◯◯◯ VERY LOW	CRITICAL

Explanations

^a^Case reports only, no comparison/control

^b^Large variation in results, likely due to individual factors of patients described in studies

Bibliography:

Case Reports - Kronenberg 1994 [231], Groen 1966 [230]

#### Admission to pediatric unit

##### Mixed diagnoses

Four studies including a total of 200 patients, examined the effect of admission to a pediatric unit in terms of weight change in hospital [232–235] (Table 46). These studies did not include comparator groups and included patients with AN, BN and EDNOS. Mean length of stay varied between studies from 31 days to 85 days. In all studies weight improved.

**Table 46:** Inpatient admission on pediatric unit for children and adolescents with eating disorders

Certainty assessment							Impact	Certainty	Importance
№ of studies	Study design	Risk of bias	Inconsistency	Indirectness	Imprecision	Other considerations			
Weight Change (assessed with: Pre-post weight measures)									
3	Case Series	very serious ^a^	not serious	not serious	not serious	none	Three case series including a total of 195 patients. Mean LOS varied between studies from 31 days to 85 days. Two studies reported change in weight using %TGW. In both studies weight rose during admission from mean %TGW of 68% (+/− 5.5) to 99% (+/− 7.7); mean %TGW 75.8% (+/− 2.3) to 85.4% (+/− 1.7) and 73.7% (+/− 2.5) to 86.4% (+/− 3.0 kg) (note: results reported in two groups in second study based on whether the patients were followed after discharge).. The final study including 102 children aged 8–12 yrs. with diagnoses of restrictive ED (93.1%) or bulimia (7.1%). At admission the mean weight was 32.3 kg (SD 7.7) and at discharge mean weight was 35.4 kg (SD 8.9).	⨁◯◯◯ VERY LOW	CRITICAL
Weight Change (assessed with: Pre-post weight measures)									
1	Case Report	very serious ^a^	not serious	not serious	not serious	none	In one case report (*n* = 6) study change in weight was reported in kg and rose a mean of 8.8 kg from admission to discharge (*n* = 5)	⨁◯◯◯ VERY LOW	CRITICAL

Explanations

^a^Observational study with no comparison/control

Bibliography:

Case Series - Lock 2003 [234], Jenkins 1987 [233], Meilleur 2012 [235]

Case Report - Maxmen 1974 [232]

### Inpatient adjunctive treatments

#### Adjunctive multi-family/parent group therapy

##### Mixed diagnoses

One study with total 112 patients with various eating disorder diagnoses reported on symptom change as measured by the EDI during admission to a multimodal inpatient eating disorders unit in two groups of patients; those who received adjunctive multi-family group therapy (MFT, *n* = 62) and those who received adjunctive multi-parent group therapy (MPT, *n* = 50) [236] (Table 47). Both MPT and MFT interventions “promoted an autonomy-supportive parental attitude and the adolescents’ autonomy and self-determination.” Parents were encouraged to “create the conditions supporting their daughters’ autonomy in establishing healthy eating at home to indirectly increase their daughters’ motivation”. Affected children were only included in the MFT group. Group format was one introductory 3-h session followed by five 2-h sessions every 2 weeks. Measures were taken pre/post of the intervention. Patients were not randomized, but rather were allocated to MFT versus MPT depending on the time of admission. Results reported a main effect of time on drive for thinness (*p* < 0.001) and body dissatisfaction (*p* < 0.001) as measured by EDI. Both scales improved independent of type of intervention. A separate case series of 32 inpatient adolescents (29 with AN, 3 with BN) described improvements in EDI score pre-post delivery of Family-Oriented Group Therapy [237].

**Table 47:** Multi-family therapy during inpatient treatment versus multi-parent therapy during inpatient treatment for children and adolescents with eating disorders

Certainty assessment							Impact	Certainty	Importance
№ of studies	Study design	Risk of bias	Inconsistency	Indirectness	Imprecision	Other considerations			
Change in eating disorder symptomatology (assessed with: Pre/post EDI-2)									
1	Case Control	serious ^a^	not serious	not serious	not serious	none	One study with total 112 patients (MFT = 62 and MPT = 50). Intervention took place during inpatient multimodal treatment. Both MPT and MFT interventions “promoted an autonomy-supportive parental attitude and the adolescents’ autonomy and self-determination.” Parents were encouraged to “create the conditions supporting their daughters’ autonomy in establishing healthy eating at home to indirectly increase their daughters’ motivation”. Group format was one introductory 3-h session followed by five 2-h sessions every 2 weeks. Measures were taken pre/post the intervention. Patients were not randomized, but rather allocation to MFT vs MPT depended on time of admission. Results reported a main effect of time for drive for thinness (*p* < 0.001) and body dissatisfaction (*p* < 0.001) as measured by EDI-2. Both scales improved independent of type of intervention.	⨁◯◯◯ VERY LOW	IMPORTANT
Change in EDI score									
1	Case series	very serious ^a^	not serious	not serious	not serious	none	One case series describing the addition of Family-Oriented Group Therapy to an inpatient sample of 32 adolescent patients (29 with AN, 3 with BN). Improvements in EDI scores were noted.	⨁◯◯◯ VERY LOW	IMPORTANT

Explanations

^a^Due to design, no blinding possible

Bibliography:

Case Control - Depestele 2017 [236]

Case Series – Salbach 2006 [237]

#### Meal support

##### Mixed diagnoses

Three studies were found that examined the effect of meal support/supervision as part of inpatient treatment for groups of patients with mixed eating disorders diagnoses [238–240] (Table 48). There were no significant differences between cohorts who received meal support and those who did not on the rate of weight gain per day or week, although there was a trend towards greater weight gain in the group who received meal support. One of these studies reported on the difference in the rate of nasogastric tube (NGT) feeds in the cohort of patients treated on inpatient unit before the institution of consistent meal support versus after [238]. Eight of 12 patients not receiving meal support (66.7%) and 1 of 9 (11.1%) of those receiving meal support required NGT feeds as part of inpatient admission, which was a statistically significant difference.

**Table 48:** Meal support during inpatient treatment versus no meal support be used in the treatment of children and adolescents with eating disorders

Certainty assessment							Impact	Certainty	Importance
№ of studies	Study design	Risk of bias	Inconsistency	Indirectness	Imprecision	Other considerations			
Rate of Weight Gain (assessed with: Measures of Weight Gain in Kg/Day), Need for NGT Feeds (assessed with: # of Patients Receiving NGT Feeds)									
3	Case Control	serious ^a^	serious ^b^	not serious	serious ^c^	all plausible residual confounding would reduce the demonstrated effect	Three studies examined the effect of meal support/supervision as part of inpatient treatment for a total number of patients receiving meal support of 88 patients. There were no significant differences between cohorts who received meal support and those who did not on the rate of weight gain per day or week,although there was a trend towards greater weight gain /day or week in the group who received meal support. Weight gain varied from 0.09 kg/day to 0.35 kg/day across studies.	⨁◯◯◯ VERY LOW	CRITICAL
		serious ^d^	not serious	not serious	not serious	strong association all plausible residual confounding would reduce the demonstrated effect	One study of these studies reported on difference in the rate of NGT feeds in cohort of patients treated on inpatient unit before the institution of consistent meal support vs after. 8/12 patients not receiving meal support (ie 66.7%) and 1/9 (11.1%) of those receiving meal support required NGT feeds as part of inpatient admission.	⨁⨁⨁◯ MODERATE	IMPORTANT

Explanations

^a^Differences in LOS and age between those receiving meal support and those not receiving meal support may have affected outcomes

^b^Wide variation in # of meals/day supervised between various studies

^c^Wide confidence intervals in some studies/groups

^d^Criteria for initiating NGT feeds somewhat vague (ie “consistent failure” to meet expected weight gain and/or acute refusal of food

Bibliography:

Case Control - Kells 2013 [239], Kells 2017 [240], Couturier 2009 [238]

#### Selective versus non-selective menus

##### Anorexia nervosa

One study was found which included 22 patients with AN who received non-selective menus compared to 18 patients who received selective menus as part of their multimodal inpatient treatment [241] (Table 49). Length of stay varied between groups (although non-significantly) with patients on non-select menus remaining in hospital a mean of 60.3 (+/− 22.8) days vs 74.2 (+/− 28.7) days in the selective menus group. The non-selective menu group gained a significantly greater amount of weight. No significant differences were found on the EDE.

**Table 49:** Non-selective menus during inpatient treatment versus selective menus for children and adolescents with anorexia nervosa

Certainty assessment							Impact	Certainty	Importance
№ of studies	Study design	Risk of bias	Inconsistency	Indirectness	Imprecision	Other considerations			
Rate of Weight Gain (assessed with: Weekly weight gain in kg/week), EDE Scores									
1	Case Control	serious ^a,b^	not serious	not serious	serious ^c^	all plausible residual confounding would reduce the demonstrated effect	One study including 22 patients who received non-selective menus compared to 18 patients who received selective menus. LOS varied between groups (although non-significant) with non-select patients remaining in hospital a mean of 60.3 (+/− 22.8) days vs 74.2 (+/−28.7) days in selective menus group. Non-selective menu group gained a mean of 0.95 kg/wk (+/−0.35) and those in selective menu group gained a mean of 0.72 kg/wk (+/− 0.24) (p = 0.02).	⨁◯◯◯ VERY LOW	CRITICAL
		serious ^a,b^	not serious	not serious	serious ^c^	all plausible residual confounding would reduce the demonstrated effect	No significant differences were found on any of the EDE items related to eating concern. Overall change in EDE eating concern scores were low ranging from −0.6 to 1.1.	⨁◯◯◯ VERY LOW	IMPORTANT

Explanations

^a^Unclear whether groups differed at baseline as these details were not reported

^b^Cohort study design (pre/post introduction of non-selective menus), unclear if other aspects of care may have also varied between groups

^c^Confidence intervals relatively wide and overlap with actual difference in effect

Bibliography:

Case Control - Leacy 2012 [241]

#### Bright light therapy

##### Anorexia nervosa and major depressive disorder

One study of patients with AN and depressive symptoms admitted to a CBT-based inpatient program and treated adjunctively with Bright Light Therapy was found [242] (Table 50). In this study patients were randomized to receive either daily 30 min Bright Light Therapy (BLT) + inpatient treatment (*n* = 12) for 6 weeks or inpatient treatment only for 6 weeks (*n* = 12). Patients in both groups had a significant change in their BMI during the 6-week study, however change from baseline was statistically significant by week 3 (*p* = 0.038) in BLT group versus by week 6 (*p* = 0.048) in the comparison group.

**Table 50:** Bright light therapy during CBT-based inpatient treatment versus CBT-based inpatient treatment alone for children and adolescents with anorexia nervosa and major depressive disorder

Certainty assessment							Impact	Certainty	Importance
№ of studies	Study design	Risk of bias	Inconsistency	Indirectness	Imprecision	Other considerations			
RCT - Change in Weight (assessed with: Change in BMI per week)									
1	randomised trials	serious ^a^	not serious	not serious	serious ^b^	none	One study randomized patients with AN-R and depressive symptoms (> 17 on HDRS) admitted to CBT-based inpatient treatment to receive either daily 30 min BLT + inpatient treatment (*n* = 12) × 6 weeks or inpatient treatment only × 6 weeks (*n* = 12). Patients in both groups had a significant change in their BMI during 6 week study, however change from baseline was statistically significant by week 3 (*p* = 0.038) in BLT group vs only significant change from baseline at week 6 (*p* = 0.048) in TAU group.	⨁⨁◯◯ LOW	CRITICAL

Explanations

^a^No blinding of subjects to treatment group

^b^Confidence intervals overlapping with actual size of treatment effect

Bibliography

RCT - Janas-Kozik 2011 [242]

#### Cognitive remediation therapy

##### Anorexia nervosa

Four studies reported on the addition of Cognitive Remediation Therapy (CRT) to multimodal inpatient treatment [243–246] (Table 51). One study described change in weight between patients who received 10 sessions of CRT over 10 weeks versus those who received TAU in a quasi-experimental design (*n* = 24 in each group) [244]. Both groups gained weight at a similar rate. The other studies reported on patients (total 79 patients) who received either 4 or 10 sessions of CRT provided as once weekly sessions. In all three studies patients gained weight. Given the design of these last three studies it was not possible to determine whether CRT had an impact on weight above and beyond what would have been expected by inpatient treatment alone.

**Table 51:** Cognitive remediation therapy during inpatient treatment be used for the treatment of children and adolescents with anorexia nervosa

Certainty assessment							Impact	Certainty	Importance
№ of studies	Study design	Risk of bias	Inconsistency	Indirectness	Imprecision	Other considerations			
Change in Weight (assessed with: Pre/Post CRT Measures of Weight), change in EBRS, change in EDE-Q									
3	Case/control Case Series	very serious ^a^	not serious	not serious	not serious	all plausible residual confounding would reduce the demonstrated effect	Three studies reported on addition of CRT to multimodal inpatient treatment. One study described change in weight between patients who received 10 sessions of CRT over 10 weeks vs those who received TAU in a quasi-experimental design (*n* = 24 in each group). Both groups gained weight at a similar rate (change from mean BMI% of 2.2 to 5.7 over 10 weeks in CRT group vs mean BMI% 5.5 to 7.6 over 10 weeks in TAU group). The other studies reported on patients (total 79 patients) who received either 4 or 10 sessions of CRT provided as once weekly sessions. In all 3 studies patients gained weight. Given the design of these studies it is not possible to determine whether CRT had an impact on weight above and beyond what would have been expected by inpatient treatment alone.	⨁◯◯◯ VERY LOW	CRITICAL
		very serious ^b^	not serious	not serious	not serious	all plausible residual confounding would reduce the demonstrated effect	One study included description of 2 patients who received 10 sessions of CRT over 10 weeks in addition to multimodal inpatient treatment. EBRS scores decreased slight for both patients from 26 to 22 and 29 to 26 at end of 10 weeks.	⨁◯◯◯ VERY LOW	IMPORTANT
		very serious ^a^	not serious	not serious	serious ^d^	all plausible residual confounding would reduce the demonstrated effect	One study, including 125 hospitalized patients. Received either group (*n* = 55) or individual (*n* = 70) CRT. Only those patients receiving individual CRT completed the EDE-Q. pre-post. Patients receiving individual CRT did not experience a change in their EDE-Q global score over the course of the 10 weeks where they received CRT.	⨁◯◯◯ VERY LOW	IMPORTANT
		very serious ^a^	not serious	not serious	not serious	all plausible residual confounding would reduce the demonstrated effect	One study, comprising 70 hospitalized patients who received multimodal inpatient treatment along with 10 individual sessions of CRT over 10 weeks. Patients completed the MSCARED before and after the course of CRT. There was a statistically significant shift in motivation noted (*p* < 0.001), where at initiation of CRT % of patients in each stages of change category were as follows: pre-contemplation 18.6%, contemplation 38.6%, preparation 28.6%, action 11.4%, maintenance 2.9%. At the end of CRT % of patients in each stage of change were: pre-contemplation 0%, contemplation 4.3%, preparation 31.4%, action 42.9%, maintenance 21.4%, Due to the design of this study it is not possible to differentiate the effect of inpatient treatment alone from inpatient treatment + CRT.	⨁◯◯◯ VERY LOW	IMPORTANT
		very serious ^b^	serious ^c^	not serious	not serious	all plausible residual confounding would reduce the demonstrated effect	One study included description of 2 patients who received 10 sessions of CRT over 10 weeks in addition to multimodal inpatient treatment. Scores on EAT decreased for one patient (30 to 16) and increased in the other patient (35 to 36).	⨁◯◯◯ VERY LOW	IMPORTANT
Weight, EAT-26									
2	Case reports	very serious ^b^	serious ^c^	not serious	not serious	all plausible residual confounding would reduce the demonstrated effect	One study involved 7 adolescentsinpatients with AN using group CRT. Weight improved as did motivation.	⨁◯◯◯ VERY LOW	IMPORTANT
		very serious ^b^	serious ^c^	not serious	not serious	all plausible residual confounding would reduce the demonstrated effect	Another study is a single case report describing improvement on the EAT-26 after 10 sessions of CRT with an inpatient with AN.	⨁◯◯◯ VERY LOW	IMPORTANT

Explanations

^a^Not all studies had comparison group and were receiving inpatient treatment which could account for some of the differences observed/reported

^b^Case report design, no comparison/control

^c^Differing results between the 2 reports likely secondary to individual differences

^d^Wide confidence intervals, overlapping with with the size of the effect noted

Bibliography:

Case control - Herbrich 2017 [244], Harrison 2018 [246]

Case series – Asch 2014 [243]

Case reports – Kuge 2017 [245], Cwojdzinska 2009 [247]

Several studies of CRT added to inpatient treatment for AN reported on symptom change. One study included a description of two patients who received 10 sessions of CRT over 10 weeks in addition to multimodal inpatient treatment [243]. Scores on EAT decreased for one patient (30 to 16) and increased in the other patient (35 to 36). One study, including 125 hospitalized patients [246], received either group (*n* = 55) or individual (*n* = 70) CRT. Only those patients receiving individual CRT completed the EDE-Q pre-post. Patients receiving individual CRT did not experience a change in their EDE-Q global score over the course of the 10 weeks they received CRT. One additional case report describes 10 sessions of CRT delivered to an inpatient with AN. Improvements on the EAT were observed [247].

One study, comprising 70 hospitalized patients who received multimodal inpatient treatment along with 10 individual sessions of CRT over 10 weeks reported on change in motivation as measured by the Motivational Stages of Change for Adolescents Recovering from an Eating Disorder (MSCARED) [246]. Patients completed the MSCARED before and after the course of CRT. There was a statistically significant improvement in motivation noted. Due to the design of this study it was not possible to differentiate the effect of inpatient treatment alone from inpatient treatment plus CRT.

#### Inpatient and day treatment combined

##### Anorexia nervosa

Five reports on 265 patients with AN treated as inpatients followed immediately by day treatment were found [200, 248–251] (Table 52). In all five studies, patients were treated as inpatients and then transferred to day treatment once medically stable. Details regarding the number of hours/days spent in day treatment were not thoroughly reported, although mean length of stay varied from 7.9 weeks to 3.9 months. Weight change was reported in various ways, however, all studies indicated improvement in weight.

**Table 52:** Inpatient and day treatment in combination for children and adolescents with anorexia nervosa

Certainty assessment							Impact	Certainty	Importance
№ of studies	Study design	Risk of bias	Inconsistency	Indirectness	Imprecision	Other considerations			
Weight Change (assessed with: Change in weight during treatment)									
5	Case Control and Case Series	very serious ^a^	not serious	not serious	not serious	none	265 patients over 5 studies, all with AN. All studies treated patients as inpatients and then transferred to day treatment once medically stable. Details regarding the number of hours/days spent in day treatment not completely reported. Mean LOS were 3.9 months, 15.1 weeks and 7.9 weeks. Patients gained weight as described by BMI in 3 studies where BMI increase from 12.1 (SD1.1) to 18.6 (SD 0.42) in one study, 15.7 (SD1.2) to 18.0 (SD 1.0) in the second study and 15.19 (+/− 1.54) to 17.56 (+/− 1.07) in the third study. The second study also reported weight as %TGW which rose from 77.6% at admission to inpt to 88.5% at end of day treatment. In 2 studies weight change was described using BMI centiles and weight rose from a mean BMI centile of 2.7 (+/− 4.2) to 34.2 (+/− 15.7) in one study and 1.6 (+/− 5.1) to 49.4 (+/− 3.9) in the second study. 5/40 patients eligible for one study left treatment AMA and were not included in analysis.	⨁◯◯◯ VERY LOW	CRITICAL
Eating Disorder Inventory - 2 Score at discharge (assessed with: Rating Scale)									
1	Case Series	very serious ^a,b^	not serious	not serious	not serious	none	35 patients completed inpt, day treatment and measures. Mean LOS 15.1 weeks. Change in ED1–2 total, drive for thinness and body dissatisfaction not significantly different between admission and discharge.	⨁◯◯◯ VERY LOW	IMPORTANT
Anorexia Nervosa Stages of Change Questionnaire (assessed with: Rating Scale)									
1	Case Series	very serious ^a,c^	not serious	not serious	serious ^d^	all plausible residual confounding would reduce the demonstrated effect	35 patients completed ANSOCQ at admission and d/c (ie after 15.1 weeks of inpatient + day treatment). Overall score increased a mean of 21.7 points which would signify moving from contemplation to preparation phases. Overall 29.4% (up from 0% at admission) of patients were classified as in “maintenance phase” and 26.5% (up from 15% at admission) in “action phase” at time of discharge.	⨁◯◯◯ VERY LOW	IMPORTANT
Overall Outcome (assessed with: Rating combining weight + compensatory symptoms)									
1	Case series	very serious ^a,e^	not serious	not serious	not serious	all plausible residual confounding would reduce the demonstrated effect	One study which included 71 patients who completed 7.9 weeks of combined inpatient and DTP (33 days inpatient and 22 days DTP). At end of DTP 35.2% were deemed to have an excellent outcome (> 90% ideal BMI, maintaining weight and no use of compensatory symptoms in last week of program), 26.8% were deemed good outcome (85–90% ideal BMI, maintaining weight and no use of compensatory symptoms in last week of treatment), 14.1% deemed below average outcome (80–85% ideal BMI and maintaining weight OR > 85% ideal BMI, but losing 0.15–0.45 kg/week with no compensatory symptoms in the last week of treatment) and 23.9% were deemed to have a poor outcome (either < 80% ideal BMI OR < 85% ideal BMI and losing > 0.15 kg/week OR readmitted to inpatient unit OR use of compensatory symptoms in the last week of treatment).	⨁◯◯◯ VERY LOW	IMPORTANT
Eating Disorder Symptomatology (assessed with: Pre-post EDE-Q)									
1	Case series	very serious ^a^	not serious	not serious	serious ^d^	none	One study, including *n* = 26 adolescents. Patients received 13 weeks of inpatient treatment based on CBT-E model followed by 7 weeks of DTP. EDE scores decreased significantly pre-post for global and all subscales other than Shape Concern. Global EDE at admission 3.7 (+/− 1.3) to d/c 2.0 (+/− 1.1), % of patients with Global EDE < 1 SD above the community mean at admission 2% (+/− 7.7) and at d/c 10% (+/− 38.5). Dietary restraint at admission 4.1 (+/− 1.2) and at d/c 1.1 (+/− 1.0), Eating Concern (3.3 (+/− 1.4) and at d/c 1.5 (+/− 1.4), Shape Concern (3.8 (+/− 1.8) and at d/c 3.2 (+/− 1.4), Weight Concern at admission 3.5 (+/− 1.9) and at d/c 2.3 (+/− 1.4).	⨁◯◯◯ VERY LOW	IMPORTANT
Change in Frequency of Eating Disorder Symptoms (assessed with: Pre-post ED symptom frequency)									
1	Case series	very serious ^a^	not serious	not serious	serious ^d^	none	One study, including *n* = 26 adolescents. Patients received 13 weeks of inpatient treatment based on CBT-E model followed by 7 weeks of DTP. Binge eating was present in 8 patients (30%) at admission and only 2 patients (7.7%) at discharge. Median frequency of bingeing in previous 28 days was 17 (range 2–148) at admission and 8 (range 1–15) at discharge. Purging by vomiting was present at admission for 10 patients (28.5%) and at d/c for 4 patients (15.1%). Frequency of vomiting in previous 28 days was 25 (range 1–196) at admission and 10.5 (range 0–30) at dscharge. Laxative misuse was present for 3 patients at admission and none at discharge. Frequency of laxative abuse in previous 28 days was 1 (range 1–20) at admission and nil at d/c.	⨁◯◯◯ VERY LOW	CRITICAL

Explanations

^a^Observational study with no comparison/control

^b^Self-rating scale (EDI-2)

^c^Self-rating scale (ANSOCQ)

^d^Lower end of confidence interval overlaps with score that would signify no change

^e^Information on compensatory symptoms was taken only from clinician notes

Bibliography:

Case control - El Ghoch 2015 [250], Strober 2006 [248]

Case series - Delle Grave 2014 [249], Hillen 2015 [251], Treat 2008 [200]

Symptom change was reported using various scales in these studies. One study included 35 patients with a mean length of stay of 15.1 weeks [251]. Change in EDI total, drive for thinness and body dissatisfaction were not significantly different between admission and discharge. One study included 26 adolescents who received 13 weeks of inpatient treatment based on the Cognitive Behavioural Therapy- Enhanced (CBT-E) model followed by 7 weeks of Day Treatment Program (DTP) [249]. EDE scores decreased significantly pre-post for global score and all subscales other than Shape Concern. This study also reported on frequency of eating disorder symptoms. Binge eating was present in eight patients (30%) at admission and only two patients (7.7%) at discharge. Purging by vomiting was present at admission for 10 patients (28.5%) and at discharge for 4 patients (15.1%). Laxative misuse was present for 3 patients at admission and none at discharge.

One study reported on change in motivation as measured by the ANOSCQ in 35 patients [251]. These patients received 15.1 weeks of inpatient and day treatment. Overall scores increased a mean of 21.7 points, which signified moving from contemplation to preparation phases.

One study which included 71 patients who completed 7.9 weeks of combined inpatient and DTP (33 days inpatient and 22 days DTP) reported on “overall outcome” [200]. At the end of DTP 35.2% were deemed to have an excellent outcome, 26.8% were deemed good outcome, 14.1% deemed below average outcome and 23.9% were deemed to have a poor outcome.

#### Admission to weight restoration versus short admission for medical stabilization with either FBT or day treatment

##### Anorexia nervosa

Two high quality studies examined the difference between patients randomized to receive a relatively short inpatient admission followed by either 20 sessions of FBT (*n* = 82) [252] or Day Treatment (*n* = 172) [253] compared to a lengthy inpatient stay to weight restoration (Table 53). In the Inpatient/FBT study [252] patients had all been unwell less than 3 years and in the inpatient/day treatment study [253] the patients were included only if it was their first admission. At the end of FBT or Day Treatment, there were no significant differences between those who were discharged after a short admission versus those who remained in hospital for weight restoration in terms of weight outcome, rate of readmissions over 12-month follow-up, or eating disorder symptoms [252, 253].

**Table 53:** Inpatient medical stabilization followed by outpatient treatment versus inpatient weight restoration for children and adolescents with anorexia nervosa

Certainty assessment							Impact	Certainty	Importance
№ of studies	Study design	Risk of bias	Inconsistency	Indirectness	Imprecision	Other considerations			
Change in weight (assessed with: Pre/post measures of weight), Rate of Readmission, psychological symptoms (EDE and EDI)									
2	randomised trials	serious ^a^	not serious	not serious	not serious	none	Two RCT examined the difference between patients randomized to receive a relatively short inpatient admission followed by either 20 sessions of FBT (*n* = 82) or DTP (*n* = 172). In the FBT F/U study patients had all been unwell less than 3 years and in the day treatment F/U study the patients we only included if it was their first admission. The first study randomized patients to be d/c once medically stable (mean LOS 21.73 +/− 5.92 days) vs to remain in hospital until 90%TGW (mean LOS 36.89 +/− 17.06). Both groups received 20 sessions of FBT following discharge. Patients discharged at point of medical stability (ie mean 84.40%TGW) had attained a mean of 95.20%TGW by the end of 20 sessions of FBT, whereas those who remained in hospital until they were 90%TGW (ie mean 92.00%TGW) were at a mean of 93.10%TGW by session 20. ES in this study was 1.28 at the end of hospitalization and 0.27 at end of session 20. There was a significant difference in weight at end of hospitalization, but not by end of session 20. The other study randomized patients to remain as inpatients for 3 weeks vs until attaining TGW (total mean treatment time 14.6 weeks). Those d/c at 3 weeks entered a DTP with similar programming (total mean treatment time 16.5 weeks). At end of treatment patients in inpatient only group had reached a mean of 89%TGW (+/− 3.8) and those in inpt + DTP had reached 88.1%TGW (+/− 4.7) - no significant difference in weight outcome in intention to treat analysis.	⨁⨁⨁◯ MODERATE	CRITICAL
		serious ^a^	not serious	not serious	not serious	none	Both RCTs examined Rate of readmission measured over the 12 months following admission in the FBT-f/u study (*n* = 82). Readmission rates were similar regardless of allocation (ie 35% in med stability group vs 36.8% in the weight restoration group). However, given that the med stability group had a shorter initial admission, the total hospital days was 45.2 d in this group vs 65.5 in the weight restoration group. In inpatient weight restoration vs DTP F/U 8/87 patients were readmitted during their DTPtreatment due to medical instability and 25.3% (inpt WR) vs 15.1% (DTP), *p* = 0.12 required readmission to inpatient unit at 12 months F/U.	⨁⨁⨁◯ MODERATE	IMPORTANT
		serious ^a^	not serious	not serious	not serious	none	One study - EDI-2 scores pre treatment and post treatment similar between groups regardless of allocation (total *n* = 143).	⨁⨁⨁◯ MODERATE	IMPORTANT
		serious ^a^	not serious	not serious	not serious	none	One study - EDE global scores not significantly different between groups at baseline or at end of FBT, 6 month or 12 month F/U (*n* = 69).	⨁⨁⨁◯ MODERATE	IMPORTANT

Explanations

^a^No blinding of participants possible

Bibliography:

RCT - Herpertz-Dahlmann 2014 [253], Madden 2015 [252]

### Day treatment

#### Multimodal day treatment

##### Anorexia nervosa

Two case series and one case report describe the outcomes of patients treated in their multimodal day hospital programs [254–256] (Table 54). Admission to day treatment in these studies could occur from an inpatient setting or an outpatient setting based on clinical need. Weight related outcomes were reported in various ways. Improvements in BMI from admission to discharge were described [255]. Two studies reported improvements in %TGW at admission and discharge [255, 256]. One study reported an increase in weight from 81.6 to 84.2%TGW [255]. The other study reported weight change separately for patients above and below 85%TGW at admission and found both cohorts gained weight [256]. Mean length of stay varied between 70 to 92 days. One case report described a weight change from 87 lbs to 101 lbs over the admission to the day program [254].

**Table 54:** Multimodal day treatment be used in the treatment of children and adolescents with anorexia nervosa

Certainty assessment							Impact	Certainty	Importance
№ of studies	Study design	Risk of bias	Inconsistency	Indirectness	Imprecision	Other considerations			
Weight Gain From Admission to Discharge (assessed with: BMI/%TGW/Wt), EDI-3, EAT-26, Motivation, successful completion (%)									
2	Case Series	very serious ^a^	serious ^b^	not serious	serious ^c^	all plausible residual confounding would reduce the demonstrated effect	Admission to DTP could occur from inpatient setting or outpatient setting based on clinical judgment of need for this level of care. Weight related outcomes reported in various ways. One study reported admission and discharge BMI with a change from 16.5 (SD 1.5) to 17.1 (SD 1.9). Two studies reported %TGW at admission and discharge. One reported an increase in weight from 81.6 to 84.2%TGW. The other study reported weight change separately for patients above and below 85%TGW at admission. For those < 85%TGW at admission, TGW rose from 81.5 to 88.3%, in those > 85%TGW at admission %TGW rose from 88.0 to 92.2%.. Mean LOS varied between 70 to 92 days.	⨁◯◯◯ VERY LOW	CRITICAL
		very serious ^a^	not serious	not serious	serious ^c^	all plausible residual confounding would reduce the demonstrated effect	One study - 26 patients in study, remained in DTP for mean LOS of 10 weeks. Eighty-five % of patients were referred to DTP from outpatient setting, remainder from inpatient program. Only criterion from admission to DTP vs inpatient was medical stability. EDI-3 scores for Drive for Thinness and Perfectionism improved significantly with Drive for Thinness changing from 13.81 (SD 9.08) to 10.08 (SD 8.32) and Perfectionism changing from 8.96 (6.79) to 8.19 (SD 6.87), signifying a small effect size (0.43 and 0.11 respectively). Body dissatisfaction and maturity fears did not change significantly during course of DTP.	⨁◯◯◯ VERY LOW	IMPORTANT
		very serious	not serious	not serious	serious ^c^	all plausible residual confounding would reduce the demonstrated effect	One study - 26 patients in study, remained in DTP for mean LOS of 10 weeks. EAT-26 scores decreased from 28.08 (SD 20.61) at admission to 22.19 (SD 19.34) at discharge which signifies a small effect size (ie 0.30).	⨁◯◯◯ VERY LOW	IMPORTANT
		very serious ^a^	not serious	not serious	serious ^c^	all plausible residual confounding would reduce the demonstrated effect	One study - 26 patients in study, remained in DTP for mean LOS of 10 weeks. ANSOCQ score changed from 53.48 (SD 20.42) to 65.63 (SD 21.27) signifying no change in “stage” (patients remained in “preparation phase” throughout).	⨁◯◯◯ VERY LOW	IMPORTANT
		very serious ^a^	serious ^b^	not serious	not serious	none	Two studies reported on “% completing” the DTP, including 53 patients with AN. Definition of “successful completion” was based on a combination of symptom change, weight gain and progression in program (vs leaving AMA or need for admission to inpatient unit). Mean LOS ranged from 11.6 to 15.3 weeks. “Successful Completion” rates in these studies were 30 to 50%. One study examined whether completion rate varied between those that started at greater than or less that 85%TGW, and reported that there was no difference based on this factor.	⨁◯◯◯ VERY LOW	CRITICAL
Weight									
1	Case report	very serious ^a^	serious ^b^	not serious	serious ^c^	all plausible residual confounding would reduce the demonstrated effect	The case report described a weight change from 87lbs to 101 lbs. over the DTP admission	⨁◯◯◯ VERY LOW	CRITICAL

Explanations

^a^ Observational study, no comparison/control

^b^Varying BMI/TGW at admission to various programs, programs provided differing levels/hours of support and results on this outcome varied

^c^Confidence intervals wider than actual effect in some studies

Bibliography:

Case series - Ngo 2014 [256], Goldstein 2011 [255]

Case reports – Garner 2002 [254]

One study examined eating disorder psychological symptoms with 26 patients remaining in DTP for mean length of 10 weeks [255]. EDI scores for Drive for Thinness and Perfectionism improved significantly, whereas body dissatisfaction and maturity fears did not change significantly.

Two studies reported on percent of patients successfully completing the day treatment program [255, 256]. Definition of “successful completion” was based on a combination of symptom change, weight gain and progression in program (versus leaving against medical advice (AMA) or need for admission to an inpatient unit). Mean length of stay ranged from 11.6 to 15.3 weeks. Successful completion rates in these studies were 30 to 50%. One study examined whether completion rate varied between those that started at greater than or less that 85%TGW, and reported that there was no difference based on this factor [256].

##### Mixed diagnoses

Several studies address mixed diagnoses of eating disorders within a multimodal day hospital program [257–261] (Table 55). Weight in all studies improved over the course of day treatment. Weight gain was correlated with a diagnosis of AN or EDNOS (versus BN), longer length of stay and lower weight at admission [261]. The length of stay in these studies varied between 15.3 weeks and 13.1 weeks.

**Table 55:** Multimodal day treatment be used in the treatment of children and adolescents with eating disorders

Certainty assessment							Impact	Certainty	Importance
№ of studies	Study design	Risk of bias	Inconsistency	Indirectness	Imprecision	Other considerations			
Weight Change (assessed with: BMI/TGW), Change in self esteem, successful completion, change in motivation									
5	Case series	very serious ^a^	not serious	not serious	serious ^b^	none	Reasons for referral to DTP were based on severity of symptomatology, but could occur from inpatient or outpatient or initial assessment. Two studies reported all patients together, the other (*n* = 160) reported AN (*n* = 116) vs BN (*n* = 44). In one mixed study the mean BMI rose from 18.9 (SD 2.6) to 20.9 (SD 2.9) which related to a change in %TGW from 94% at admission to 102% at discharge. In the other mixed study the weight gain was reported as 0.95 kg over the 2.6 weeks LOS. It was noted that approx one-quarter of patients lost weight, one quarter gained 0–0.9 kg, on quarter gained 0.9–1.8 kg and one quarter gained > 1.8 kg. Weight gain was correlated with dx of AN or EDNOS vs BN, longer LOS and lower weight at admission. The last study reported that patients with AN started at a mean BMI of 18.3 (SD 1.2) and gained 0.9 points, whereas patients with BN started with a mean BMI of 20.3 (SD 3.3) and gained a mean of 0.3 points. The LOS in these studies was 15.3 weeks and 13.1 weeks respectively.	⨁◯◯◯ VERY LOW	CRITICAL
		very serious ^a^	not serious	not serious	serious ^b^	all plausible residual confounding would reduce the demonstrated effect	One study - Total of 160 patients (ie 116 AN patients and 44 BN patients). Mean LOS was 15 weeks. For AN group the SEED in relation to others decreased from 16.5 (SD 9.7) to 15.0 (SD 10.7) (*p* = 0.039) and SEED related to weight and shape changed from 14.6 (SD 7.8) to 13.5 (SD 9.0) (*p* = 0.046). In the BN group SEED in relation to others changed from 17.3 (SD 7.8) to 13.2 (SD 8.5) (*p* = 0.000) and SEED related to weight and shape changed from 17.6 (SD 7.0) to 13.2 (SD 8.0) (*p* = 0.001). No significant difference in effect between AN and BN.	⨁◯◯◯ VERY LOW	IMPORTANT
		very serious ^a^	not serious	not serious	not serious	none	Two studies for total of 61 patients. Success defined using various criteria such as adequate weight gain, symptom reduction, and no AMA discharge or inpatient admission. “Success” rate was 49 and 50% in these 2 studies.	⨁◯◯◯ VERY LOW	IMPORTANT
		very serious ^a^	not serious	not serious	not serious	all plausible residual confounding would reduce the demonstrated effect	One study including 30 patients. LOS was 10.5 weeks. Motivational Stage of Change was measured pre-post with the MSCARED. Patients were noted to progress through 1.9 +/− 1.3 stages from beginning to end of treatment (*p* < 0.0001). The change in SOC from intake to discharge was significantly correlated with the change in the ChEAT score during the same time period (*p* = 0.001).	⨁◯◯◯ VERY LOW	IMPORTANT

Explanations

^a^Observational study with no comparison/control

^b^Confidence intervals wider than effect size

Bibliography:

Case series - Bustin 2013 [260], Lazaro 2011 [259], Dancyger 2002 [257], Dancyger 2003 [258], deGraft-Johnston 2013 [261]

Lazaro and colleagues [259] reported outcomes separately for those with AN and BN within their day treatment program. The sample size was 160 patients (116 AN patients and 44 BN patients). Mean length of stay was 15 weeks. For both groups, self-esteem improved in relation to others and in relation to weight and shape. No significant differences were found between the AN and BN groups [259].

Two studies treating mixed diagnoses of eating disorders for total of 61 patients looked at successful completion of the program [257, 258]. Success was defined using various criteria such as adequate weight gain, symptom reduction, and no AMA discharge or inpatient admission. Success rate was 49% [258] and 50% [257].

One study including 30 patients with mixed diagnoses examined motivational stage of change [260]. Length of stay was 10.5 weeks. Motivational Stage of Change was measured pre-post with the MSCARED [260]. Patients were noted to progress through the stages of change during treatment. The change in stage of change from intake to discharge was significantly correlated with the change in the Children’s Eating Attitudes Test (ChEAT) score during the same time period [260].

#### Family-based day treatment

##### Anorexia nervosa/low weight eating disorders

Nine studies for a total of 427 patients examined a family-based day treatment program [262–270] (Table 56). Studies varied with regards to the degree to which they included parents in treatment, number of hours/week of programming and length of stay. Criteria for admission to the day treatment program varied.

**Table 56:** Family-based day treatment for children and adolescents with anorexia nervosa and low-weight eating disorders

Certainty assessment							Impact	Certainty	Importance
№ of studies	Study design	Risk of bias	Inconsistency	Indirectness	Imprecision	Other considerations			
Change in Weight (assessed with: Pre-post change in weight outcomes), Change in EDE-Q scores, change in symptoms									
9	Case Control and Case Series	very serious ^a^	serious ^b^	not serious	serious ^c^	strong association all plausible residual confounding would reduce the demonstrated effect	Nine studies for a total of 427 patients. Studies varied with regards to degree/method of including parents in treatment, # of hours/week of programming and LOS. Criteria/reasons for admission to the DTP program varied, studies which reported referral source/reasons described that patients could be referred from either initially assessment, inpatient or outpatient based on the severity of their symptoms. Five studies reported on change in BMI which rose from 17.5 (SD 0.4) to 19.5 (SD 0.4), 16.4 to 19.6, 16.3 (+/−1.6) to 17.3 (+/− 1.3), 17.01 (range 12.3–22.1) to 20.05 (range 14.8–25.1), and 16.2 (+/− 1.98) to 19.4 (+/−2.87). Three studies reported on total weight gained in program (8.6 kg +/− 4.5 kg; 5.0 kg +/− 2.5; 7.3 kg +/− 3.1 and 17.58 kg). Two studies reported on change in %TGW which rose from 82.56 to 93.00% in one study and 82.3 to 97.99%. LOS in these studies varied from 27.6 (SD 12.13) days to 1.3 (SD 0.2) years. One study reported on difference in weight outcomes between their Maudsley and non-Maudsley DTP, noting no difference between these 2 groups. One other study reported on differences between patients who received “formal psychotherapy” (individual and/or family) outside of program thereby needing to leave program for approx 2 h/week and noted that patients who received psychotherapy within the first 2 months of entering DTP gained significantly less weight (ie 5.0 +/− 2.5 kg vs 7.3 +/− 3.1 kg). One study examined predictors of weight restoration in DTP and reported that Higher BMI at admission (range 12.3–22.1), greater gain in %TGW in first 4 weeks (range − 0.18 to 25.27% TGW) and lower caregiver empowerment at baseline were predictive of weight restoration at end of intensive treatment (ie DTP + IOP).	⨁◯◯◯ VERY LOW	CRITICAL
		very serious ^d^	not serious	not serious	not serious	all plausible residual confounding would reduce the demonstrated effect	Five studies receiving a family-based DTP treatment. LOS was 37.05 days, 28.41 days (SD 13.55) over 11.7 weeks (patients did not attend every day as they were transitioning back to school), 27.6 days (SD 12.13) and 11.56 days (SD 6.61), and one was a 3 month follow up. Weight at onset in 4 studies were similar although reported in different ways (ie 80.94%TGW in first study, BMI 16.3/79.9% in the second study, 82.56% in third study and BMI 16.4 in forth study). EDE scores, global and all subscales decreased significantly in all studies, although confidence intervals overlapped with size of effect. In the study reporting on a control group which was treated in the same program, but without the inclusion of Maudsley/family interventions, the EDE-Q scores decreased more in the Maudsley group than the non-Maudsley as the Maudsley group started with higher EDE-Q scores and at the end of the treatment period their scores were similar to the non-Maudsley. Of note the scores for Wt Concern and Restraint Concerns did not change significantly in the non-Maudsley group whereas they decreased significantly in the Maudsley group.	⨁◯◯◯ VERY LOW	IMPORTANT
		very serious ^e^	not serious	not serious	not serious	all plausible residual confounding would reduce the demonstrated effect	One study consisted of 32 patients. LOS not reported in study. Body image disturbance disappeared completely in 59%, decreased partially in 28% and remained unchangedin 13%. Prolonged duration of meals improved during treatment and “normalized” in 87.5% by end of treatment. Eighty-seven percent stopped ritualistic exercise habits by end of treatment.	⨁◯◯◯ VERY LOW	IMPORTANT
		very serious ^a^	not serious	not serious	serious ^c^	all plausible residual confounding would reduce the demonstrated effect	One study including 60 patients, LOS median stay 8 months (SD 2.27). Statistically significant change was reported in EDI-3 Drive for Thinness (53.40 +/− 35 to 30.68 +/− 31.70) and Dissatisfaction (50.88 +/− 27.60 to 31.62 +/− 29.80), *p* < 0.001.	⨁◯◯◯ VERY LOW	IMPORTANT
		very serious ^a^	not serious	not serious	serious ^c^	all plausible residual confounding would reduce the demonstrated effect	One study including 60 patients, LOS median stay 8 months (SD 2.27). Statistically significant change was reported in EAT-26. Mean EAT-26 score was 26.70 (+/− 17.7) at admission and 7.97 (+/− 11.5) at discharge, *p* < 0.001.	⨁◯◯◯ VERY LOW	IMPORTANT

Explanations

^a^Many studies did not include a control or comparison group

^b^Admission weight, # hours/weeks of treatment, process of family involvement and LOS varied among studies, likely affecting outcome

^c^Confidence intervals wider than effect size in some studies

^d^Only one study included a control comparison, no blinding of participants possible

^e^No validated scale used, no comparison/control group

Bibliography:

Case control - Bean 2010 [264], Danziger 1989 [262]

Case series - Danziger 1988 [263], Gezelius 2016 [265], Martin-Wagar 2019 [269], Rienecke 2016 [266], Rienecke 2018 [267], Simic 2018 [270], Zanna 2017 [268]

Five studies reported improvement in BMI [264, 265, 268–270]. Three studies reported on total weight gained in program [262, 263, 269]. Two studies reported on change in %TGW which rose from 83 to 93% in one study [266], and 83 to 98% in another study [269]. Length of stay in these studies varied from 28 days to 1.3 years.

One study reported on difference in weight outcomes between their Maudsley and non-Maudsley DTP, noting no difference between these two groups [264]. Another study reported on differences between patients who received “formal psychotherapy” (individual and/or family) outside of program thereby needing to leave program for approx 2 h/week and noted that patients who received external psychotherapy within the first 2 months of entering DTP gained significantly less weight [262]. One study examined predictors of weight restoration in DTP and reported that higher BMI at admission, greater gain in the first 4 weeks and lower caregiver empowerment at baseline were predictive of weight restoration at end of intensive treatment [269].

Six studies examined psychological symptoms with the EDE-Q [264, 266, 267, 269–271]. EDE-Q scores, global and all subscales decreased significantly in these studies. In a study reporting on a control group which was treated in the same program, but without the inclusion of Maudsley/family interventions, the EDE-Q scores decreased more in the Maudsley group than the non-Maudsley [264]. Of note the scores for Weight Concern and Restraint Concerns did not change significantly in the non-Maudsley group whereas they decreased significantly in the Maudsley group [264].

One study consisting of 32 patients reported on body image disturbance [263]. Body image disturbance disappeared completely in 59%, decreased partially in 28% and remained unchanged in 13%. Prolonged duration of meals improved during treatment and “normalized” in 87.5% by end of treatment. Eighty-seven percent stopped ritualistic exercise habits by end of treatment.

One study including 60 patients, with median length of stay 8 months showed statistically significant change in EDI Drive for Thinness and body dissatisfaction [268]. Statistically significant change was reported on the EAT.

##### Mixed eating disorder diagnoses

Five case series and one case report for total of 262 patients studied a family-based day treatment program with adolescents with mixed eating disorder diagnoses [272–277] (Table 57). Studies varied with regard to the form of parent involvement, hours/week in treatment and admission criteria. Four studies reported change in BMI from admission to discharge and found that BMI improved [272, 275–277] (Table 57). Three studies reported on change in %TGW and found significant improvements [272, 274, 276]. One study reported weight change as 12/19 patients reaching 100%TGW at 3 months and the other 7/19 reaching a mean %TGW of 94% [273]. The mean LOS varied between these studies from 3.2 weeks to 28.5 weeks. (Table 57).

**Table 57:** Family-based day treatment/intensive outpatient for adolescents with eating disorders

Certainty assessment							Impact	Certainty	Importance
№ of studies	Study design	Risk of bias	Inconsistency	Indirectness	Imprecision	Other considerations			
Improved Weight at Discharge (assessed with: %TGW/BMI), Change in EDE-Q (assessed with: Pre-post EDE-Q scores)									
5	Case Series	very serious ^a^	serious ^b^	not serious	serious ^c^	none	Five studies for total of 254 patients. Studies varied with regard to the form of parent involvement, hours/week in treatment and admission criteria. Referral to receive treatment in DTP or IOP was noted in the studies to be due to the presence of severe symptoms impairing the patients’ functioning or physical health. In some cases the patients had to have already received another form of treatment (ie inpatient or outpatient), but in other cases patients could be referred directly for services in DTP/IOP. Weight related outcomes reported as change in BMI or %TGW. Four studies reported change in BMI from admission to discharge and found that weight rose from 17.4 (SD 2.0) to 18.3 (SD 1.8); 16.5 (SD 2.3) to 18.4 (SD 1.6);18.7 (SD 2.4) to 20.5 (SD 2.0) and by a mean of 0.91+/−0.55 in the final study. Three studies reported on change in %TGW and found an increase in %TGW from 86 (SD 10) to 96 (SD 7) and 91.7 (SD 6.1) to 101.8% (SD 7.7) and 88 to 93.47%. One study reported weight change as 12/19 patients reaching 100%TGW at 3 months and the other 7/19 reaching a mean %TGW of 94% with mean %TGW at admission of 88%). The mean LOS varied between these studies from 3.2 weeks to 28.5 weeks.	⨁◯◯◯ VERY LOW	CRITICAL
		very serious ^a^	not serious	not serious	not serious	all plausible residual confounding would reduce the demonstrated effect	One study with total of 51 patients looking at EDE-Q. Fifty-three % of patients were referred directly from the inpatient unit in which case the treating inpatient clinician and insurance provider had to have determined that the patient/family required higher intensity treatment than outpatient could provide. Thirty-five % were referred due to inability to make progress in outpatient treatment. In 12% of cases, no referral source was recorded/available. Previous treatment and route of referral was not noted in other study. LOS was 7 weeks and mean of 40 +/− 17.2 days in each program. Global EDE-Q score decreased from 3.76 (SD 1.55) to 2.08 (SD 1.4) from admission to discharge (*p* = 0.001) in one study and from a mean of 3.83 +/− 0.95 to 1.50 (+/−1.03) in the other study (*p* = 0.012). Adolescent norm score reported in study was 1.6 (SD 1.4).	⨁◯◯◯ VERY LOW	IMPORTANT
		very serious ^a^	not serious	not serious	serious ^c^	all plausible residual confounding would reduce the demonstrated effect	Two studies for a total of 82 patients reported on change in EDI. Admission to the program was determined based on clinical assessment that the patients required a high level of treatment intensity based on symptomatology, in some cases patients had not received any prior treatment. LOS were 15 and 21.4 weeks. Change in EDI-2 was reported in one study and stated that EDI-DT decreased from 16.05 (SD 6.04) to 11.56 (SD 7.42) and EDI-BD decreased from 19.85 (SD 8.39) to 17.31 (SD 9.21), this study also reported that of those starting above the norm at beginning of study, 40% of patients improved on EDI-DT and 24.6% on EDI-BD). In the second study EDI-3 scores were reported to have improved significantly on all subscales other than maturity fears by 3 months. Scores for EDI-DT decreased from 49.24 (SD 12.61) to 42.06 (SD 11.52) and EDI-BD from 48.47 (SD 11.85) to 46.65 (SD 11.74).	⨁◯◯◯ VERY LOW	IMPORTANT
		very serious ^a^	not serious	not serious	serious ^c^	all plausible residual confounding would reduce the demonstrated effect	One study involved 56 patients, only 30 patients had pre-post data to analyze, mean LOS of 10.3 weeks. ChEAT scores reported only in graph format, all subscales significantly improved, although upper and lower confidence intervals overlapped with median effect in all subcales.	⨁◯◯◯ VERY LOW	IMPORTANT
		very serious ^a^	not serious	not serious	not serious	none	Completion rate - One study with 51 patients. Patients were referred from both inpatient and outpatient sources based on severity of symptoms. 15/36 patients (30%) were considered not successful (ie premature d/c) due to need for higher level of care, psychiatric hospitalization or left treatment AMA. Mean LOS was 22.2 (SD 3.8) days.	⨁◯◯◯ VERY LOW	CRITICAL
Change in EDE, YBC-EDS (assessed with: Pre/post YBC-EDS), Body Checking Questionnaire									
1	Case Report	very serious ^a^	not serious	not serious	serious ^c^	all plausible residual confounding would reduce the demonstrated effect	One study with 8 patients and their parents. LOS mean of 40 days +/−17.2. Intervention was family-based with CBT principles. EDE-Q subscales --statistically significant decreases in all subscales (range *p* = 0.012 to 0.028).	⨁◯◯◯ VERY LOW	
		very serious ^a^	not serious	not serious	serious ^c^	all plausible residual confounding would reduce the demonstrated effect	YBC-EDS total score decreased from mean 39.29 (+/−8.42) to 17.12 (+/−11.47) (*p* = 0.028), Concerns scores from mean of 15.57 to 9.43 (*p* = 0.034) and Rituals from mean of 14.71 to 7.71 (*p* = 0.028).	⨁◯◯◯ VERY LOW	
		very serious ^a^	not serious	not serious	serious ^c^	all plausible residual confounding would reduce the demonstrated effect	BCQ total scores decreased pre/post from 59.67 (+/−20.96) to 43.50 (+/−15.15) (*p* = 0.075). Scores also decreased for idiosyncratic checking and body dimensions subscales (*p* = 0.027 and 0.046)	⨁◯◯◯ VERY LOW	

Explanations

^a^Observational study with no control comparison

^b^Differences in admission BMI/%TGW, LOS, amount of hours/week of treatment which are likely to affect outcomes

^c^Confidence intervals wider than effect size in some studies

Bibliography:

Case Series - Girz 2013 [273], Henderson 2014 [275], Johnston 2015 [276], Grewal 2014 [274], Ornstein 2012 [272]

Case Report – Iniesta Sepulveda 2017 [277]

In terms of psychological symptoms, one study with total of 51 patients looked at EDE-Q scores and found improvements [276]. Two studies for a total of 82 patients reported on change in EDI [273, 275]. EDI drive for thinness subscale decreased in one study [275], and in the second study EDI scores were reported to have improved significantly on all subscales other than maturity fears by 3 months [273]. One study examining ChEAT scores, involved 56 patients, however, only 30 patients had pre-post data to analyze. The mean length of stay was 10.3 weeks [272]. ChEAT scores improved.

One study with 51 patients [276] examined a family therapy with group DBT skills training in an intensive outpatient program. Fifteen out of 36 patients (30%) were considered not successful due to need for higher level of care, psychiatric hospitalization or left treatment against medical advice.

One study was found with eight patients and their parents [277] describing family-based treatment with CBT principles within a DTP. Statistically significant decreases were seen in all subscales of the EDE-Q and the Yale Brown Cornell Eating Disorder Scale (YBC-EDS) total score decreased significantly [277] (Table 57).

##### Avoidant/restrictive food intake disorder

One study examined 32 patients with ARFID, compared to patients with AN (*n* = 68), BN (*n* = 15) and OSFED (*n* = 15) in the same DTP [278] (Table 58). This study reported that the reason for patients with ARFID to be admitted to their day treatment program was “acute onset of severe food restriction that results in significant weight loss or failure to gain weight.” Length of stay for ARFID patients was significantly shorter than for those with AN, but not compared to those with BN or OSFED. Patients with ARFID gained weight from 86% median BMI to 95% median BMI which did not differ from the median weight gain for the AN or OSFED groups (Table 58). This study also reported that patients with ARFID had total ChEAT scores that were subclinical at admission and demonstrated minimal change in scores during treatment. There were no significant differences between the diagnostic groups at the end of treatment on ChEAT scores [278].

**Table 58:** Family-based day treatment for children and adolescents with ARFID

Certainty assessment							Impact	Certainty	Importance
№ of studies	Study design	Risk of bias	Inconsistency	Indirectness	Imprecision	Other considerations			
Change in Weight (assessed with: Pre/post % median BMI), Change in ED symptomatology (assessed with: Pre/post ChEAT scores)									
1	Case Control	serious ^a^	not serious	not serious	serious ^b^	none	One study of 32 patients with ARFID, compared to patients with AN (*n* = 68), BN (*n* = 15) and OSFED (*n* = 15) in the same DTP. Study reported that the reason for patients with ARFID to be admitted to their PHP was “acute onset of severe food restriction that results in significant weight loss or failure to gain weight.” LOS for ARFID was significantly lower than AN (7.03 +/− 3.38 weeks vs 11.94 +/− 4.21 weeks), but not BN or OSFED. Patients with ARFID gained weight from 86.21%MBMI (+/− 9.96) to 95.45%MBMI (+/− 7.96) which did not differ from the median weight gain for the AN or OSFED groups.	⨁◯◯◯ VERY LOW	CRITICAL
		serious ^a^	not serious	not serious	serious ^b^	none	Patients with ARFID had Total ChEAT scores that were subclinical at admission and demonstrated minimal change in scores during treatment. There were no significant differences between the diagnostic groups at the end of treatment on ChEAT scores.	⨁◯◯◯ VERY LOW	NOT IMPORTANT

Explanations

^a^No control or comparison with no treatment, just patients in same program with other ED diagnoses

^b^Confidence intervals wide

Bibliography:

Case Control - Ornstein 2017 [278]

#### Family-based day treatment combined with dialectical Behavioural therapy

##### Bulimia nervosa

One study including 35 adolescent females with BN examined DBT combined with FBT principles within a day hospital setting [279] (Table 59). Length of stay was 77.18 days. Binge-purge symptoms monitored via self-report on EDE-Q decreased significantly [279]. EDE-Q global, shape and weight concerns decreased significantly pre-post, whereas restraint and eating concerns scores were unchanged at end of treatment [279].

**Table 59:** Family-based combined with DBT-based day treatment for children and adolescents with bulimia nervosa

Certainty assessment							Impact	Certainty	Importance
№ of studies	Study design	Risk of bias	Inconsistency	Indirectness	Imprecision	Other considerations			
Weight Change (assessed with: Pre-post BMI), Change in frequency of bingeing and purging (assessed with: Pre-post frequency of binge/purge symptoms), Change in EDE-Q (assessed with: Pre-post EDE-Q)									
1	Case Series	very serious ^a^	not serious	not serious	not serious	none	Study included 35 adolescent females. Criteria for referral/admission to the program was not reported. BMI did not change. At admission mean BMI was 26.3 (SD 2.34) and at discharge mean BMI was 24.9 (SD 2.87) (p 0.68). LOS 77.18 days.	⨁◯◯◯ VERY LOW	IMPORTANT
		very serious ^a^	not serious	not serious	serious ^b^	none	Study included 35 adolescent females. LOS 77.18 days. B/P symptoms monitored via self-report on EDE-Q reported as monthly frequency of these symptoms. At admission the mean frequency of objective bingeing was 4.03 (SD 6.69) and at discharge it was 1.43 (SD 3.66) (*p* = 0.04). At admission the self-reported (ie EDE-Q) mean frequency of purging was 10.82 (SD 11.57) and at discharge it was 3.51 (SD 2.26) (*p* = 0.005).	⨁◯◯◯ VERY LOW	CRITICAL
		very serious ^a^	not serious	not serious	not serious	none	EDE-Q global, shape and weight concerns decreased significantly pre-post (p 0.001–0.002). Restraint and eating concerns scores were unchanged at end of treatment.	⨁◯◯◯ VERY LOW	IMPORTANT

Explanations

^a^No comparison/control

^b^Wide confidence intervals, larger than actual effect

Bibliography:

Case Series - Murray 2015 [279]

#### CBT- based day treatment

##### Anorexia nervosa

One case series including 42 patients with AN examined a CBT- based day treatment program [280] (Table 60). Length of stay in day treatment was a mean of 22.2 weeks. Patients gained weight, with a mean increase of 5.37 kg or BMI increase of 1.87 kg/m over the course of treatment (Table 60). It was noted the increase in weight was correlated with the number of months in program, as well as EDI scores and Motivational Stages of Change score. Of note only 38 completed 2 months, 25 completed 4 months and 9 completed 6 months of treatment.

**Table 60:** CBT-based day treatment for children and adolescents with anorexia nervosa

Certainty assessment							Impact	Certainty	Importance
№ of studies	Study design	Risk of bias	Inconsistency	Indirectness	Imprecision	Other considerations			
Change in Weight (assessed with: Pre/post measures of weight)									
1	Case Series	serious ^a^	not serious	not serious	serious ^b^	none	One study, including 42 patients. Unclear reasons for patients being referred to the program. Mean duration of illness prior to admission to this program was 2.40 years (SD = 2.02). LOS in Day Treatment was a mean of 22.2 weeks (range 0–52 weeks). Patients gained weight, with a mean increase of 5.37 kg or BMI increase of 1.87 kg/m over the course of treatment. It was noted the increase in weight was correlated with the number of months in program (0.23, *p* < 0.01), EDI-DT (− 4.90, *p* < 0.001), EDI-BD (− 3.56, *p* < 0.001) and Motivational Stages of Change (6.15, *p* < 0.001). Of note only 38 completed 2 months, 25 completed 4 months and 9 completed 6 months -- unclear how many were discharged due to improved clinical presentation vs deterioration or inability to meet program requirements.	⨁◯◯◯ VERY LOW	CRITICAL

Explanations

^a^Observational study with no comparison or control group

^b^Confidence intervals not reported

Bibliography:

Case Series - Green 2015 [280]

#### Behaviour therapy based day treatment

##### Avoidant/restrictive food intake disorder

Two case reports were found describing patients aged 4 years (fear of choking) [281] and 8 years (emetophobia) [282]. Length of stay in the day treatment program was 9 days and 7 days respectively. At the end of treatment, the patients had increased their intake (Table 61). The 4 year old was no longer supplement dependent and accepting 30 new foods. The 8 year old had increased her intake from having nothing by mouth to meeting her daily nutritional needs.

**Table 61:** Behaviour therapy based day treatment for children with ARFID

Certainty assessment							Impact	Certainty	Importance
№ of studies	Study design	Risk of bias	Inconsistency	Indirectness	Imprecision	Other considerations			
Change in eating behaviours/intake (assessed with: Pre/post measures of intake)									
2	Case Reports	very serious ^a^	not serious	not serious	not serious	none	Two case reports, patients were 4 yrs. (fear of choking) and 8 yrs. (emetophobia). LOS in DTP were 9 days and 7 days respectively. At end of treatment the patients had increased their intake. The 4 yo was no longer supplement dependent and accepting 30 new foods. The 8 yo had increased her intake from NPO to meeting her daily nutritional needs.	⨁◯◯◯ VERY LOW	CRITICAL

Explanations

^a^Case studies only, no comparison/control

Bibliography:

Case Reports - Seiverling 2016 [281], Williams 2011 [282]

#### Resistance training as an adjunct in a day treatment program

##### Mixed diagnoses

This randomized controlled study involved 36 patients with mixed diagnoses of eating disorders (18 intervention and 18 control) [283]. The study took place within a day treatment program and consisted of supervised exercise (50–60 min), for 3 days per week for 8 weeks. In order to be included in the study the patients must have had a BMI greater than 14 and could not be “excessive exercisers” (ie < 6 h per week). Intervention patients received resistance training plus 150 kcal extra to compensate for this activity. There was no difference in weight restoration between groups (Table 62).

**Table 62:** Resistance training in combination with day treatment for adolescents with eating disorders

Certainty assessment							Impact	Certainty	Importance
№ of studies	Study design	Risk of bias	Inconsistency	Indirectness	Imprecision	Other considerations			
Body Mass Index at Discharge (assessed with: BMI calculated)									
1	randomised trials	serious ^a^	not serious	not serious	not serious	none	36 patients participated (18 intervention and 18 control) another 8 patients were lost to follow-up. Study took place within a day treatment program and consisted of 3 day per week × 8 weeks of supervised exercise (50–60 min). In order to be included in the study the patients must have had a BMI > 14 kg/m and could NOT be “excessive exercisers” (ie < 6 h /week). Intervention patients received resistance training + 150 kcal extra to compensate for this activity. There was no difference in weight restoration between groups. Mean BMI at initiation of study ranged was greater than 17 in both groups and patients had already been hospitalized for a mean of 50.8 and 61.5 days prior to enrollment in the study. Exclusion factor - excessive exercise as part of illness.	⨁⨁⨁◯ MODERATE	IMPORTANT

Explanations

^a^No concealment or blinding for patients or study team noted

Bibliography:

RCT - Fernandez-del-Valle 2016 [283]

### Residential treatment

Four case series examined residential treatment and included 1068 patients with AN, BN and EDNOS, along with two additional case reports (Table 63). One case series examined patients with AN exclusively [287]. Reasons for admission to residential treatment were not noted and all studies took place in the United States. These studies measured change in weight in various ways. Four studies utilized BMI [284–287]. Admission mean BMI varied from 15.8 to 18.6. Discharge mean BMI varied from 17.8 to 21.3. Change in mean BMI from admission to discharge varied from 1.92 to 2.72. Two studies additionally reported on %TGW at admission and discharge. Admission mean %TGW were 83.4% [284] and 76.7% [287] and discharge mean %TGW were 94.7 and 86.6% respectively.

**Table 63:** Residential treatment for children and adolescents with eating disorders

Certainty assessment							Impact	Certainty	Importance
№ of studies	Study design	Risk of bias	Inconsistency	Indirectness	Imprecision	Other considerations			
Change in Mean Body Mass Index at Discharge (assessed with: Calculated BMI), change in purge frequency, EDI 3 Drive for thinness, EDE-Q, Readiness for Change									
4	Case Series	very serious ^a^	serious ^b^	not serious	serious ^c,d^	none	Studies included patients with AN, BN and EDNOS for a total *n* = 1068. Reasons for admission to residential treatment were not noted and all studies took place in the US (ie decision for admission likely influenced by insurance coverage/parental finances). One study noted that they included only data from the first admission for patients admitted more than once to residential treatment and that only patients who remained in treatment > 2 weeks were included. Another study noted that patients had a mean of 1.2 previous inpatient admissions prior to residential treatment. Otherwise there was a paucity of information describing previous treatments. They measured change in weight in various ways. Four studies utilized BMI. Admission mean BMI varied from 15.8 to 18.65. Discharge mean BMI varied from 17.8 to 21.3. Change in mean BMI from admission to discharge varied from 1.92 to 2.72. Two studies additionally reported on %TGW at admission and discharge. Admission mean %TGW were 83.4 and 76.7% and discharge mean %TGW were 94.7 and 86.6% respectively.	⨁◯◯◯ VERY LOW	CRITICAL
		very serious ^e^	serious ^f^	not serious	serious ^d^	all plausible residual confounding would reduce the demonstrated effect	One study reported on 361 patients that were purging at admission a mean of 3.25 times per day. At discharge they were purging a mean of 0.02 times per day. Differing diagnostic groups not reported separately. LOS 51.8 days +/− 25.8. Treatment was multimodal.	⨁◯◯◯ VERY LOW	IMPORTANT
		very serious ^g^	serious ^b^	not serious	serious ^c^	all plausible residual confounding would reduce the demonstrated effect	Three studies looked at EDI 3 scores and included 313 patients with AN, BN and EDNOS. Treatment provided was multimodal. Various subscales and EDI-3 Risk Composite as well as EDI-3 Global were reported in the some of the studies. EDI-3 Risk Composite was reported in 2 studies (total *n* = 212) mean decrease in EDI-3 RC varied from 14 to 31 (SD = 23.1 and 17.62 respectively). EDI-3 Global was reported in 1 study (*n* = 101) where it decreased a mean of 39.3 points (SD = 55.2). EDI-3 Drive for Thinness was reported in 3 studies (*n* = 277) where it decreased a mean of 3.53, 5.11 and 12.37 (SD 6.9, 7.81 and 6.42). EDI-3 was reported in 1 study (*n* = 111) and decreased 3.75 (SD = 2.21). EDI-3 Body Dissatisfaction was reported in 1 study (*n* = 101) where it decreased 3.45 (SD = 10.88). LOS varied between studies from 28.5–56.4 days, one study did not report their LOS.	⨁◯◯◯ VERY LOW	CRITICAL
		very serious ^g^	not serious	not serious	not serious	all plausible residual confounding would reduce the demonstrated effect	One study looked at the EDE-Q Pre to Post and included 105 patients with AN, BN and EDNOS. Treatment was multimodal and mean LOS was 56 days. EDE-Q changed from 3.6 (SD = 1.58) to 1.95 (SD = 1.35), mean change − 1.56 (SD = 1.27) -- similar to reported norms in adolescent girls.	⨁◯◯◯ VERY LOW	CRITICAL
		very serious ^c,g^	not serious	not serious	not serious	all plausible residual confounding would reduce the demonstrated effect	One study included 65 patients with AN and treatment was multimodal. Mean readiness for change (ANSOQC) at admission was 53.98 (SD 16.36) and at discharge was 67.28 (SD 20.06). This difference was statistically significant, but signifies no change in actual stage of change (ie Preparation Phase scores are 50–69). They were further divided into low readiness and high readiness. High readiness patients had a shift from 66.86 (SD 11.78) at admit to 76.80 (SD 15.71) at d/c, signifying a shift from Preparation to Action Phases. Low readiness patients shifted from 40.70 (SD 7.12) to 57.47 (SD 19.5), signifying shift from Contemplative to Preparation Phases. LOS was 28.5 days.	⨁◯◯◯ VERY LOW	IMPORTANT
Weight gain									
2	Case Reports	very serious ^g^	not serious	not serious	not serious	none	The 2 case reports both described patients with Type 1 diabetes and reported weight gains of 2.2 and 4.3 kg during admission. Varying types of treatment provided in multimodal format. LOS varied among studies from 28 days to 56 days and in one study LOS was not reported.	⨁◯◯◯ VERY LOW	IMPORTANT

Explanations

^a^Observational study with no comparison

^b^Mixed diagnostic group (AN, BN and EDNOS)

^c^Large or overlapping confidence intervals wide in some studies included here

^d^Confidence intervals not reported or not reported in all studies

^e^Observational study with no comparison, self-reported # of purges/day

^f^Mixed diagnostic group (AN-B/P and BN) - results not differentiated

^g^Observational study with no comparison, self-rated scale

Bibliography:

Case Series - Fisher 2015 [284], Weltzin 2014 [285], Twohig 2016 [286], McHugh 2007 [287]

Case Reports – Pitel 1998 [288], Rodigue 1990 [289]

One study reported on 361 patients that were purging at admission a mean of 3.25 times per day [284]. At discharge, they were purging a mean of 0.02 times per day. Differing diagnostic groups were not reported separately. Length of stay was an average of 52 days. Treatment was multimodal.

In terms of psychological symptoms, three studies looked at EDI scores and included 313 patients with AN, BN and EDNOS [285–287]. The treatment provided was multimodal. Length of stay varied between studies from 28.5 to 56.4 days. In general, EDI scores were improved when admission scores were compared with discharge scores. One study looked at the EDE-Q pre to post and included 105 patients with AN, BN and EDNOS [285]. The EDE-Q changed from 3.6 (SD = 1.58) to 1.95 (SD = 1.35).

One study including 65 patients with AN examined readiness for change. Treatment was multimodal [287]. Mean readiness for change (ANSOQC) at admission was 53.98 (SD 16.36) and at discharge was 67.28 (SD 20.06). This difference was statistically significant, but does not signify a change in actual stage of change. Participants were further divided into low readiness and high readiness. High readiness patients had a shift from 66.86 (SD 11.78) at admission to 76.80 (SD 15.71) at discharge, signifying a shift from Preparation to Action Phases. Low readiness patients shifted from 40.70 (SD 7.12) to 57.47 (SD 19.5), signifying a shift from Contemplative to Preparation Phases.

Two case reports both described patients with AN and Type 1 diabetes and reported weight gains and better glycemic control after residential treatment [288, 289]. Varying types of treatment were provided in multimodal format.

## Recommendations

### Family therapy

#### Family-based treatment

##### Family-based treatment (FBT) is strongly recommended for any child or adolescent with Anorexia Nervosa or Bulimia Nervosa, especially for those who have been ill less than 3 years.

**Strong recommendation**

*Qualifying statements:*

There are implementation challenges with Family-Based Treatment (FBT) including requirements for specialized, well-trained staff, access and costs of training. Parent-Focused Family Therapy – where the patient is seen separately from the family – may be just as effective as traditional FBT where the family is seen together. Adaptations to FBT such as shorter or longer treatment, removal of the family meal, guided self-help, parent to parent consult, short term intensive formats, and delivery of FBT by telehealth, require more study. Structural and Systemic Family therapy might be helpful for children and adolescents with Anorexia Nervosa, but the evidence generally does not indicate superiority to FBT, especially when costs are taken into consideration.

*Key Evidence:*

Anorexia Nervosa

One meta-analysis [21] and three high quality RCTs [6, 22, 23] have demonstrated that greater weight gain and higher remission rates are achieved in FBT compared to individual treatment, particularly when focusing on one year follow-up. Eight large case series also show improvement in weight following treatment [26–32, 40].

Bulimia Nervosa

Three high quality RCTs for Bulimia Nervosa have been completed and compared FBT to various control conditions [48–50]. When FBT was compared to Cognitive Behavioral Therapy (CBT), remission rates were significantly higher in the FBT group (39% versus 20%) [50]. Remission rates were also significantly better in the FBT group, when FBT was compared to supportive psychotherapy (39% versus 18% )[48]. However, when family therapy (with some elements consistent with FBT) was compared to guided self-help CBT, there were no significant differences in remission (10% versus 14%) [49]. A case series and case report also support the use of FBT for Bulimia Nervosa [34, 51].

#### Multi-family therapy

##### Multi-family therapy (MFT) may be a reasonable treatment option for children and adolescents with Anorexia Nervosa.

**Weak recommendation**

*Qualifying statements*:

Multi-Family Therapy (MFT) provides workshops for multiple families at once and generally is delivered alongside single-family therapy following FBT principles, although some studies just report on the delivery of the multi-family workshops alone. It may be challenging for programs to run MFT as it requires several staff present for several full days and requires several families interested at the same time to begin the treatment. The delivery of MFT for children and adolescents with Bulimia Nervosa may be beneficial but requires more study. Members of the guideline committee indicated that the value of parents having support from each other cannot be understated. The panel voiced that peer support is often a missing component of treatment and hospital administration can place barriers to the implementation of this option.

*Key evidence:*

One large high quality RCT found that MFT (multi-family workshops plus single FBT) conferred additional benefits compared to FBT alone in terms of remission rates in adolescents with Anorexia Nervosa (75% in MFT versus 60% in FBT) [76]. Several case series have also demonstrated a benefit of MFT [77–80]. There is one small case series examining MFT for adolescents with Bulimia Nervosa which found improvements in eating disorder symptoms [81].

#### Additional promising therapies

Other outpatient family therapies exist that have some data showing their promise but where more research is required before definitive recommendations can be made. These are treatment options in which research efforts should be prioritized.

They are:

- FBT for children with atypical Anorexia Nervosa.
- FBT for children with Avoidant/Restrictive Food Intake Disorder (ARFID).
- FBT for children across the gender spectrum, including individuals who are gender variant or gender non-conforming.
- Adjuncts to FBT, such as cognitive remediation therapy, art therapy and cognitive behavioural therapy for children and adolescents with Anorexia Nervosa.
- Emotion focused family therapy (EFFT) for Bulimia Nervosa and Anorexia Nervosa, as stand- alone treatment, or as an adjunct to FBT.

### Individual or group outpatient psychotherapies

#### Cognitive Behavioural therapy

##### Cognitive behavioural therapy may be a reasonable treatment option for children and adolescents with Anorexia Nervosa or Bulimia Nervosa.

**Weak recommendation**

*Qualifying statements:*

Across the studies, Cognitive Behavioural Therapy was not offered in a uniform manner. Motivational interviewing as a component of treatment or prior to initiating treatment, may also be helpful although strong scientific evidence is lacking due to a paucity of studies.

*Key evidence:*

Anorexia nervosa

Eight case reports [97–104] and one large case series [96] indicate that CBT results in weight gain and improvement in eating disorder psychological symptoms for children and adolescents with Anorexia Nervosa. A small RCT (*n* = 22) did not show any difference between CBT and Behavioural Family Therapy in terms of these outcomes for children with Anorexia Nervosa, however, both improved [24]. Efficacy has also been shown when CBT is delivered in a group setting for Anorexia Nervosa [105, 106].

Bulimia nervosa

For Bulimia Nervosa, three high quality RCTs exist examining CBT. One RCT compared CBT to psychodynamic therapy in primarily adolescents, but also some young adults. This trial did not find any difference in terms of remission from Bulimia Nervosa. There were small advantages in terms of greater reduction in binge/purge frequency in the CBT group [107]. There are also two high quality RCTs comparing CBT to family-based approaches for Bulimia Nervosa [49, 50]. There are conflicting results between these two studies, with the study by Le Grange and colleagues [50] indicating significantly greater remission rates in the FBT group compared to the CBT group, whereas the study by Schmidt and colleagues [49] showed no significant difference between the groups with only a small proportion remitted in each group. Two large case series indicate significant decreases in binge/purge frequency pre to post treatment [108, 109]. Several case reports indicating improvement in binge/purge symptoms exist [110–114].

#### Adolescent focused psychotherapy

##### Adolescent focused psychotherapy may be a reasonable treatment option for children and adolescents with Anorexia Nervosa.

**Weak recommendation**

*Qualifying statements:*

Adolescent Focused Psychotherapy (AFP) could be delivered in situations in which FBT has been attempted, but been ineffective, or if FBT is contraindicated, not possible, or not available.

A manual is not yet available to clinicians, which makes training and dissemination difficult.

It is a challenge to study this type of treatment due to its lengthy nature and lack of clarity around essential elements. Adolescent Focused Psychotherapy includes elements of: an emphasis on therapeutic relationship with a goal to improve symptoms, psychoeducation, the role of the eating disorder as a coping mechanism, along with the development of more positive coping mechanisms. Panel members agreed that treatment of this nature is commonly delivered and can be quite beneficial to some patients. This treatment for Anorexia Nervosa may be beneficial, however other treatments have some advantages in terms of cost and more rapid improvement in symptoms.

*Key evidence:*

Anorexia Nervosa

Adolescent Focused Psychotherapy (AFP; based on psychodynamic principles) has some evidence to support its use [22, 23, 128], as does individual psychodynamic treatment [129], and group analytic psychotherapy [130]. Remission rates were not significantly different between AFP and FBT in two RCTs involving a total sample of 158 adolescents [22, 23]. Rates of 20% (12/60) remitted in AFP compared to 34% (21/60) in FBT were found in the study by Lock and colleagues [23], whereas 41% in the AFP group met the weight goal of the 50th percentile in the study by Robin and colleagues [22] compared to 53% in the FBT group. Differences between FBT and AFP become more apparent at one year follow up with FBT having an advantage [23].

#### Additional promising psychotherapies

Other promising outpatient psychotherapies exist that require more research before definitive recommendations can be made.

These include:

- Cognitive Behavioural Therapy for Avoidant/Restrictive Food Intake Disorder.
- Dialectical Behavioural Therapy for eating disorders.

#### Other therapies - adjunctive yoga

##### Yoga, in addition to standard treatments, may be a reasonable option for medically stable youth with Anorexia Nervosa, Bulimia Nervosa, and Other Specified Feeding and Eating Disorders.

**Weak recommendation**

*Qualifying statements:*

There is no evidence to guide the specific regimen (e.g. duration, frequency) of yoga. Yoga should only be undertaken with support by the physician involved in the individual’s care. Hot yoga or other strenuous forms of Yoga are not recommended when medical concerns exist. If Yoga interferes with recovery, or worsens symptoms, it should be discontinued.

*Key Evidence:*

One high quality study suggests some benefits in terms of the psychological symptoms of eating disorders, as well as depressive and anxious symptoms in the context of an eating disorder [136].

### Medications

The clinical trials environment to test medications for the treatment of eating disorders is fraught with ethical and methodological complexity. Obtaining the required informed consent to bring a child or adolescent into a study requires disclosure of the study intent, hypotheses, and potential for side effects attributable to the medication. In some cases, these effects (e.g. weight gain) are connected specifically to outcomes patients may strongly fear. In addition, parents are often reluctant to give their children psychotropic medication. This often results in studies that have prolonged enrollment phases, that struggle or fail to meet recruitment goals, and suffer from high rates of patient drop out. As a consequence, study quality is poor and prone to bias.

#### Atypical antipsychotics

##### Olanzapine or aripiprazole may be reasonable treatment options for certain populations of children and adolescents with Anorexia Nervosa if monitored carefully.

**Weak recommendation**

*Qualifying statements:*

In specific contexts, consideration of olanzapine and aripiprazole use may be undertaken for the adjunct treatment of low weight children and adolescents with Anorexia Nervosa. Although the evidence-base supporting these specific medications is scant and of poor quality, expert opinion suggests potential benefit in carefully selected treatment contexts. Given their propensity for side effects, these medications should only be considered with appropriate consultation and monitoring by trained specialists in Child and Adolescent Psychiatry or Pediatrics who have expertise in the treatment of children and adolescents with eating disorders. When utilized, these medications should be initiated at a very low dose (0.625–1.25 mg for olanzapine, or 0.5–1.0 mg for aripiprazole) and titrated very carefully. Target doses in research trials are often modest. Informed consent from the young person, or their substitute decision maker including risk of side effects must be obtained and appropriate monitoring undertaken while these medications are in use.

*Key evidence:*

Olanzapine

Olanzapine has been the most commonly studied psychotropic medication for children and adolescents with Anorexia Nervosa. At present, only one small double-blind placebo-controlled trial in this population has been published [137], and no beneficial effect in favour of olanzapine was found in the 15 subjects who completed the trial. Several open trials and case series have examined the use of olanzapine in children and adolescents with Anorexia Nervosa [138–142]. While some have demonstrated benefit (e.g. weight gain), reported adverse effects associated with the medication as well as patient attrition were common.

Aripiprazole

Three small poor-quality studies found aripiprazole showed some modest benefit in adolescents with Anorexia Nervosa [165–167].

#### Additional promising medications

The use of other medications for the purposes of eating disorder treatment require more research before definitive recommendations can be made. These medications should be a priority for research. These include:

- Selective Serotonin Reuptake Inhibitors (fluoxetine for Bulimia Nervosa).
- Risperidone and Quetiapine for use in Anorexia Nervosa.
- Atypical Antipsychotics for use in Avoidant/Restrictive Food Intake Disorder.
- Mirtazapine use for patients with Anorexia Nervosa.

#### Medications that are not recommended

The medications below have no evidence to support their use in the treatment of primary eating disorder symptoms, or are harmful.

- Selective Norepinephrine Reuptake Inhibitors – no evidence.
- Mood Stabilizers - no evidence.
- Buproprion - not recommended for use in eating disorders, due to the elevated risks of seizures in this population.

### Level of care – inpatient/day treatment/residential care

In contrast to the above sections that examine specific treatment modalities, this section focuses on the level – or setting - where care takes place. Research on level of care is generally sparse. Moreover, the setting where care takes place is often conflated with the treatment activities themselves making it difficult to attribute which mechanism(s) contributed to outcomes. Some tools already exist to guide the practitioner on which level of care might be indicated (e.g. residential, inpatient, day treatment, or outpatient care) based on a variety of clinical factors [290].

#### Level of care

##### It is strongly recommended that the least intensive treatment environment be provided (e.g. family-based treatment or day treatment versus lengthy hospitalizations) especially for those children and adolescents with Anorexia Nervosa requiring a first admission to hospital and/or with a duration of illness less than 3 years.

**Strong recommendation**

*Qualifying statements:*

Definitive clinical research does not currently exist that identifies the specific characteristics of what comprises “least intensive environment” or an agreed upon hierarchy of least to most intensive environments. However, the evidence-base does provide signals of reasonable options and areas that should be prioritized for further study. In addition, definitive clinical research does not currently exist that identifies the specific elements required to optimize inpatient, specialist outpatient, and community outpatient programs.

*Key evidence:*

Studies comparing different levels of care and length of stay

One trial of 167 adolescents randomized to inpatient care, a specialist outpatient program, or a generalist community outpatient program found significant improvements across all three groups with no differences between the groups [291]. In order to examine length of inpatient treatment related to outcome, two high quality studies examined the difference between patients randomized to receive a relatively short inpatient admission followed by either 20 sessions of FBT (*n* = 82) [252] or day treatment (*n* = 172) [253] compared to a lengthy inpatient stay to weight restoration. In the inpatient/FBT study [252] patients had all been unwell less than 3 years, and in the inpatient/day treatment study [253] the patients were included only if it was their first admission. At the end of FBT or day treatment, there were no significant differences between those who were discharged after a short admission versus those who remained in hospital for weight restoration in terms of: weight outcome, rate of readmissions over 12-month follow-up, or eating disorder symptoms.

Studies examining inpatient treatment only

Multiple low-quality studies have been published examining the outcomes for children and adolescents with eating disorders [187, 188, 193, 194, 197, 213, 216, 222, 224, 227, 232–234, 251, 292]. The most consistent finding is that inpatient treatment leads to weight restoration regardless of the treatment framework used. There are no studies directly comparing treatment modalities. Outcomes related to the cognitive symptoms of the eating disorder were mixed in these inpatient studies. Some low quality studies have examined various adjuncts to inpatient treatment including non-select versus selective menus, meal support, multi-family versus multi-parent group therapy, cognitive remediation therapy, and bright light therapy. Non-select menus conferred a benefit related to rate of weight restoration and meal support appeared to decrease the need for nasogastric tube feeds. Other outcomes were less evident and potentially eclipsed by the effect of the inpatient treatment milieu.

Studies examining day treatment programming only

Several low-quality studies report a benefit of day hospital programming in terms of weight restoration and reduction in eating disorder symptoms [255, 256, 258, 259, 262, 266, 272–276, 280]. Of these studies 14 described using a family-based approach [262, 264–270, 272–277], eight a multimodal approach [254–261] and one a CBT framework [280] in their programs. All studies reported an increase in mean weight during day treatment, and most studies reported improvement in eating disorder symptoms. One study compared cohorts in their program with and without family involvement [264], and found that weight outcomes did not vary with family involvement, but there was a greater improvement observed in overall symptomatology, and in particular weight concerns and dietary restraint with family involvement. Only three small studies have examined the use of day treatment settings for patients with Avoidant/Restrictive Food Intake Disorder [278, 281, 282] and one small study examined this setting for Bulimia Nervosa [279]. One high quality study observed no harm with the addition of a standardized resistance training program to day treatment care as usual for patients with mixed eating disorder diagnoses [283].

Studies examining residential programming only

Six low quality studies examined outcomes for patients with eating disorders treated in a residential setting [284–289]. Studies included patients with Anorexia Nervosa, Bulimia Nervosa and Eating Disorder Not Otherwise Specified for a total of 1070 patients studied. Reasons for admission to residential treatment were not noted, there is a paucity of information reported on treatments received prior to admission to residential facilities, and all studies took place in the United States. Length of stay in these studies varied from 28 days to 56 days. All six studies reported that underweight patients gained weight during treatment. One study reported that episodes of purging were significantly reduced [284]. Three studies reported that eating disorder symptoms decreased significantly during treatment in the residential setting [285–287].

#### General care considerations when choosing level or setting of treatment

The following are reasonable care considerations as it relates to the choice of environment in which treatment is available for children and adolescents with eating disorders.

##### Care within an inpatient treatment environment

- Inpatient treatment may promote weight restoration regardless of model of care provided, but requires more study to determine the critical treatment elements related to weight restoration.
- Cognitive Behavioural Therapy (CBT) and family-based inpatient treatment may lead to improvement in eating disorder symptoms.
- Inpatient treatment combined with day treatment follow-up may be helpful in weight restoration, symptom change and motivation for children and adolescents with Anorexia Nervosa.
- Adjuncts to inpatient treatment, such as non-selective menus, meal support, cognitive remediation and bright light therapy may be helpful for certain children and adolescents with eating disorders.
- Inpatient treatment alone or in combination with day treatment for Bulimia Nervosa and Avoidant/Restrictive Food Intake Disorder requires more study.
- Peer support during inpatient treatment by other parents would be an asset.

##### Preparing for discharge from inpatient care

- Any transition in care is a period of high risk for deterioration and adverse events. Bridging these transitions with added supports is needed to prevent young people from suffering from adverse outcomes due to disruptions in continuity of care.
- Parental support is needed in order to prepare parents for discharge and the treatment that follows.
- Patient and parent preferences should be considered when planning for discharge.
- Issues of consent and capacity should also be considered when making decisions around admission and discharge.

##### Care within a day treatment environment

- Day treatment may promote weight restoration in Anorexia Nervosa regardless of model of care provided, but requires more study to determine the critical treatment elements related to weight restoration.
- Multimodal, CBT and family-based day treatment may lead to improvement in eating disorder symptoms.
- Day treatment for Avoidant/Restrictive Food Intake Disorder may be helpful in weight restoration and improved outcome.
- Resistance training may be offered to children and adolescents who do not have a history of compulsive exercise while participating in day treatment, but it remains unclear whether this intervention imparts any benefit.
- Day treatment varies significantly from study to study, so comparison is difficult. The common element appears to be a group-based treatment program with meal support.
- Equity and access to day treatment are issues to be considered. Families must live close to such a program in order to be able to attend, or must abandon their home/career to move close to a day treatment program in order for their child to attend.

##### Care within a residential treatment program

- Although literature was lacking to support a formal recommendation for residential treatment, many of the panel members opined that residential treatment is an essential component of treatment for some individuals with eating disorders who need lengthier treatment in a setting away from home. Based on expert opinion and those with lived experience, it was agreed that individuals who have had repeat admissions to the hospital and those with complex comorbid conditions, might benefit from residential treatment.

## Discussion

These are the first Canadian Practice Guidelines to evaluate the evidence on psychotherapeutic and psychopharmacological treatments focused specifically on children and adolescents with eating disorders. Strong recommendations were supported in favour of Family-Based Treatment, and more generally in terms of least intensive treatment environment. Weak recommendations in favour of Multi-Family Therapy, Cognitive Behavioural Therapy, Adolescent Focused Psychotherapy, adjunctive Yoga, and atypical antipsychotics were confirmed. Several gaps for future work were identified including enhanced research efforts on new adjunctive treatments in order to address severe eating disorders and complex co-morbidities. Underlying the specific treatments emerged some general values and philosophies to be upheld, particularly apparent during the panel meeting. These philosophies included mutual trust and respect in the provider/patient/family relationship.

In addition, parent and patient representatives mentioned the critical importance of peer support (patient and parent), particularly in times of transition between different levels of care and from the pediatric to adult system of care. The importance of a co-ordinated continuum of care from outpatient to residential care was emphasized by the panel. The lack of services was also emphasized. Several individuals mentioned the lack of residential care across the country and the great need that exists for certain individuals with eating disorders for intensive inpatient and residential services. This need is particularly apparent for those who are medically stable, but have psychiatric co-morbidities and need longer term treatment in a highly structured environment. The co-morbidity with substance abuse was mentioned as an area where there is a complete lack of services in Canada. Patient and parent choice/preferences of treatment were also mentioned as essential to consider when thinking of the treatments and levels of care available.

The strengths of this guideline are numerous. We used rigorous methodology for our literature review and synthesis as well as for our guideline development. Our literature synthesis methods included a thorough review of all literature (including gray literature and papers of any language). We translated 25 papers for full text review. In terms of guideline development, conflict of interest statements were reviewed by an impartial chair in order to address any biases. We had a face-to-face meeting to discuss our recommendations, followed by an anonymous voting procedure. Furthermore, our panel included the voices of various stakeholder groups including researchers, clinicians, policy makers, parents and those with lived experience.

### Limitations

These guidelines serve as a starting point for Canadian Practice Guidelines for treating children and adolescents with eating disorders, and as such, they have several limitations. Our guidelines did not aim to review treatments within the scope of medical stabilization, or in terms of treatments for the physical complications of eating disorders in children and adolescents. A companion Canadian Guideline focused on these topics for children and adolescents is needed. The reader is encouraged to examine the Academy for Eating Disorders Guidelines on eating disorders, and the Clinical Practice Guidelines *for the* BC eating disorders continuum of services which includes a Short Term Allocation Tool for Eating Disorders (STATED) [290], specifically outlining medical criteria for hospital admission, and level of care recommendations for the full age spectrum. None of the outpatient treatments mentioned in our current guideline should be delivered with a medically unstable child needing hospital admission for medical reasons. Similarly, if outpatient treatments are attempted, and an individual deteriorates during these treatments, or fails to progress, stepping up to either day treatment, or inpatient care may be needed. Furthermore, if outpatient treatments are not available, then lengthier inpatient stays may be necessary.

We did not examine qualitative literature in our search. The scope of our guideline was so broad already, that these studies could not be incorporated. These studies should be included in future iterations of these guidelines. Qualitative meta-syntheses on the topic of treatment for and recovery from AN in particular, highlight the importance of therapeutic alliance, treatment targets, building identity and self-acceptance [293, 294]. These qualitative works, can shed light on the concept of recovery which can have several different definitions, not just focused on symptomatic change, but quality of life, and functionality of work and relationships. For the purposes of this guideline, we focused on studies reporting on symptomatic change, however, future iterations should include other outcomes as viewed as essential to those affected by these illnesses and their families. Caregiver outcomes would also be important to include in future guidelines. We also would recommend including transition age youth as an important population with unique needs. A more in-depth examination of transitions in the health care system in general would be beneficial.

Most of the published studies to date on pharmacotherapy of eating disorders in children and adolescents have focused on the role of antipsychotic medication in AN. Despite progress in recent years, the total number of subjects studied remains small, and there is a paucity of randomized controlled trials. Further, it has become increasingly clear that there are substantive challenges involved with the completion of such studies. As a result, there is still insufficient evidence to recommend medication as a first line consideration in children and adolescents with eating disorders. Due to the significant challenges in recruitment and retention in clinical trials to date, large multi-site collaborative trials are necessary to move the field forward in determining which young patients with eating disorders might benefit most from psychotropic medication and in what fashion. In addition, we did not systematically review the literature for certain classes of medications including benzodiazepines, or stimulants. We came across a couple of case reports through searching in the other areas [295, 296], however, searches on these drugs should be included in future iterations of this guideline.

Our search strategy also had limitations. Although our search was very thorough, we were unable to retrieve several citations as full text articles. Some of these were difficult to locate as they were dissertations, conference proceedings, books, or simply did not exist. We attempted to examine sex differences, but the numbers of male subjects were so small that no conclusions could be drawn. Furthermore, although we searched the literature thoroughly for art and music therapies, we could not find any articles on these topics. In addition, two papers in the family-based therapy section were identified through external review, indicating that these papers were not found through the initial search.

Despite these limitations, these guidelines represent a significant step forward in developing a collaborative process for identifying effective treatments for children and adolescents with eating disorders and will be reviewed every 5 years.

### Future directions

Several gaps were noted by the guideline panel and these should be a focus for future study. These gaps included treatments for complex presentations of eating disorders, including complex co-morbidity such as borderline personality disorder, obsessive compulsive disorder, and substance use disorder. Determining which treatment benefits which individual in advance should be a priority for further study. There were also difficulties in making recommendations around medication use. Studies in the area of psychopharmacology are fraught with challenges in terms of a lack of recruitment and retention. Small and poorly designed studies, make it difficult to arise at recommendations. Perhaps multi-site trials, or innovative designs are needed to further promote and enhance the evidence base where psychopharmacology is concerned. The panel noted difficulty in making recommendations on inpatient and residential levels of care, but noted that these are sorely needed services, and should be expanded in Canada, along with a more rigorous investigation of effectiveness. Developing treatments, including new and adjunctive psychotherapeutic approaches for families unable to engage in Family-Based Treatment is essential. In addition, particular populations may have unique needs that have not yet been explored, such as predominantly male populations, and those with non-binary gender identities. Furthermore, creative ways of accessing evidence-based treatment need to be explored including the use of technology to treat patients and families at a distance.

## Conclusions

Our Canadian Practice Guidelines for the treatment of children and adolescents with eating disorders recommend the provision of: 1) FBT for those with AN or BN (strong recommendation), 2) MFT for those with AN (weak recommendation), 3) CBT for those with AN or BN (weak recommendation), 4) AFP for those with AN (weak recommendation), and, 5) adjunctive Yoga for those with AN, BN and OSFED (weak recommendation). All of these treatments can only be delivered in a medically stable young person, and more intensive treatment should be considered if treatments are deeming to lack efficacy. In terms of medication, a weak recommendation was confirmed for olanzapine and aripiprazole for those with AN. A strong recommendation was agreed upon for providing care in a least intensive environment. Patient and parental preferences should be considered. Research efforts should be devoted to developing treatments for severe eating disorders with complex co-morbidity.

## Data Availability

Not applicable.

## Abbreviations

%TGW: Percent treatment goal weight
ADHD: Attention deficit hyperactivity disorder
AFP: Adolescent focused psychotherapy
AGREE II: Appraisal of guidelines, research, and evaluation
AN: Anorexia nervosa
ANSOCQ: Anorexia nervosa stage of change questionnaire
ARFID: Avoidant/restrictive food intake disorder
BED: Binge eating disorder
BLT: Bright light therapy
BMI: Body mass index
BN: Bulimia nervosa
CBT: Cognitive behavioural therapy
CBT-E: Cognitive behavioural therapy - enhanced
ChEAT: Children’s eating attitudes test
CRT: Cognitive remediation therapy
DBT: Dialectical behavioural therapy
DTP: Day treatment program
EAT: Eating attitudes test
EDE: Eating disorder examination
EDE-Q: Eating disorder examination - questionnaire
EDI: Eating disorders inventory
EDNOS: Eating disorder not otherwise specified
EFFT: Emotion focused family therapy
FBT: Family-based treatment
GDP: Guideline development panel
GRADE: Grading of recommendations assessment, development, and evaluation system
GUIDE-M: Guideline implementability for decision excellence model
LOS: Length of stay
MFT: Multi-family therapy
MPT: Multi-parent group therapy
MSCARED: Motivational stages of change for adolescents recovering from an eating disorder
NGY: Nasogastric tube
NJ: Nasojejunum
OCD: Obsessive compulsive disorder
OSFED: Other specified feeding and eating disorder
PRISMA: Preferred reporting items for systematic reviews and meta-analyses
SNRIs: Selective norepinephrine reuptake inhibitors
SSRIs: Selective serotonin reuptake inhibitors
TAU: Treatment as usual
YBC-EDS: Yale brown Cornell eating disorder scale
